# Phospholyl and Arsolyl Triple-Decker Sandwich Complexes of Europium(II) and Strontium(II)

**DOI:** 10.1021/jacsau.4c00300

**Published:** 2024-06-06

**Authors:** Noah Schwarz, Julia Feye, Vanitha R. Naina, Ralf Köppe, Sebastian Gillhuber, Xiaofei Sun, Peter W. Roesky

**Affiliations:** † Institute of Inorganic Chemistry, Karlsruhe Institute of Technology, Kaiserstr. 12, 76131 Karlsruhe, Germany; ‡ Faculty of Engineering, Baden-Württemberg Cooperative State University Karlsruhe, 76133 Karlsruhe, Germany

**Keywords:** sandwich complexes, heterocyclic ligands, lanthanide, europium, arsenic, phosphorus

## Abstract

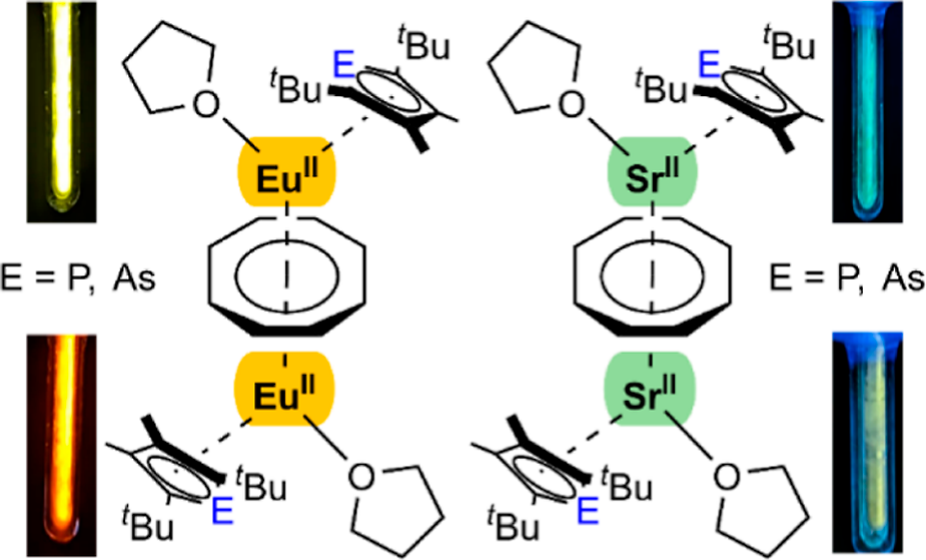

To study the influence of heteroatoms on the photophysical properties of divalent Eu and Sr complexes, the synthesis of the phospholyl and arsolyl compounds [{(Dtp)(thf)M}_2_{μ-η^8^:η^8^-C_8_H_8_}] (M = Eu^II^ and Sr^II^; Dtp = 3,4-dimethyl-2,5-bis(*tert*-butyl)phospholyl) and [{(Dtas)(thf)M}_2_{μ-η^8^:η^8^-C_8_H_8_}] (M = Eu^II^ and Sr^II^; Dtas = 3,4-dimethyl-2,5-bis(*tert*-butyl)arsolyl) is reported. Organometallic compounds of divalent europium with P and As heterocyclic ligands have not been described previously. They were prepared by salt elimination reactions from potassium phospholyl or arsolyl, K_2_C_8_H_8_, and EuI_2_(thf)_2_ or SrI_2_. Photophysical properties were investigated alongside a reference cyclopentadienyl complex with a comparable structure. Critically, the influence of the heteroatom on the photoluminescence emission and excitation and quantum yields of the complexes is significant. Density functional theory calculations were performed to rationalize the ligand influences.

## Introduction

In recent years, the exploration of heterocyclopentadienyl ligands, in which at least one carbon atom is replaced by a heteroatom, has gained significant interest in the field of lanthanide chemistry.^1−4^ Although the chemistry of phospholyl ligands with lanthanides has been extensively studied,^5,6^ it is noteworthy that no sandwich complex with divalent Eu has been reported thus far. This is surprising considering that sandwich complexes of other classical divalent lanthanides, such as Sm and Yb, have been reported.^7^ In contrast, triple-decker complexes of the divalent lanthanides featuring a heterocyclic deck are unknown. Furthermore, it is intriguing that none of these complexes have been reported, given that divalent Eu complexes often exhibit interesting photophysical properties, such as high quantum efficiency. Similarly, for strontium, which often shows a coordination behavior comparable to divalent europium, due to their similar ionic radii, only one phospholyl complex has been reported.^8^

Generally, lanthanide–arsolyl complexes are less common compared to their lighter counterparts. Currently, only for one arsolyl complex of a divalent lanthanide, [(Dsas)_2_Tm^II^(thf)] (Dsas = 3,4-dimethyl-2,5-bis(trimethylsilyl)arsolyl),^9^ the solid-state structure has been established by single-crystal X-ray diffraction, while the other two examples were solely characterized by NMR spectroscopy.^7^ This trend extends to the alkaline-earth metals, where only two examples of a calcium–arsolyl complex have been described in the existing literature.^10,11^

Multidecker complexes featuring divalent lanthanides have been the subject of extensive research. In 1995, Evans and co-workers discovered the first instance of a triple-decker complex with a COT (COT = C_8_H_8_^2–^) ligand. By reacting EuCl_3_ and K_2_COT with KCp* (Cp* = C_5_Me_5_^–^), they obtained the divalent triple-decker complex [{(Cp*)(thf)_2_Eu^II^}_2_{μ-η^8^:η^8^-C_8_H_8_}],^12^ which exhibited a bent structure that remained intact even after the removal of coordinated THF through high vacuum heating.^13^ Similar behavior was observed for the analogous Yb and Sm compounds in other reports by Evans and co-workers.^14^ Recently, our group has also succeeded in synthesizing cyclic sandwich complexes (cyclocenes), with the general formula [*cyclo*-M^II^(μ-η:^8^η^8^-COT^TIPS^)]_18_ (M = Sr^II^, Sm^II^, and Eu^II^, COT^TIPS^ = 1,4-(*^i^*Pr_3_Si)_2_C_8_H_6_^2−^).^15^

Our curiosity was sparked by the scarcity of divalent europium and alkaline-earth complexes involving heterocyclopentadienyl ligands, as well as the limited exploration of europium chemistry within the extensively studied divalent triple-decker complex class. In fact, no divalent lanthanide triple-decker complex featuring a heterocyclic ligand in the coordination sphere is known. Motivated by this, we sought to investigate the influence of different ligand systems and especially the heteroatoms on COT-bridged triple-decker lanthanide and alkaline-earth complexes. Specifically, we aimed at generating europium and strontium triple-decker compounds incorporating phospholyl and arsolyl ligands,^1^ with a particular focus on studying their photophysical properties, since the luminescence of divalent europium adds further incentive to explore its chemistry.

Herein, the syntheses and structures of the triple-decker complexes [{(Dtp)(thf)M}_2_{μ-η^8^:η^8^-C_8_H_8_}] (M = Eu^II^ and Sr^II^; Dtp = 3,4-dimethyl-2,5-bis(*tert*-butyl)phospholyl) and [{(Dtas)(thf)M}_2_{μ-η^8^:η^8^-C_8_H_8_}] [M = Eu^II^ and Sr^II^, Dtas = 3,4-dimethyl-2,5-bis(*tert*-butyl)arsolyl] are reported. These compounds were fully characterized and the photophysical properties of the synthesized complexes as well as one example from the literature were investigated by measuring UV–vis, photoluminescence emission (PL) and excitation (PLE) spectra. The luminescence properties of the Eu complexes were also compared to the previously reported complex [{(Cp*)(thf)_2_Eu^II^}_2_{μ-η^8^:η^8^-C_8_H_8_}].^12^

## Results and Discussion

### Synthesis and Characterization

**Scheme 1:**
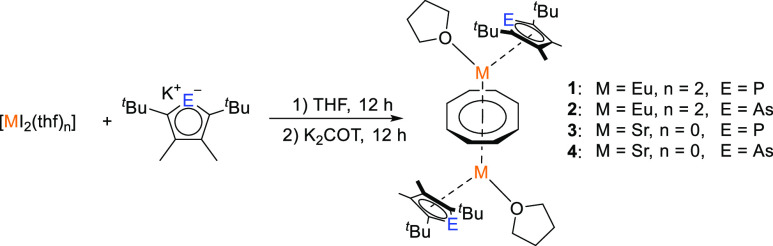
Synthesis of the Complexes [{(η^5^-Dtp)Eu(thf)}_2_{μ-η^8^:η^8^-C_8_H_8_}] (**1**), [{(η^5^-Dtas)Eu(thf)}_2_{μ-η^8^:η^8^-C_8_H_8_}] (**2**), [{(η^5^-Dtp)Sr(thf)}_2_{μ-η^8^:η^8^-C_8_H_8_}] (**3**), and [{(η^5^-Dtas)Sr(thf)}_2_{μ-η^8^:η^8^-C_8_H_8_}] (**4**)

For the synthesis of triple-decker complexes coordinated by phospholyl and arsolyl ligands, we employed a synthesis protocol based on the previously synthesized classical cyclopentadienyl complex [{(Cp*)(thf)Yb^II^}_2_{μ-η^8^:η^8^-C_8_H_8_}] as a reference.^13^ In a two-step protocol, equimolar amounts of [Eu^II^I_2_(thf)_2_] were reacted with the respective potassium phospholyl [K(Dtp)] or arsolyl [K(Dtas)] complexes in THF (Scheme 1). After 12 h, 0.5 equiv of K_2_COT was added in a second step. As the reaction progressed, a noticeable transformation occurred. The intense blue luminescence of [Eu^II^I_2_(thf)_2_] gradually diminished and was replaced by a distinct, intense orange-yellow luminescence, clearly visible under UV excitation (365 nm) at room temperature. The resulting mixtures were then filtered and compounds [{(η^5^-Dtp)Eu^II^(thf)}_2_{μ-η^8^:η^8^-C_8_H_8_}] (**1**) and [{(η^5^-Dtas)Eu^II^(thf)}_2_{μ-η^8^:η^8^-C_8_H_8_}] (**2**) were obtained as single crystals through slow evaporation of the solvent. Both compounds crystallize in the monoclinic space group *P*2_1_/*c*, with one molecule in the asymmetric unit.

**Figure 1:**
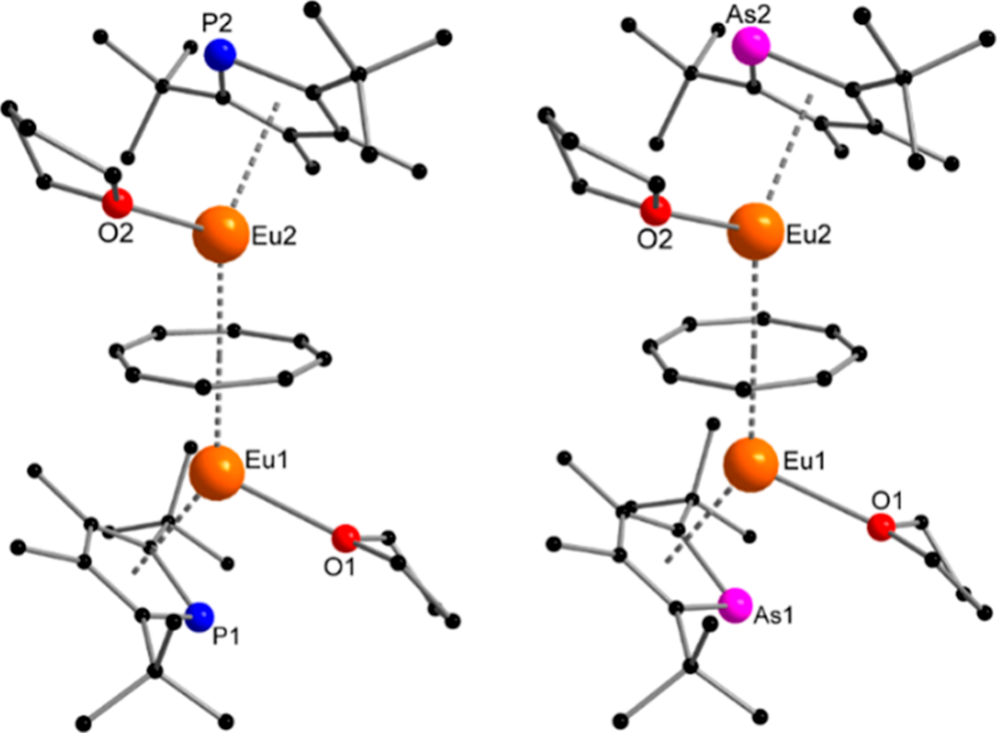
Molecular structure of **1** (left) and **2** (right) in the solid state. Hydrogen atoms are omitted for clarity. Selected bond lengths [Å] and angles [°] for **1**: Eu1–P1 3.063(2), Eu2–P2 3.082(2), Eu1–Ct_Dtp1_ 2.625(3), Eu2–Ct_Dtp2_ 2.633(3), Eu1–Ct_COT_ 2.169(3), Eu1–C_COT_ 2.813(8)–2.906(8), Eu2–C_COT_ 2.801(8)–2.903(7), Eu2–Ct_COT_ 2.172(3), Eu1–O1 2.575(5), Eu2–O2 2.566(5); Eu1–Ct_COT_–Eu2 177.5(2), Ct_COT_–Eu1–Ct_Dtp1_ 144.19(2), Ct_COT_–Eu2–Ct_Dtp2_ 143.75(12). **2**: Eu1–As1 3.1395(5), Eu2–As2 3.1621(5), Eu1–Ct_Dtas1_ 2.645(2); Eu2–Ct_Dtas2_ 2.649(2), Eu1–Ct_COT_ 2.168(2), Eu1–C_COT_ 2.807(5)–2.916(5), Eu2–C_COT_ 2.822(5)–2.901(5), Eu2–Ct_COT_ 2.175(2), Eu1–O1 2.577(3), Eu2–O2 2.571(3); Eu1–Ct_COT_–Eu2 177.23(11), Ct_COT_–Eu1–Ct_Dtas1_ 144.51(6), Ct_COT_–Eu2–Ct_Dtas2_ 143.88(7).

The overall structure of the complexes aligned with our expectations, with the COT ligand positioned as a bridging deck between the two europium atoms and the phospholyl and arsolyl ligands being η^5^-coordinated on the top and bottom of the molecule (Figure 1). Notably, to each europium atom, an additional THF molecule is bound. In comparison, in the analogous [{(Cp*)Eu^II^(thf)_2_}_2_{μ-η^8^:η^8^-C_8_H_8_}], two THF molecules are attached to each europium atom.^12^ This indicates the increased steric demand of the phospholyl and arsolyl ligands compared to the Cp* ligand. The two phospholyl and arsolyl ligands are located in a trans arrangement relative to each other, with the coordinating THF molecules also being on opposite sides of the molecule. The [Eu^II^_2_(μ-η^8^:η^8^-C_8_H_8_)]^2+^ fragments of compounds **1** and **2** exhibit nearly linear geometries, with Eu1–Ct_COT_–Eu2 (Ct = centroid) angles of 177.5(2) and 177.23(11)°, respectively.

In compound **1**, the Eu1–C_COT_ distances range from 2.813(8) to 2.906(8) Å, while the Eu1–Ct_COT_ distance measures 2.169(3) Å. Similarly, the Eu2–C_COT_ distances range from 2.801(8) to 2.903(7) Å with the Eu2–Ct_COT_ distance being 2.172(3) Å. As expected, complex **2** displays almost identical bond lengths to the COT ligand, due to being isostructural to **1**. The main differences between the two compounds arise from the variation in the heteroatom within the η^5^-coordinated five-membered ring. While the Eu1–P1 and Eu2–P2 distance in **1** is 3.063(2) and 3.082 (2) Å, the Eu1–As1 and Eu2–As2 bond lengths are 3.1395(5) and 3.1621(5) Å, respectively.

This is the result of the larger ionic radius of arsenic compared to phosphorus.^16^ The Eu–P bond lengths correspond well to previously reported Sm–Dtp complexes (since no Eu–phospholyl complexes are known, the slightly larger divalent samarium ion was used as a reference).^17^ The influence of the larger arsenic atom is also reflected in the distances between the europium atom and the centroids of the phospholyl and arsolyl ligands. The Eu1–Ct_Dtp1_ and Eu2–Ct_Dtp2_ distances are 2.625(3) and 2.633(3) Å, whereas Eu1–Ct_Dtas1_ and Eu2–Ct_Dtas2_ are slightly elongated with 2.645(2) and 2.649(2) Å. Another feature of these structures is their bent conformation, typical for such lanthanide triple-decker systems,^13,14^ as can be seen by the Ct_COT_–Eu–Ct_Dtp_ angles of 144.19(2) and 143.75(12)° and the Ct_COT_–Eu–Ct_Dtas_ angles of 144.51(6) and 143.88(7). Attempts to desolvate the products to obtain the solvent-free triple-decker complexes by heating the complexes under vacuum (120 °C and 10^–3^ mbar) were unsuccessful.

**Figure 2:**
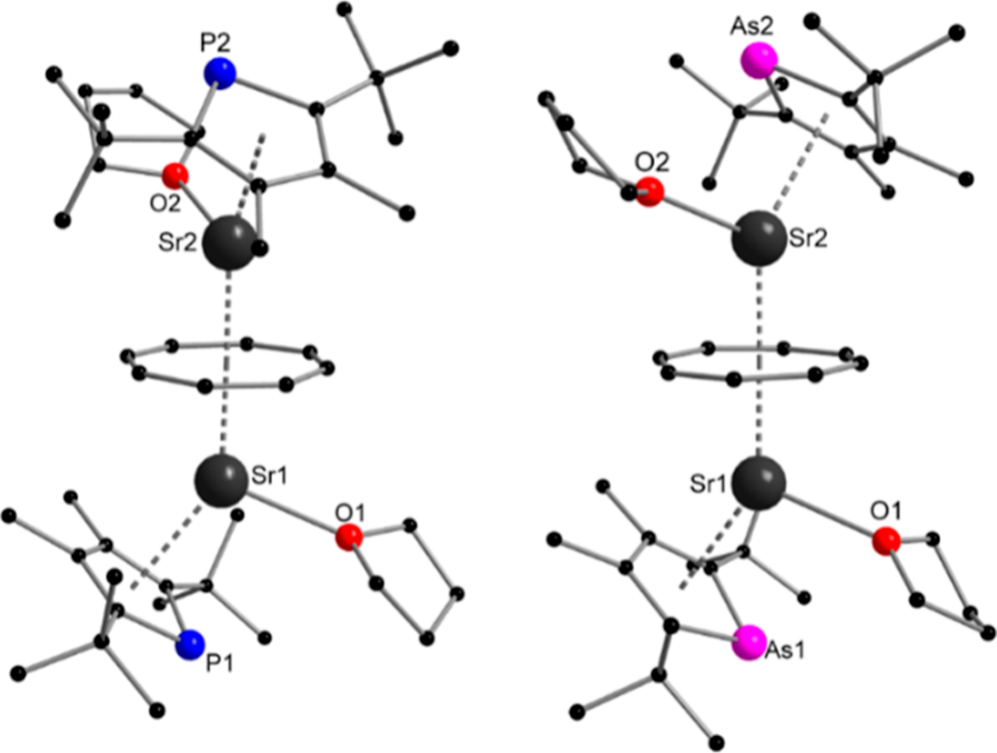
Molecular structure of **3** (left) and **4** (right) in the solid state. Hydrogen atoms are omitted for clarity. Selected bond lengths [Å] and angles [°] for **3**: Sr1–P1 3.108(3), Sr2–P2 3.085(3), Sr1–Ct_Dtp1_ 2.659(4); Sr2–Ct_Dtp2_ 2.660(4), Sr1–Ct_COT_ 2.196(4), Sr1–C_COT_ 2.825(9)–2.915(10), Sr2–C_COT_ 2.832(10)–2.916(11), Sr2–Ct_COT_ 2.191(4), Sr1–O1 2.558(7), Sr2–O2 2.545(7); Sr1–Ct_COT_–Sr2 178.1(2), Ct_COT_–Sr1–Ct_Dtp1_ 143.0(2), Ct_COT_–Sr2–Ct_Dtp2_ 143.88(13). **4**: Sr1–As1 3.1831(7), Sr2–As2 3.1647(7), Sr1–Ct_Dtas1_ 2.674(2); Sr2–Ct_Dtas2_ 2.669(2), Sr1–Ct_COT_ 2.198(2), Sr1–C_COT_ 2.831(5)–2.906(5), Sr2–C_COT_ 2.815(5)–2.914(5), Sr2–Ct_COT_ 2.189(2), Sr1–O1 2.550(4), Sr2–O2 2.556(4); Sr1–Ct_COT_–Sr2 177.77(11), Ct_COT_–Sr1–Ct_Dtas1_ 143.40(8), Ct_COT_–Sr2–Ct_Dtas2_ 144.03(7).

Due to the similarities in ionic radii and general coordination behavior of strontium and divalent europium,^18,19^ we also attempted to synthesize the analogous Sr complexes. This successfully resulted in compounds [{(η^5^-Dtp)Sr^II^(thf)}_2_{μ-η^8^:η^8^-C_8_H_8_}] (**3**) and [{(η^5^-Dtas)Sr^II^(thf)}_2_{μ-η^8^:η^8^-C_8_H_8_}] (**4**) as colorless single crystals after slow evaporation of the solvent (Scheme 1). As expected, these compounds are isostructural to the europium complexes **1** and **2**. Again, the phospholyl and arsolyl ligands are η^5^-coordinated (Figure 2). They are located at the opposite ends of the triple-decker in trans position to each other. The Sr1–P1 and Sr2–P2 bonds in **3** are 3.108(3) and 3.085(3) Å, respectively, while the Sr1–As1 and Sr2–As2 bonds in **4** exhibit lengths of 3.1831(7) and 3.1647(7) Å. These bond lengths align with the observed trend in the europium complexes **1** and **2**. It is worth noting that the Sr–P bond length observed in **3** is consistent with the only previously reported Sr–phospholyl complex documented in the literature.^8^ A corresponding arsolyl complex is not known. The Sr–Ct_Dtas_ bonds in **4** are quite similar in length to the Sr–Ct_Dtp_ bonds in **3**. Also, the Sr–Ct_COT_ bonds in both complexes are almost identical. Both compounds show almost linear coordination of the COT ligand with angles of Sr1–Ct_COT_–Sr2 of 178.1(2)° for **3** and 177.77(11)° for **4**. The change of the heterocyclic ligand does also not significantly influence the Ct_COT_–Sr–Ct_Dtp_ and Ct_COT_–Sr–Ct_Dtas_ angles, with them being very similar to each other.

Since the obtained Sr complexes **3** and **4** are diamagnetic, contrary to the paramagnetic Eu complexes **1** and **2**, they could be unambiguously characterized by ^1^H, ^13^C{^1^H}, and ^31^P{^1^H} (for **3**) NMR spectroscopy. The NMR spectra of both compounds (Figures S1–S5, Supporting Information) show no major differences. Therefore, only the ones of **3** are discussed in detail. The signal of the COT protons appears at 6.10 ppm, while the signals of the methyl groups and the ^*t*^Bu groups are observed at 2.22 and 1.78 ppm, respectively. The protons of the coordinating THF molecules are also visible. In the ^13^C{^1^H} spectrum, a slight difference of **3** and **4** is seen, as all carbon signals from the arsolyl ligand are shifted to higher frequencies compared to the phospholyl ligand, probably because of electronic effects in the ring due to the heavier arsenic atom. In the ^31^P{^1^H} NMR spectrum, one peak at 71.2 ppm is observed, indicative of a η^5^-coordinated phospholyl ligand being equivalent in solution.^20,21^

### Photophysical Measurements

**Figure 3:**
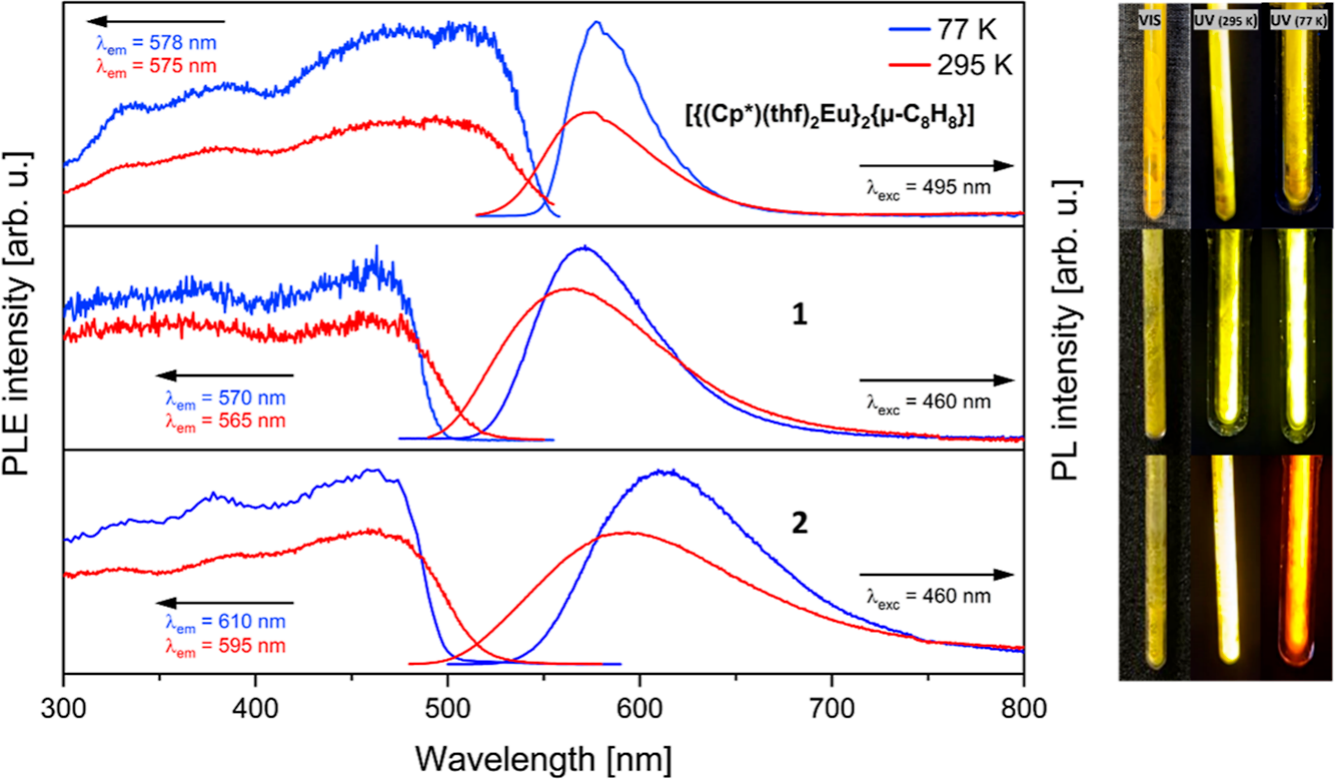
Solid-state PL and PLE spectra of complexes [{(Cp*)(thf)_2_Eu^II^}_2_{μ-η^8^:η^8^-C_8_H_8_}] (top), **1** (middle), and **2** (below) at 77 K (blue lines) and 295 K (red lines). The PL emission was excited at 495 nm for [{(Cp*)(thf)_2_Eu}_2_{μ-C_8_H_8_}] and at 460 nm for **1** and **2** and the PLE spectra were recorded at the indicated wavelengths. The pictures show the samples [{(Cp*)(thf)_2_Eu}_2_{μ-C_8_H_8_}] (top), **1** (middle), and **2** (bottom) under daylight (left) and UV lamp illumination (365 nm) at 295 K (middle) and 77 K (right).

Due to the Laporte rule, f–f-transitions in trivalent lanthanide complexes are parity-forbidden. Additionally, the shielded f-orbitals of trivalent lanthanides render influences of the ligand sphere on luminescence properties almost negligible. On the other hand, in divalent lanthanides, 4f → 5d processes are commonly observed. These parity-allowed transitions lead to broader bands and intense emissions. Since fluorescence is a known characteristic of divalent europium compounds,^22^ we observed bright emission from complexes **1** and **2** at room temperature under UV excitation (365 nm). To investigate this further, we recorded PL and PLE spectra in the solid state at both 295 and 77 K, as depicted in Figure 3.

The PLE spectra of the europium complexes, at both temperatures, exhibit a broad, unstructured absorption band, typical for divalent europium,^23−25^ which can be attributed to parity-allowed 4f^7^ → 4f^6^5d^1^ transitions (Figure 3).

For the phospholyl-substituted complex **1**, the PL spectra excited at λ_exc_ = 460 nm display a distinct, slightly asymmetric peak at λ_em_ = 570 nm (77 K) and λ_em_ = 565 nm (295 K), respectively, arising from the 4f^6^5d^1^ → 4f^7^ relaxation process. Notably, a slight red shift in the emission maximum is observed upon cooling. Furthermore, we observed an increase in emission intensity at 77 K, indicating the suppression of thermally activated nonradiative processes at lower temperatures. A sharpening of the emission bands is also visible upon cooling, when comparing the full widths at half-maximum (fwhms) with 100 nm at 295 K and 72 nm at 77 K. The excitation spectra of complex **2** exhibit a broad and unstructured excitation band similar to that observed in complex **1**. This observation suggests that the same 4f^7^ → 4f^6^5d^1^ transitions occur in both complexes. Again, the PL spectra of **2** show one emission maximum at λ_em_ = 610 nm (77 K) and λ_em_ = 595 nm (295 K), when being excited at λ_exc_ = 460 nm (Figure 3). At lower temperatures, the emission shows a bathochromic shift of 15 nm, also evident to the naked eye as the luminescence under UV excitation (365 nm) changes from yellow at room temperature to orange at 77 K. Again, a sharpening of the peak can be observed upon cooling with a fwhm of 131 nm at 295 K and 103 nm at 77 K.

While the change from phosphorus to arsenic in the ligand does not have a significant influence on the structure of the complex (see above), the luminescence properties are strongly dependent on the ligand system coordinated to the divalent europium resulting in a shift of the respective emission maxima. Upon changing the phospholyl to an arsolyl ligand, the emission of **1** is shifted from λ_em_ = 565 nm to λ_em_ = 595 nm at 295 K in **2**. A similar bathochromic shift can be seen at 77 K as well, with the phospholyl-ligated complex emitting at λ_em_ = 570 nm, while the arsolyl compound emits at λ_em_ = 610 nm. Furthermore, we conducted emission lifetime measurements for both complexes. For **1**, this resulted in a total value of 2.49 μs at 77 K. At higher temperatures (295 K), the emission lifetime decreased slightly to 1.48 μs. In contrast, the arsolyl-coordinated compound **2** exhibited slightly shorter fluorescence lifetimes.

At lower temperatures, the lifetime of **2** was determined to be 1.58 μs, while at higher temperatures, it decreased to 1.25 μs. The observed lifetimes are consistent with those of previously documented divalent europium compounds.^26−28^ However, it is noteworthy that there are also compounds characterized by longer lifetimes, reaching up to 50 μs,^28^ as well as others exhibiting shorter lifetimes in the nanosecond range.^29^ Furthermore, as recently shown, the substituents on the rings also play a crucial role in luminescence lifetimes.^30^ Quantum yield measurements were conducted for both europium complexes in the solid state at room temperature. The quantum yield of **1** was found to be 73%, while for **2**, it was 69%.

To investigate the influence of the heteroatoms on the luminescence of these triple-decker structures, we also measured the PLE and PL spectra of the complex [{(Cp*)(thf)_2_Eu^II^}_2_{μ-η^8^:η^8^-C_8_H_8_}],^12^ which has been reported previously but not characterized in terms of its photophysical properties. This compound exhibits a broad band in the excitation spectrum, similar to **1** and **2**, typical for divalent europium. This band extends up to around 550 nm, after which the PLE intensity starts to decline. In contrast, for **1** and **2**, the excitation onset is around 500 nm. The emission upon excitation at λ_exc_ = 495 nm is characterized by peaks at λ_em_ = 578 nm (77 K) and λ_em_ = 575 nm (295 K) with a fwhm of 46 and 65 nm, respectively, being consistent with the yellow color of the observed emission. The emission wavelengths are close to the phospholyl-coordinated compound **1** (570 nm at 77 K and 565 nm at 295 K), while the emission bands are sharper at both temperatures. The results indicate that substituting the ligand from Cp* to phospholyl has only a small impact on the luminescence properties of these compounds. However, when changing from Cp* to the arsolyl ligand, a noticeable red shift in the PL spectra is observed. Apart from the shift in emission maxima, one of the main differences is the width of the emission bands. The Cp*-coordinated complex exhibits relatively sharp bands for a divalent europium complex, whereas both **1** and **2** have significantly broader bands. Upon analyzing the lifetimes of the previously reported Cp* complex [{(Cp*)(thf)_2_Eu^II^}{μ-η^8^:η^8^-C_8_H_8_}], it was determined that, at room temperature, the complex exhibited a fluorescence lifetime of 1.34 μs. This lifetime increased to 1.84 μs at 77 K, placing it within the range observed for complexes **1** and **2**. Furthermore, we also conducted quantum yield measurements for [{(Cp*)(thf)_2_Eu^II^}_2_{μ-η^8^:η^8^-C_8_H_8_}]. The obtained quantum yield of 40% stands out, albeit for its relatively lower value compared to that of the phospholyl- and arsolyl-coordinated compounds. This disparity might arise from the distinctive coordination environment, where each Eu atom binds two THF molecules, in contrast to the coordination of one THF molecule observed in **1** and **2**.

Additionally, PL and PLE spectra of **1** and **2** were measured in THF (see Supporting Information Figures S16 and S20). However, no significant differences were observed compared to the previous measurements in the solid state.

**Figure 4:**
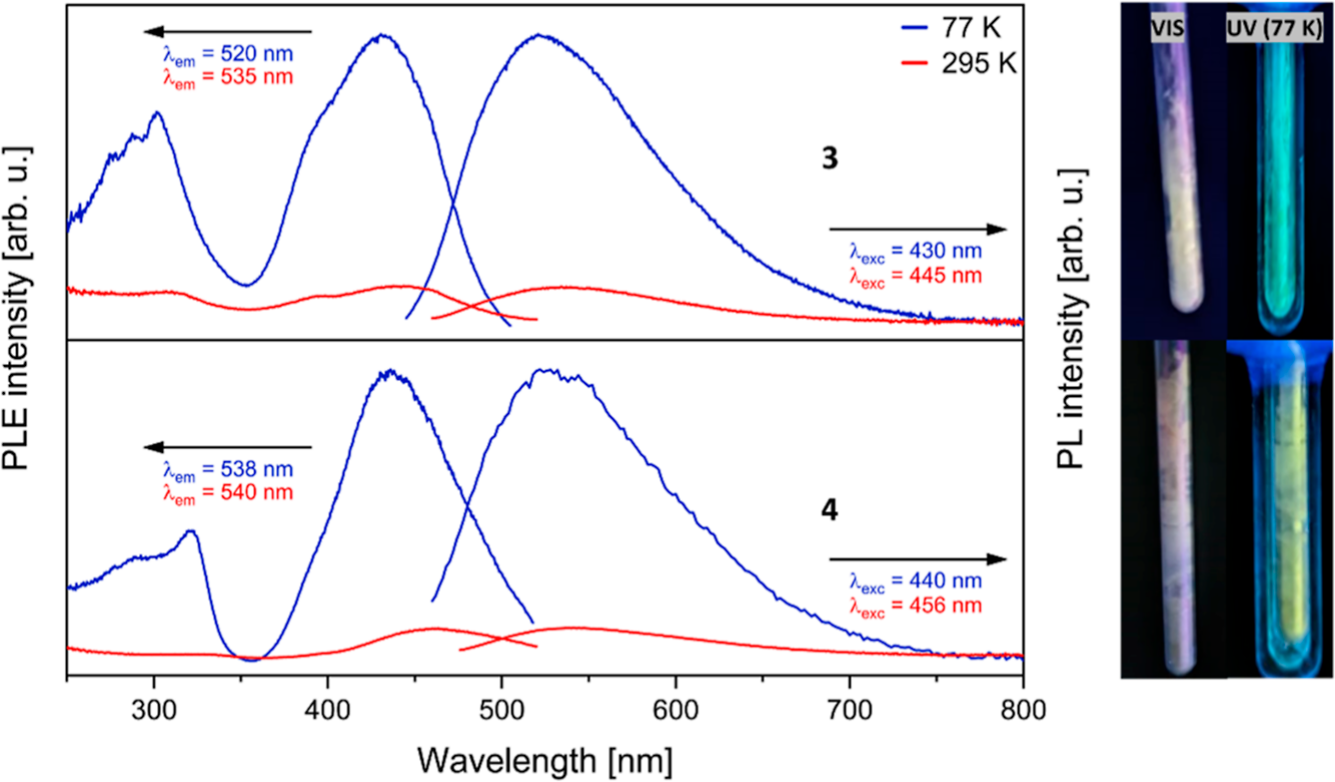
Solid-state PL and PLE spectra of complexes **3** (top) and **4** (below) at 77 K (blue lines) and 295 K (red lines). The PL emission was in each case excited at 460 nm and the PLE spectra were recorded at the indicated wavelengths. The pictures show the samples under daylight (left) and UV lamp illumination (365 nm) at 295 K (middle) and 77 K.

The luminescence observed for the europium complexes is readily apparent, whereas for the strontium complexes, no luminescence is noticeable upon excitation with a UV lamp (365 nm) at room temperature. However, when the solid complexes **3** and **4** are cooled to 77 K and excited with UV light, a weak emission is observable. This is interesting, since Sr possesses a closed-shell electron configuration, suggesting that the heterocyclic ligands are responsible for the luminescence, as the starting materials K(Dtp) and K(Dtas) also show emission after UV excitation at lower temperatures (see Figures S34 and S35). Therefore, we also measured the PLE and PL spectra of these strontium complexes (Figure 4).

The excitation spectrum of compound **3** at 77 K is characterized by two bands at 305 and 430 nm. Likewise, compound **4** exhibits similar excitation spectra at 77 K, although the peaks are shifted to slightly higher wavelengths (322 and 440 nm).

Compound **3** exhibits broad emission peaks at λ_em_ = 520 nm (77 K) and at λ_em_ = 535 nm (295 K). The PL spectra indicate a significant reduction in emission intensity at higher temperatures compared to lower temperatures. Similarly, the emission of **4** is much stronger at 77 K than at 295 K, with the emission maximum only slightly shifting from λ_em_ = 538 nm (77 K) to λ_em_ = 540 nm (295 K). The Stokes shift for compound **3** remains constant with 0.5 eV for both temperatures, whereas for compound **4**, it shows a variation from 0.5 eV at 77 K to 0.4 eV at 295 K. The lifetime of these fluorescence processes could not be determined reliably, since the duration of these processes is around 2 ns, which also represents the lower limit of the detector. Due to the weak emission only visible at 77 K, no quantum yields could be determined for these complexes. Additionally, the luminescence spectra of **3** and **4** in THF were measured at 77 K (Figures S27 and S31), since only very weak emissions were observed at room temperature. Unlike in the solid state, only one peak was observed in the excitation spectra (λ_exc_ = 315 nm for **3** and λ_exc_ = 334 nm for **4**) in THF solution. The emission wavelengths of **3** (λ_em_ = 495 nm) and **4** (λ_em_ = 505 nm) exhibited a significant blue shift compared to the previous solid-state measurements. Moreover, in contrast to the solid-state measurements, fluorescence and phosphorescence processes with extended lifetimes were observed (see Figures S28 and S32).

### Quantum Chemical Calculations

The experimental work was accompanied by theoretical studies to evaluate the influence of phospholyl and arsolyl ligands on the photophysical properties of the europium compounds. We used the TURBOMOLE^31,32^ program package to investigate the electronic excitations and fluorescence of the europium compounds **1**, **2**, and [{(Cp*)(thf)_2_Eu^II^}_2_{μ-η^8^:η^8^-C_8_H_8_}], as well as of the strontium compounds **3** and **4**. Structure parameters of the ground states as well as in part of the first excited singlet states (**3** and **4**) have been optimized at the density functional theory (DFT) level, employing the PBE0 hybrid functional^33,34^ and def2-TZVP basis sets for all atoms.^35^

The structural results of the ground-state molecules are in excellent agreement with the experimentally deduced values (distances **1**: Eu–P 3.040 Å, Eu–Ct_Dtp_ 2.624 Å, Eu–Ct_COT_ 2.214 Å; **2**: Eu–As 3.138 Å, Eu–Ct_Dtp_ 2.644 Å, Eu–Ct_COT_ 2.221 Å; **3**: Sr–P 3.072 Å, Sr–Ct_Dtp_ 2.647 Å, Sr–Ct_COT_ 2.244 Å; **4**: Sr–As 3.169 Å, Sr–Ct_Dtp_ 2.668 Å, Sr–Ct_COT_ 2.246 Å). As we expected a strong influence of the europium f electrons on the electronic situation, we initially investigated the two strontium compounds **3** and **4** for simplicity. The lowest singlet vertical excitations, corresponding to the UV–vis absorptions, were calculated using time-dependent DFT (TD-DFT) to be 306 nm (**3**) and 311 nm (**4**), respectively, showing the same trend as the experimental PLE values of 430 nm (**3**) and 440 nm (**4**) (see MO diagrams in Figure S39). The associated fluorescence transitions were determined to be at 506 nm (**3**) and 608 nm (**4**), respectively, obtained after geometry optimization of the first excited singlet state by TD-DFT. The values are in good accord to the measured emissions [520 nm (**3**) and 538 nm (**4**)]. Excitation of **3** or **4** mainly increases the Sr–P and Sr–As distance, respectively (distances: Sr1–E1/Sr2–E2 (E = P and As) **3**: Sr–P 3.076/3.138 Å, Sr–Ct_Dtp_ 2.652/2.649 Å, Sr–Ct_COT_ 2.235/2.273 Å; **4**: Sr–As 3.172/3.225 Å, Sr–Ct_Dtas_ 2.673/2.645 Å, Sr–Ct_COT_ 2.235/2.275 Å). The excitations can be attributed to HOMO–LUMO transitions (π-type character) centered at the phospholyl or arsolyl ligands. The small differences can be rationalized by the differing involvement of the heteroatoms on the electronic transitions. The nonrelaxed difference electron density plots upon fluorescence are presented in Figure S38 of the Supporting Information.

Because of the complicated electronic situation caused by the 14 unpaired f electrons in **1** and **2**, we did not perform excited-state geometry optimizations and thus did not consider the emission process which is already described in detail above. Instead, we investigated the excitations from the ground-state geometry using TD-DFT. These results are interpreted in a more qualitative way. As expected, the excitations of the two europium compounds **1** and **2** are dominated by 4f^6^5d^1^ ← 4f^7^ transitions.

**Figure 5:**
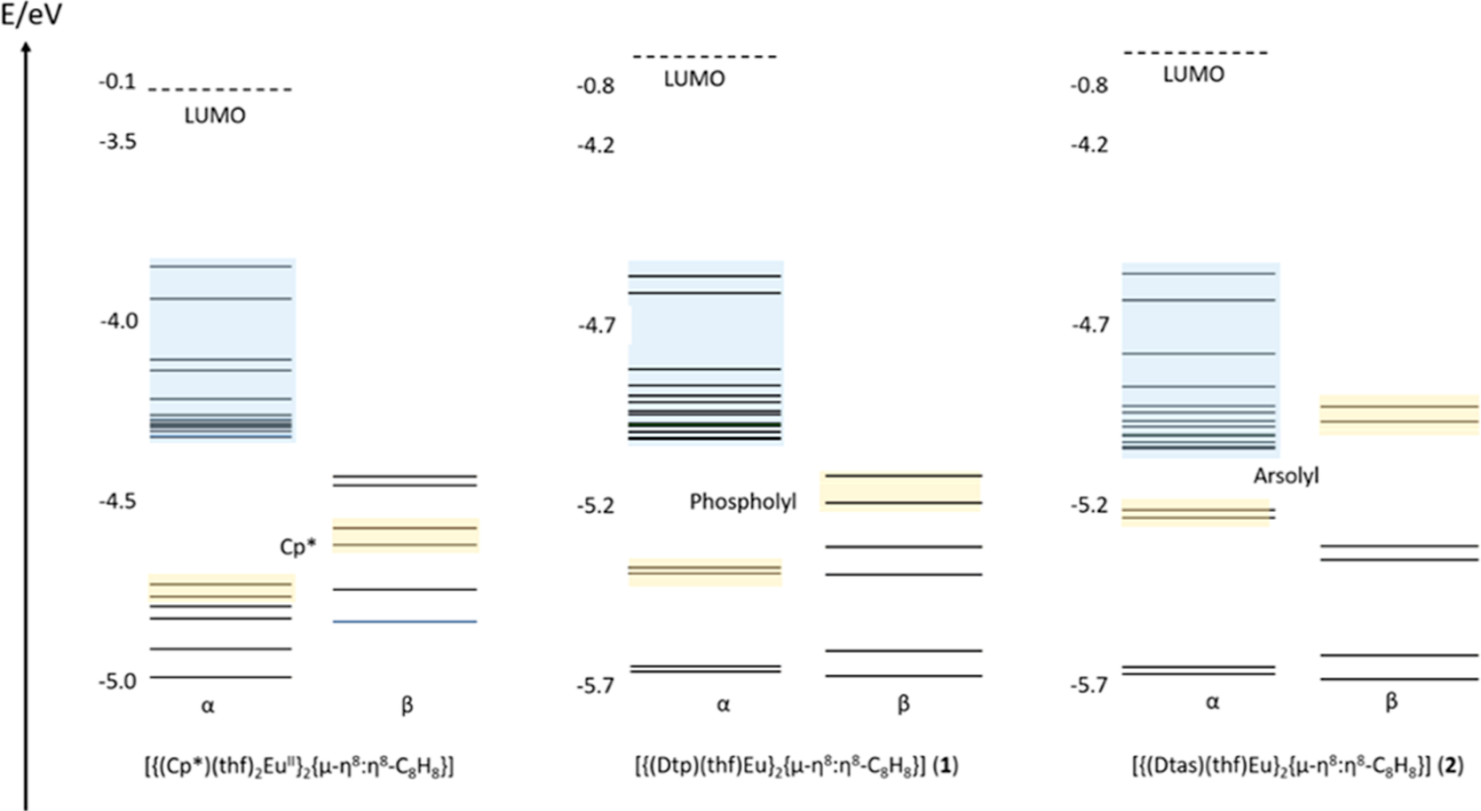
MO diagrams of the ground states of **1**, **2**, and [{(Cp*)(thf)_2_Eu}_2_{μ-η^8^:η^8^-C_8_H_8_}] (RI-DFT, PBE0, def2-TZVP). The energetically highest π-MOs of the Cp*, phospholyl, and arsolyl ligands are depicted in light yellow; the 14 f (α-)electrons of the two europium atoms are given in light blue. *Y* axes refer to energies in eV.

Deeper insights on the influence of the phospholyl and arsolyl ligands on the fluorescence of the europium compounds can be obtained from the MO diagrams of **1**, **2**, and [{(Cp*)(thf)_2_Eu^II^}_2_{μ-η^8^:η^8^-C_8_H_8_}] (Figure 5). The highest occupied π-MOs of the cyclopentadienyl, phospholyl, and arsolyl ligands with β-spin do not interact with the α-spin AOs of Eu^2+^. However, the MO diagram gives insights into the fundamentally different energy situation of the ligand MOs with the Eu f electrons: mixing of the Eu f electrons with the α-MOs of the arsolyl ligand is expected to be rather strong, whereas that with the phospholyl ligand is less pronounced. Isosurface plots of those highest ligand π-MOs with α-spin of the three compounds under discussion are presented in Figure S40 of the Supporting Information. Taking into account the results of Mulliken population analysis of these MOs ([{(Cp*)(thf)_2_Eu^II^}_2_{μ-η^8^:η^8^-C_8_H_8_}]: q(Eu f) 0.66, **1**: 0.62; **2**: 0.99), one finds that Eu f contribution of the cyclopentadienyl and the phospholyl species is of comparable size but increases significantly to the arsolyl species. This confirms the situation depicted in Figure 5. To evaluate the situation upon excitation in a qualitative way, the nonrelaxed difference electron density plots of the first two (almost) degenerate electronic transitions of the absorptions in **1** and **2** were determined (Figure S38). One finds the dominance of the 4f^6^5d^1^ ← 4f^7^ transitions influenced by the arsolyl or less pronounced by the phospholyl ligands but in a certain degree also by the COT ligand.

## Conclusions

In summary, a comprehensive study of the influence of heteroatoms in heterocyclic Eu and Sr triple-decker complexes is presented. For this purpose, we have reported the synthesis and structural characterization of COT-bridged Eu^II^ and Sr^II^ triple-decker complexes **1–4**, coordinated by phospholyl and arsolyl ligands. Remarkably, these complexes represent the first organometallic compounds of europium with such ligands, while also introducing the first example of a strontium arsolyl complex to date. The title compounds are also the first divalent rare-earth triple-decker complexes featuring a heterocyclic deck. Comparisons showed minimal impact on the structural properties of the complexes upon changing the heteroatom in the ligand. In contrast, a strong influence of the heteroatom is seen for the photophysical properties. The influence of the heteroatom is strong in the arsolyl and less pronounced in the phospholyl case. For all complexes, PLE and PL spectra were measured and compared to the all-carbon analogue [{(Cp*)(thf)_2_Eu^II^}_2_{μ-η^8^:η^8^-C_8_H_8_}]. The data revealed that the change from P to As in the ligand system results in a red shift of the emission peak at both 77 and 295 K. Notably, the quantum yield measurements demonstrated enhanced values for **1** and **2** compared to the Cp*-coordinated compound due to slight structural differences. The influence of the cyclopentadienyl, phospholyl, and arsolyl ligands on the electronic situation has also been investigated by theoretical calculations, which support the experimental observations.

## Methods

### Materials and Reagents

All air- and moisture-sensitive manipulations were performed under a dry N_2_ or Ar atmosphere using standard Schlenk techniques or in an argon-filled MBraun glovebox, unless otherwise stated. Solvents were dried using an MBraun solvent purification system (SPS-800) and degassed. THF was additionally distilled under nitrogen from potassium benzophenone ketyl before storage over a 4 Å molecular sieve. THF-*d*_8_ was dried over a Na–K alloy. All deuterated solvents were degassed by freeze–pump–thaw cycles. The starting materials [EuI_2_(thf)_2_],^36^ K_2_COT,^37^ K(Dtp),^17^ K(Dtas),^38^ and [{(Cp*)(thf)_2_Eu}_2_{μ-C_8_H_8_}]^12^ were prepared according to literature known procedures. SrI_2_ was obtained from commercial sources and used without further purification.

### General Procedure for the Synthesis of the Complexes

The synthesis of **1** is given as an example. At −78 °C, 10 mL of THF was condensed onto a mixture of K(dtp) (71.6 mg, 0.27 mmol, 2.00 equiv) and [EuI_2_(thf)_2_] (150 mg, 0.27 mmol, 2.00 equiv). The suspension was warmed to room temperature and stirred for 12 h. After that, K_2_COT (24.9 mg, 0.14 mmol, 1.00 equiv) was added and the suspension was stirred for another 12 h. The reaction mixture was then filtered over a glass frit to remove the precipitated KI. The product could be isolated by slow evaporation of the solvent in the form of bright-yellow luminescent crystals.

## Supporting Information Available

Experimental procedures and characterization details, NMR, IR, computational and structural information, and luminescence measurements, including figures depicting nonrelaxed difference electron densities of the compounds, MO diagrams, and isosurface plots and tables of MO energies of the relevant occupied and unoccupied π-MOs of ligands and calculated total energies and Cartesian coordinates of the compounds (PDF)
Crystallographic data for complex **1** (CIF)
Crystallographic data for complex **2** (CIF)
Crystallographic data for complex **3** (CIF)
Crystallographic data for complex **4** (CIF)

## Supplementary Material

au4c00300_si_001.pdf

au4c00300_si_002.cif

au4c00300_si_003.cif

au4c00300_si_004.cif

au4c00300_si_005.cif
