# Supplementary material for: Phospholyl and Arsolyl Triple-Decker Sandwich Complexes of Europium(II) and Strontium(II)
Source: JACS Au. 2024 Jun 6;4(6):2343–50. doi: 10.1021/jacsau.4c00300 (PMC11200235; doi:10.1021/jacsau.4c00300)
Supplement: Supplementary file 1 — au4c00300_si_001.pdf [file au4c00300_si_001.pdf]

SUPPORTING INFORMATION

Phospholyl and Arsolyl Triple-Decker Sandwich Complexes of Europium (II) and Strontium (II)

Noah Schwarz,[a] Julia Feye,[ab] Vanitha R. Naina,[a] Ralf Köppe,[a] Sebastian Gillhuber,[a] Xiaofei Sun,[a] Peter W. Roesky*[a]

[a] Institute of Inorganic Chemistry, Karlsruhe Institute of Technology, Kaiserstr. 12, 76131 Karlsruhe, Germany

[b] Faculty of Engineering, Baden-Württemberg Cooperative State University Karlsruhe, 76133 Karlsruhe, Germany

E-mail: roesky@kit.edu

Table of Contents

Synthesis and characterization S3
General procedures S3
Synthesis of complexes S4
NMR Spectra S6
IR spectra S9
X-ray crystallography S11
General methods S11
Summary of crystal data S12
Crystal structures S13
Photoluminescence measurements S15
UV-Vis measurements S29
Theoretical calculations S30
References S52

Synthesis and characterization

General procedures

All air- and moisture-sensitive manipulations were performed under dry N_2 or Ar atmosphere using standard Schlenk techniques or in an argon-filled MBraun glovebox, unless otherwise stated. Prior to use, CH_2Cl_2 and MeCN were dried by refluxing over P_2O_5 and CaH_2, respectively, and distilled under a nitrogen atmosphere. Other solvents (THF, Et_2O and toluene) were dried using an MBraun solvent purification system (SPS-800) and degassed. THF was additionally distilled under nitrogen from potassium benzophenone ketyl before storage over 4 Å molecular sieve. C_6D_6 was dried over Na-K alloy and $CDCl_3$ was dried over 4 Å molecular sieves. All deuterated solvents were degassed by freeze-pump-thaw cycles. The starting materials $[EuI_2(thf)_2]$[1], K_2COT[2], K(Dtp)[3], K(Dtas)[4] and $[\{(Cp^*)(thf)_2Eu\}_2\{\mu\text{-}C_8H_8\}]$[5] were prepared according to literature known procedures.

All other chemicals were obtained from commercial sources and used without further purification. NMR spectra were recorded on Bruker spectrometers (Avance III 300 MHz, Avance 400 MHz or Avance III 400 MHz). Chemical shifts are referenced using signals of the residual protio solvent (^{1}H) or the solvent (^{13}C) and are reported relative to tetramethylsilane (^{1}H, ^{13}C, ^{29}Si). All NMR spectra were measured at 298 K, unless otherwise specified. The multiplicity of the signals is indicated as s = singlet, d = doublet, t = triplet, m = multiplet and br = broad. Assignments were determined based on unambiguous chemical shifts, coupling patterns and ^{13}C-DEPT experiments or 2D correlations ($^{1}H^{1}H$ COSY, $^{1}H^{13}C$ HMQC, $^{1}H^{13}C$ HMBC). Infrared (IR) spectra were recorded in the region 3600–400 cm^{-1} on a Bruker Tensor 37 FTIR spectrometer equipped with a room temperature DLaTGS detector and a diamond attenuated total reflection (ATR) unit. Elemental analyses were carried out with an elementar Vario Micro Cube.

Synthesis of complexes

Synthesis of [{(Dtp)(thf)Eu}$_2${μ-C_8H_8}] (1)

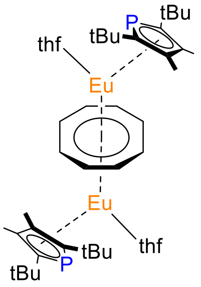

At -78 °C 10 mL of THF was condensed onto a mixture of K(dtp) (71.6 mg, 0.27 mmol, 2.00 eq.) and [EuI_2(thf)$_2$] (150 mg, 0.27 mmol, 2.00 eq.). The suspension was warmed to room temperature and stirred for 12 h. After that, K_2COT (24.9 mg, 0.14 mmol, 1.00 eq.) was added and the suspension was stirred for another 12 h. The reaction mixture was then filtered over a glass frit to remove the precipitated KI. The product could be isolated by slow evaporation of the solvent in form of bright-yellow luminescent crystals.

Crystalline yield: 53 mg, 0.05 mmol (39%).

Due to the paramagnetism of the compound, no meaningful NMR data could be obtained

IR (ATR): ($\tilde{\nu}$) [cm^{-1}] = 2955 (w), 2939 (w), 2892 (w), 2866 (w), 1846 (vw), 1730 (vw), 1604 (vw), 1457 (w), 1423 (vw), 1386 (w), 1356 (w), 1249 (w), 1198 (w), 1134 (vw), 1069 (vw), 1041 (m), 978 (w), 888 (m), 794 (vw), 768 (vw), 713 (m), 661 (vw), 610 (w), 476 (vw).

EA [%] calcd. for [$C_{44}H_{72}Eu_2O_2P_2$ - THF] (926.83 g mol^{-1}): C 51.84, H 6.96; found: C 51.39, H 7.07.

Synthesis of [{(Dtas)(thf)Eu}$_2${μ-C_8H_8}] (2)

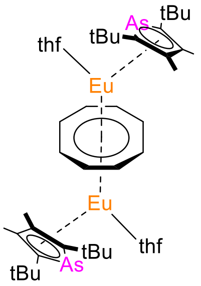

At -78 °C 10 mL of THF was condensed onto a mixture of K(dtas) (83.6 mg, 0.27 mmol, 2.00 eq.) and [EuI_2(thf)$_2$] (150 mg, 0.27 mmol, 2.00 eq.). The suspension was warmed to room temperature and stirred for 12 h. After that, K_2COT (24.9 mg, 0.14 mmol, 1.00 eq.) was added and the suspension was stirred for another 12 h. The reaction mixture was then filtered over a glass frit to remove the precipitated KI. The product could be isolated by slow evaporation of the solvent in form of yellow-orange luminescent crystals.

Crystalline yield: 72 mg, 0.06 mmol (49%).

Due to the paramagnetism of the compound, no meaningful NMR data could be obtained

IR (ATR): ($\tilde{\nu}$) [cm^{-1}] = 3006 (vw), 2942 (w), 2894 (vw), 2869 (w), 1729 (vw), 1604 (vw), 1455 (w), 1387 (vw), 1355 (w), 1303 (vw), 1241 (w), 1195 (vw), 1126 (vw), 1042 (w), 967 (vw), 888 (w), 788 (vw), 769 (vw), 713 (m), 592 (w), 424 (vw).

EA [%] calcd. for [$C_{44}H_{72}As_2Eu_2O_2$] (1086.83 g mol-1): C 48.63, H 6.68; found: C 48.78, H 6.50.

Synthesis of [{(Dtp)(thf)Sr}$_2${μ-C_8H_8}] (3)

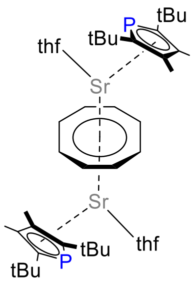

At -78 °C 10 mL of THF was condensed onto a mixture of K(dtp) (76.9 mg, 0.29 mmol, 2.00 eq.) and SrI_2 (100 mg, 0.29 mmol, 2.00 eq.). The suspension was warmed to room temperature and stirred for 12 h. After that, K_2COT (26.7 mg, 0.15 mmol, 1.00 eq.) was added and the suspension was stirred for another 12 h. The reaction mixture was then filtered over a glass frit to remove the precipitated KI. The product could be isolated by slow evaporation of the solvent in form of colourless crystals.

Crystalline yield: 62 mg, 0.07 mmol (49%).

^{1}H NMR (THF-d_8, 400 MHz): δ [ppm] = 6.10 (s, 8 H, C_8H_8), 3.62 (m, 8 H, OCH_2), 2.22 (s, 12 H, CH_3), 1.78 (m, ca. 7 H, CH_2), 1.21 (s, 36 H, C(CH_3)$_3$).

^{13}C{^{1}H} NMR (THF-d_8, 100 MHz): δ [ppm] = 154.2 (d, $^1J_{CP}$ = 47.0 Hz, C^q(C(CH$_3$)$_3$), 129.5 (C^q(CH$_3$)), 91.4 (C_8H_8)), 68.3 (OCH$_2$), 35.1 (d, $^2J_{CP}$ = 18.3 Hz, C(CH$_3$)$_3$), 33.3 (d, $^3J_{CP}$ = 11.3 Hz, C(CH$_3$)$_3$), 26.4 (CH$_2$), 17.3 (CH$_3$).

^{31}P{^{1}H} NMR (THF-d_8, 162 MHz): δ [ppm] = 71.2 (s, CPC).

IR (ATR): ($\tilde{\nu}$) [cm^{-1}] = 3009 (vw), 2956 (w), 2940 (w), 2891 (w), 2862 (w), 2165 (vw), 1982 (vw), 1724 (vw), 1599 (vw), 1458 (w), 1386 (w), 1356 (w), 1249 (w), 1197 (w), 1134 (vw), 1041 (m), 977 (vw), 888 (m), 795 (vw), 765 (vw), 712 (s), 663 (vw), 610 (w), 477 (vw).

EA [%] calcd. for [$C_{44}H_{72}O_2P_2Sr_2$ – 2 THF] (726.03 g mol^{-1}): C 59.56, H 7.77; found: C 59.66, H 7.91.

Synthesis of [{(Dtas)(thf)Sr}$_2${μ-C_8H_8}] (4)

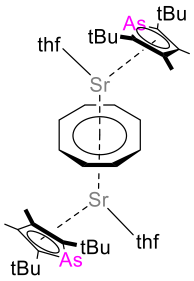

At -78 °C 10 mL of THF was condensed onto a mixture of K(dtas) (89.7 mg, 0.29 mmol, 2.00 eq.) and SrI_2 (100 mg, 0.29 mmol, 2.00 eq.). The suspension was warmed to room temperature and stirred for 12 h. After that, K_2COT (26.7 mg, 0.15 mmol, 1.00 eq.) was added and the suspension was stirred for another 12 h. The reaction mixture was then filtered over a glass frit to remove the precipitated KI. The product could be isolated by slow evaporation of the solvent in form of colourless crystals.

Crystalline yield: 63 mg, 0.07 mmol (45%).

^{1}H NMR (THF-d_8, 400 MHz): δ [ppm] = 6.10 (s, 8 H, C_8H_8), 3.62 (m, ca. 6 H, OCH_2), 2.17 (s, 12 H, CH_3), 1.78 (m, ca. 6 H, CH_2), 1.25 (s, 36 H, C(CH_3)$_3$).

^{13}C{^{1}H} NMR (THF-d_8, 100 MHz): δ [ppm] = 167.3 (C^q(C(CH$_3$)$_3$)), 130.6 (C^q(CH$_3$)), 91.4 (C_8H_8)), 68.3 (OCH$_2$), 37.0 (C(CH$_3$)$_3$), 33.5 (C(CH$_3$)$_3$), 26.4 (CH$_2$), 18.6 (CH$_3$).

IR (ATR): ($\tilde{\nu}$) [cm^{-1}] = 3010 (vw), 2943 (w), 2870 (w), 2165 (vw), 1723 (vw), 1599 (vw), 1457 (w), 1387 (vw), 1356 (w), 1303 (vw), 1241 (w), 1194 (vw), 1125 (vw), 1041 (m), 966 (vw), 888 (m), 766 (vw), 713 (m), 645 (vw), 592 (w), 499 (vw), 424 (vw).

EA [%] calcd. for [$C_{44}H_{72}As_2O_2Sr_2$] (958.14 g mol^{-1}): C 55.16, H 7.57; found: C 54.93, H 7.52.

NMR Spectra

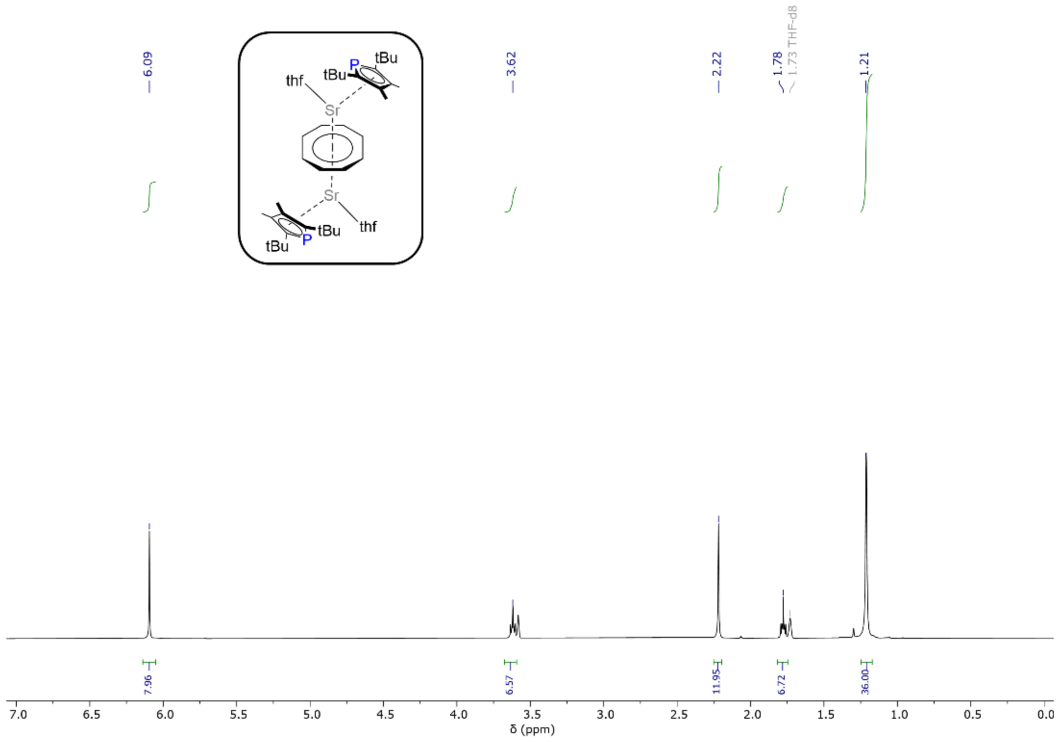

Figure S1: ^{1}H NMR spectrum (400 MHz, THF-d_8, 298 K) of **[{(Dtp)(thf)Sr}$_2${μ-C_8H_8}]**in THF-d_8.

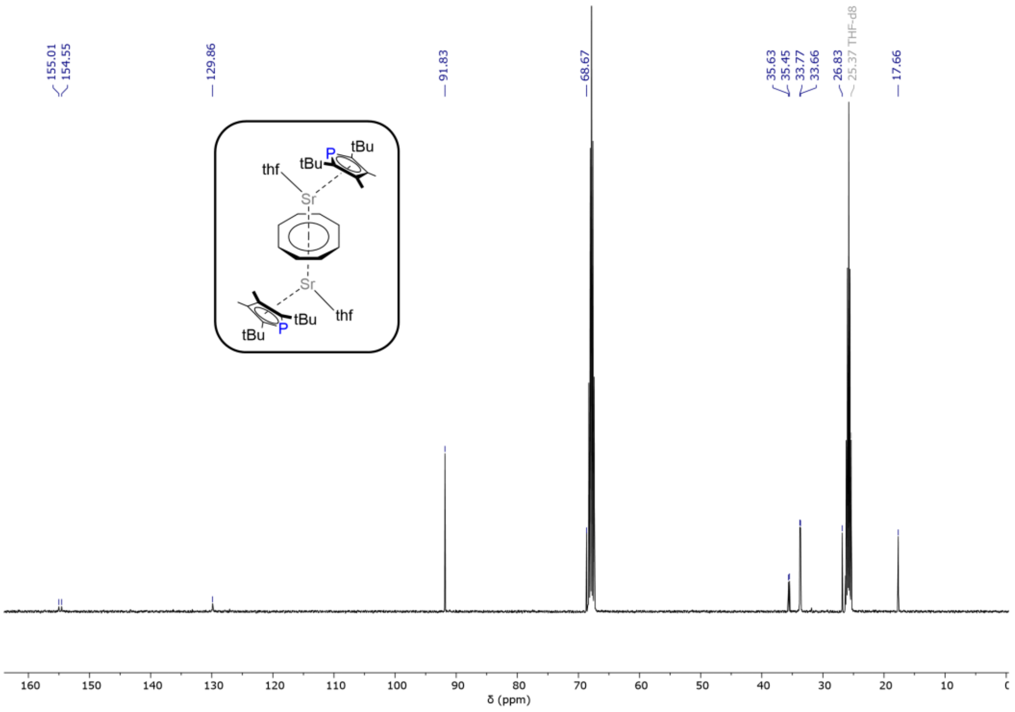

Figure S2: $^{13}C\{^{1}H\}$ NMR spectrum (100 MHz, THF-d_8, 298 K) of **[{(Dtp)(thf)Sr}$_2${μ-C_8H_8}]** in THF-d_8.

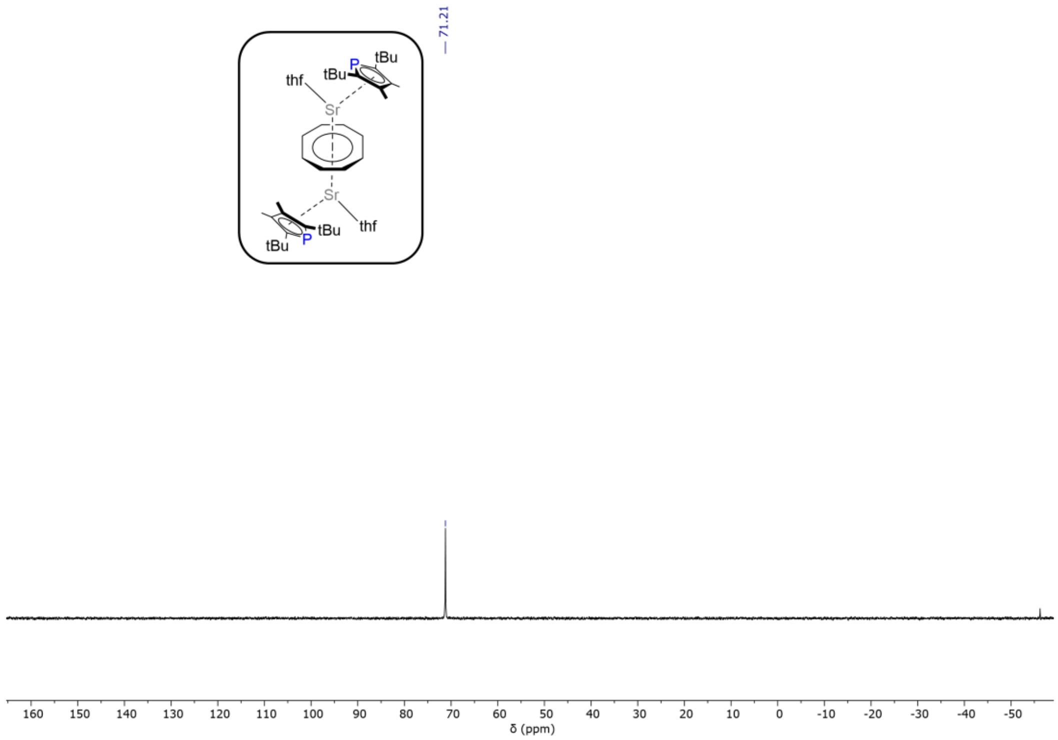

Figure S3: $^{31}P\{^{1}H\}$ NMR spectrum (162 MHz, THF-d_8, 298 K) of **[{(Dtp)(thf)Sr}$_2${μ-C_8H_8}]** in THF-d_8.

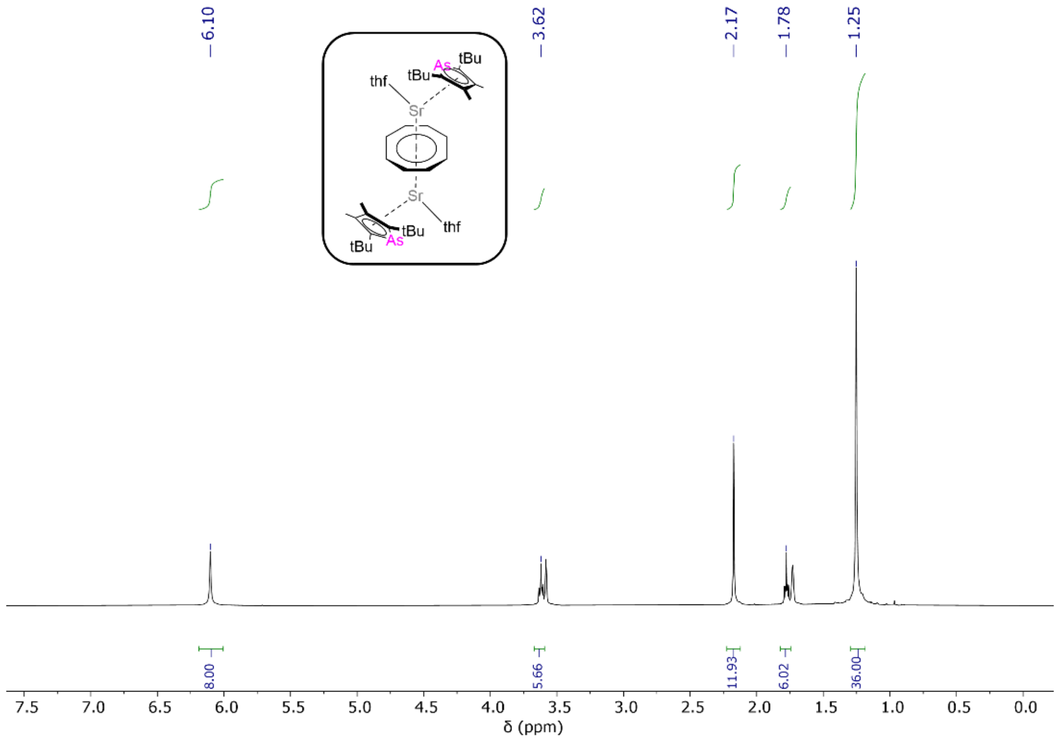

Figure S4: ^{1}H NMR spectrum (400 MHz, THF-d_8, 298 K) of **[{(Dtas)(thf)Sr}$_2${μ-C_8H_8}]** in THF-d_8.

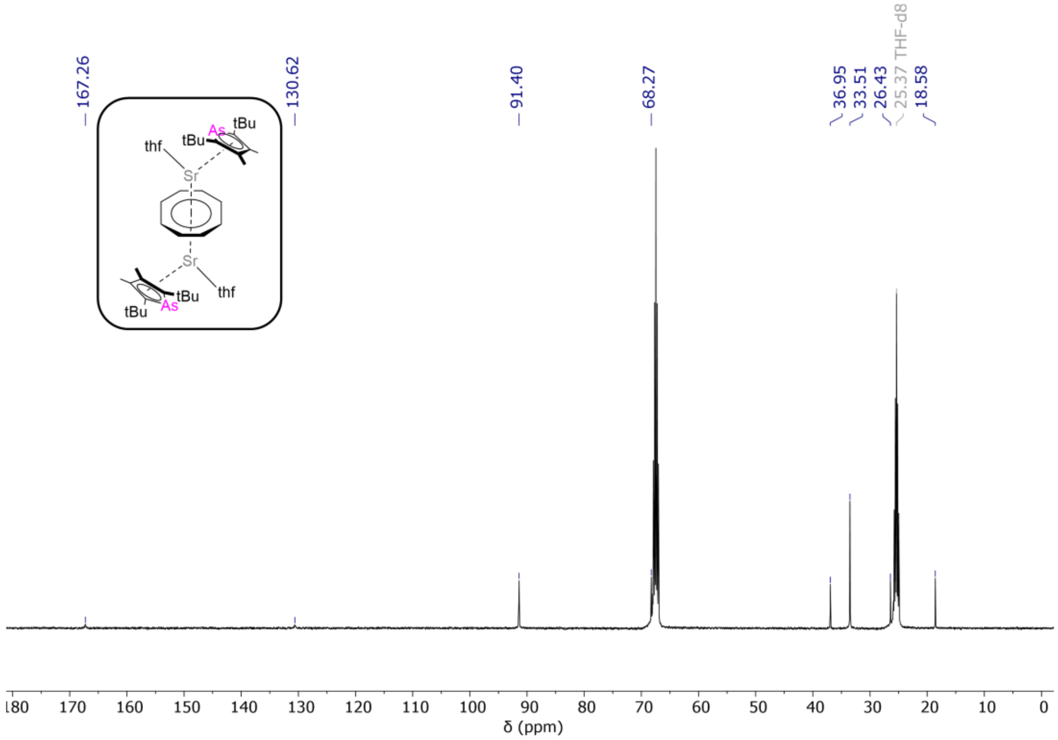

Figure S5: $^{13}C\{^{1}H\}$ NMR spectrum (100 MHz, THF-d_8, 298 K) of **[{(Dtas)(thf)Sr}$_2${μ-C_8H_8}]** in THF-d_8.

IR spectra

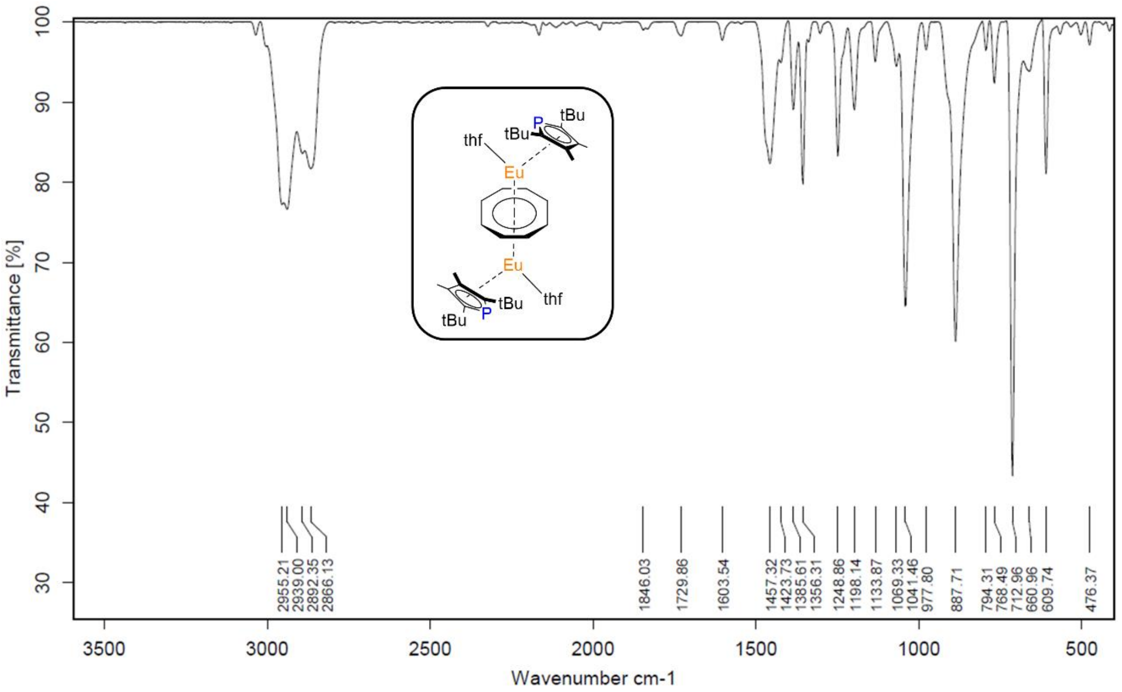

Figure S6: FT-IR (ATR) spectrum of of **[{(Dtp)(thf)Eu}$_2${μ-C_8H_8}]**.

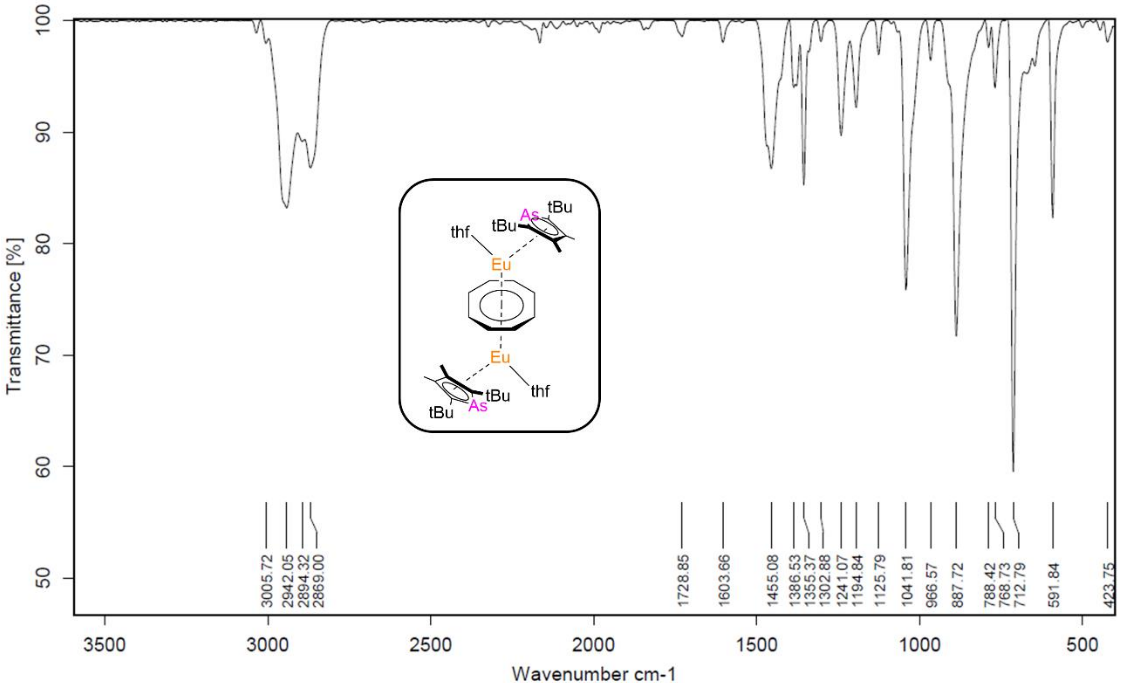

Figure S7: FT-IR (ATR) spectrum of **[{(Dtas)(thf)Eu}$_2${μ-C_8H_8}]**.

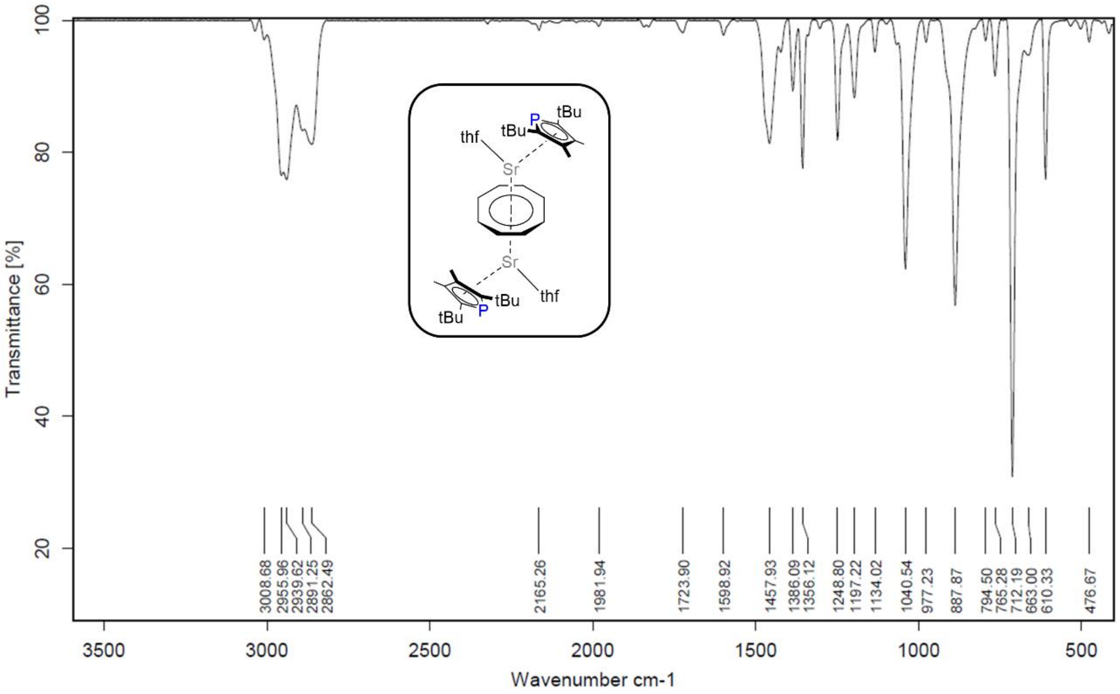

Figure S8: FT-IR (ATR) spectrum of **[{(Dtp)(thf)Sr}$_2${μ-C_8H_8}]**.

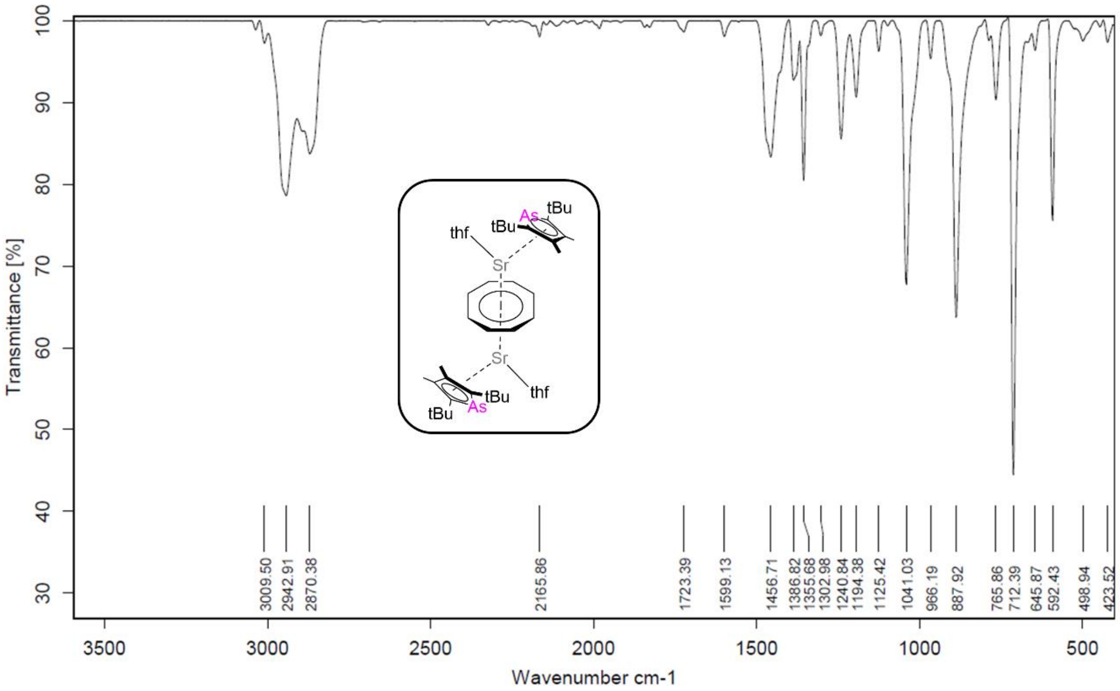

Figure S9: FT-IR (ATR) spectrum **[{(Dtas)(thf)Sr}$_2${μ-C_8H_8}]**.

X-ray crystallography

General methods

Suitable crystals for the X-ray analysis of all compounds were obtained as described above. A suitable crystal was covered in mineral oil (Aldrich) and mounted on a glass fibre. The crystal was transferred directly to the cold stream of a STOE StadiVari (100 K) diffractometer. All structures were solved by using the program SHELXS/T and Olex2.[6-8] The remaining non-hydrogen atoms were located from successive difference Fourier map calculations. The refinements were carried out by using full-matrix least-squares techniques on F^2 by using the program SHELXL.[9] The H-atoms were introduced into the geometrically calculated positions (SHELXL procedures) unless otherwise stated and refined riding on the corresponding parent atoms. In each case, the locations of the largest peaks in the final difference Fourier map calculations, as well as the magnitude of the residual electron densities, were of no chemical significance. Specific comments for each data set are given below. Summary of the crystal data, data collection and refinement for compounds are given in Table S1. Crystallographic data for the structures reported in this paper have been deposited with the Cambridge Crystallographic Data Centre as a supplementary publication no. CCDC 2306753-2306756. Copies of the data can be obtained free of charge on application to CCDC, 12 Union Road, Cambridge CB21EZ, UK (fax: (+(44)1223-336-033; email: deposit@ccdc.cam.ac.uk)

Summary of crystal data

Table S1: Crystal data, data collection and refinement for complexes **1**-**4**.

Compounds	[{(Dtp)(thf)Eu}$_2${μ-C_8H_8}] (1)	[{(Dtas)(thf)Eu}$_2${μ-C_8H_8}] (2)	[{(Dtp)(thf)Sr}$_2${μ-C_8H_8}] (3)	[{(Dtas)(thf)Sr}$_2${μ-C_8H_8}] (4)
Chemical formula	$C_{44}H_{72}Eu_2O_2P_2$	$C_{44}H_{72}As_2Eu_2O_2$	$C_{44}H_{72}O_2P_2Sr_2$	$C_{44}H_{72}As_2O_2Sr_2$
CCDC Number	2306753	2306754	2306755	2306756
Formula Mass	998.87	1086.77	870.19	958.09
Crystal system	Monoclinic	Monoclinic	Monoclinic	Monoclinic
a/Å	15.647(2)	15.6346(6)	15.6714(6)	15.6613(14)
b/Å	14.9837(10)	15.0727(4)	15.0708(8)	15.1443(8)
c/Å	19.910(2)	20.0142(7)	19.8823(9)	20.0212(14)
α/°				
β/°	110.340(9)	110.203(3)	110.598(3)	110.489(6)
γ/°				
Unit cell volume/Å^3	4377.0(8)	4426.3(3)	4395.6(4)	4448.2(6)
Temperature/K	100	100	100	100
Space group	$P2_1/c$	$P2_1/c$	$P2_1/c$	$P2_1/c$
No. of formula units per unit cell, Z	4	4	4	4
Absorption coefficient, μ/mm^{-1}	2.946	4.325	2.533	3.908
No. of reflections measured	22065	25325	7918[a]	27764
No. of independent reflections	8573	9070	7918[a]	9570
R_{int}	0.0493	0.0242	-[a]	0.0403
Final R_1 values ($I > 2\sigma(I)$)	0.0484	0.0323	0.0827	0.0509
Final $wR(F^2)$ values ($I > 2\sigma(I)$)	0.0911	0.0754	0.1852	0.1030
Final R_1 values (all data)	0.0874	0.0356	0.1385	0.0796
Final $wR(F^2)$ values (all data)	0.1041	0.0774	0.2283	0.1137
Goodness of fit on F^2	1.120	1.211	1.038	1.127

[a]merged data due to twin refinement by using Olex2 routine.[7]

Crystal structures

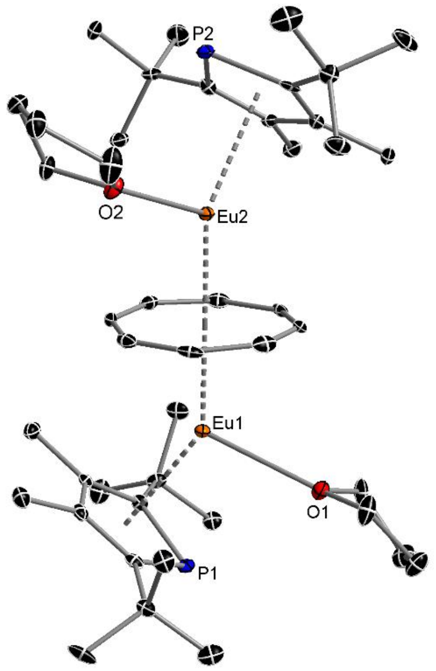

Figure S10: Molecular structure of **1** in the solid state with ellipsoids drawn at 30% probability. Hydrogen atoms are omitted for clarity. Selected bond lengths [Å] and angles [°]: Eu1-P1 3.063(2), Eu2-P2 3.082(2), Eu1-Ct$_{Dtp1}$ 2.625(3), Eu2-Ct$_{Dtp2}$ 2.633(3), Eu1-Ct$_{COT}$ 2.169(3), Eu1-C$_{COT}$ 2.813(8)-2.906(8), Eu2-C$_{COT}$ 2.801(8)-2.903(7), Eu2-Ct$_{COT}$ 2.172(3), Eu1-O1 2.575(5), Eu2-O2 2.566(5); Eu1-Ct$_{COT}$-Eu2 177.5(2), Ct$_{COT}$-Eu1-Ct$_{Dtp1}$ 144.19(2), Ct$_{COT}$-Eu2-Ct$_{Dtp2}$ 143.75(12).

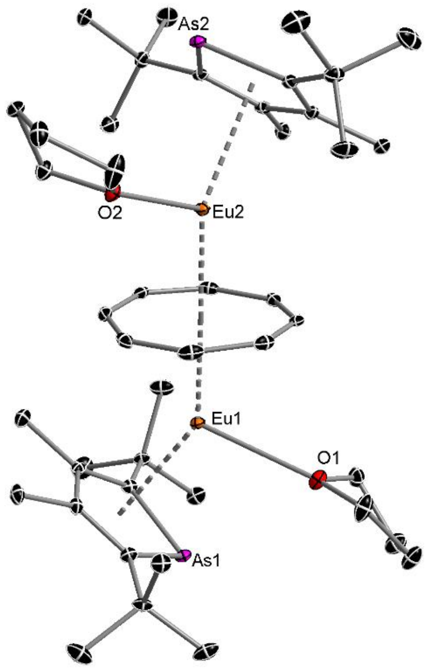

Figure S11: Molecular structure of **2** in the solid state with ellipsoids drawn at 30% probability. Hydrogen atoms are omitted for clarity. Selected bond lengths [Å] and angles [°]: Eu1-As1 3.1395(5), Eu2-As2 3.1621(5), Eu1-Ct$_{Dtas1}$ 2.645(2); Eu2-Ct$_{Dtas2}$ 2.649(2), Eu1-Ct$_{COT}$ 2.168(2), Eu1-C$_{COT}$ 2.807(5)-2.916(5), Eu2-C$_{COT}$ 2.822(5)-2.901(5), Eu2-Ct$_{COT}$ 2.175(2), Eu1-O1 2.577(3), Eu2-O2 2.571(3); Eu1-Ct$_{COT}$-Eu2 177.23(11), Ct$_{COT}$-Eu1-Ct$_{Dtas1}$ 144.51(6), Ct$_{COT}$-Eu2-Ct$_{Dtas2}$ 143.88(7).

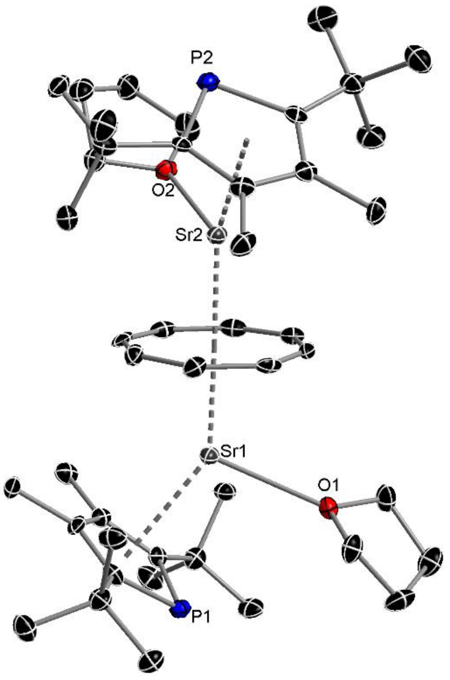

Figure S12: Molecular structure of **3** in the solid state with ellipsoids drawn at 30% probability. Hydrogen atoms are omitted for clarity. Selected bond lengths [Å] and angles [°]: Sr1-P1 3.108(3), Sr2-P2 3.085(3), Sr1-Ct_{Dtp1} 2.659(4); Sr2-Ct_{Dtp2} 2.660(4), Sr1-Ct_{COT} 2.196(4), Sr1-C_{COT} 2.825(9)-2.915(10), Sr2-C_{COT} 2.832(10)-2.916(11), Sr2-Ct_{COT} 2.191(4), Sr1-O1 2.558(7), Sr2-O2 2.545(7); Sr1-Ct_{COT}-Sr2 178.1(2), Ct_{COT}-Sr1-Ct_{Dtp1} 143.0(2), Ct_{COT}-Sr2-Ct_{Dtp2} 143.88(13).

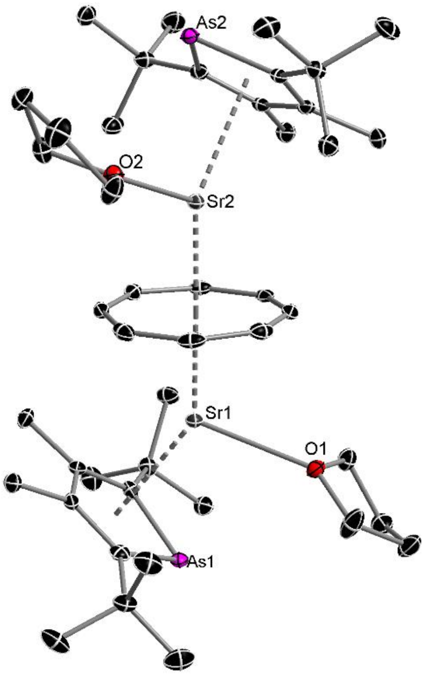

Figure S13: Molecular structure of **4** in the solid state with ellipsoids drawn at 30% probability. Hydrogen atoms are omitted for clarity. Selected bond lengths [Å] and angles [°]: Sr1-As1 3.1831(7), Sr2-As2 3.1647(7), Sr1-Ct_{Dtas1} 2.674(2); Sr2-Ct_{Dtas2} 2.669(2), Sr1-Ct_{COT} 2.198(2), Sr1-C_{COT} 2.831(5)-2.906(5), Sr2-C_{COT} 2.815(5)-2.914(5), Sr2-Ct_{COT} 2.189(2), Sr1-O1 2.550(4), Sr2-O2 2.556(4); Sr1-Ct_{COT}-Sr2 177.77(11), Ct_{COT}-Sr1-Ct_{Dtas1} 143.40(8), Ct_{COT}-Sr2-Ct_{Dtas2} 144.03(7).

Photoluminescence measurements

Photoluminescence emission (PL) and excitation (PLE) measurements in solid state and in solution were performed with a PTI QuantaMasterTM 8075-22 fluorometer with double monochromators (HORIBA Jobin Yvon GmbH, Germany). Solid samples (crystalline powders) and solutions were each sealed under inert atmosphere in NMR tubes with a J. Young valve (material Suprasil® quartz glass). The tube was placed in a glass dewar vessel (equipped with a suprasil finger on the bottom where spectroscopy takes place) which was filled with liquid nitrogen for measurements at 77 K. For emission detection, a R928 photomultiplier (250–800 nm) (HORIBA Jobin Yvon GmbH, Germany) was used. All spectra were corrected for the wavelength dependent response of the detector (in relative photon flux units) and the spectrometer. For all spectra, the instrument software FelixGX 4.9.0.10243 was used. PL and PLE spectra were recorded at the indicated wavelengths given in the Figures. For detection of the emission decay traces, the samples were excited with a Delta DiodeTM (HORIBA Jobin Yvon GmbH, Model DD-370) or a PTI XenonFlashTM (before the emission monochromator, frequency 300 MHz). For determination of the lifetimes the obtained traces were fit with an exponential decay curve using the program Origin(Pro), Version 2019 (OriginLab Corporation, USA). Quantum yields of the complexes (solid samples were used as dispersions in a thin layer of viscous mineral oil Sigma Aldrich placed between two 1 mm thin quartz plates) at ambient temperature were determined using an integrating sphere out of optical PTFE, which was installed into the sample chamber of the spectrometer, according to the method of de Mello *et al.*[10] The uncertainty of this measurement was estimated to be ±10%.

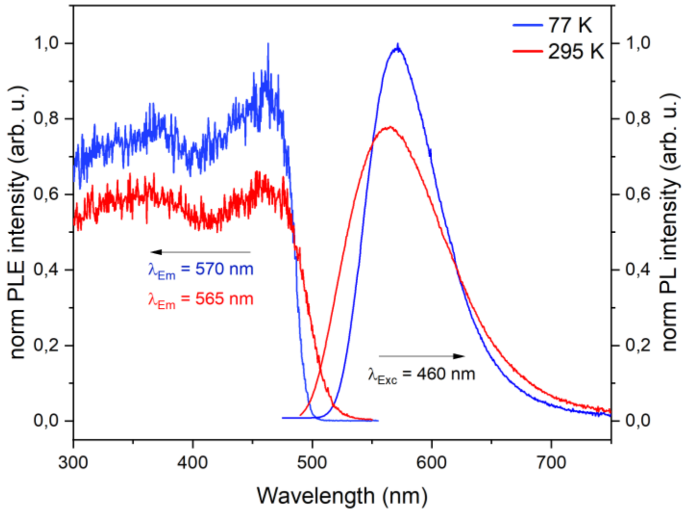

Figure S14: Photoluminescence emission (PL) and excitation (PLE) spectra of compound **1** in the solid state at 77 K (blue line) and 295 K (red line).

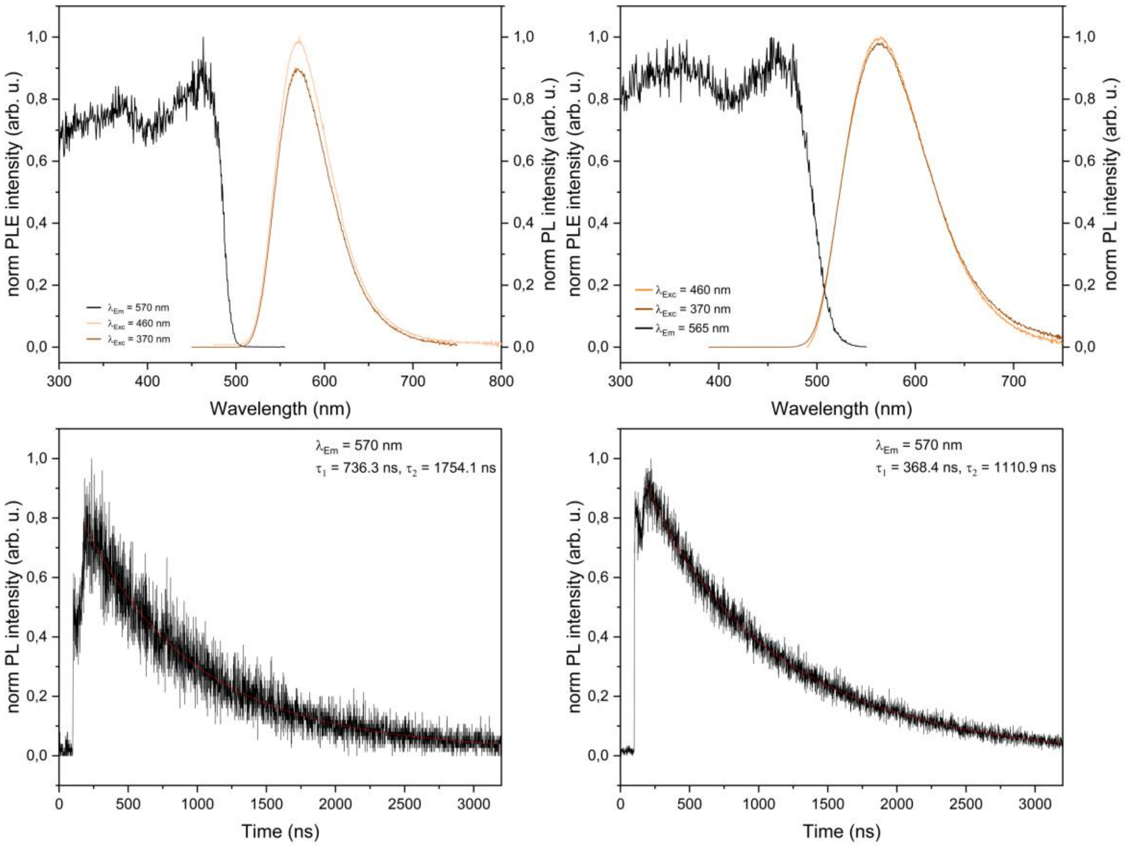

Figure S15: Top: PL and PLE spectra of **1** in the solid state at 77 K (left) and 295 K (right). Bottom: Fluorescence decay times in the solid state for **1** at 77 K (left) and 295 K (right).

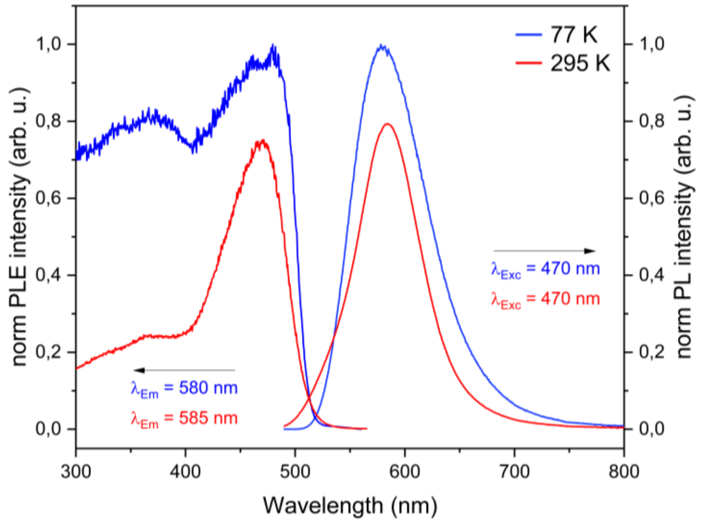

Figure S16: Photoluminescence emission (PL) and excitation (PLE) spectra of compound **1** in a THF solution at 77 K (blue line) and 295 K (red line).

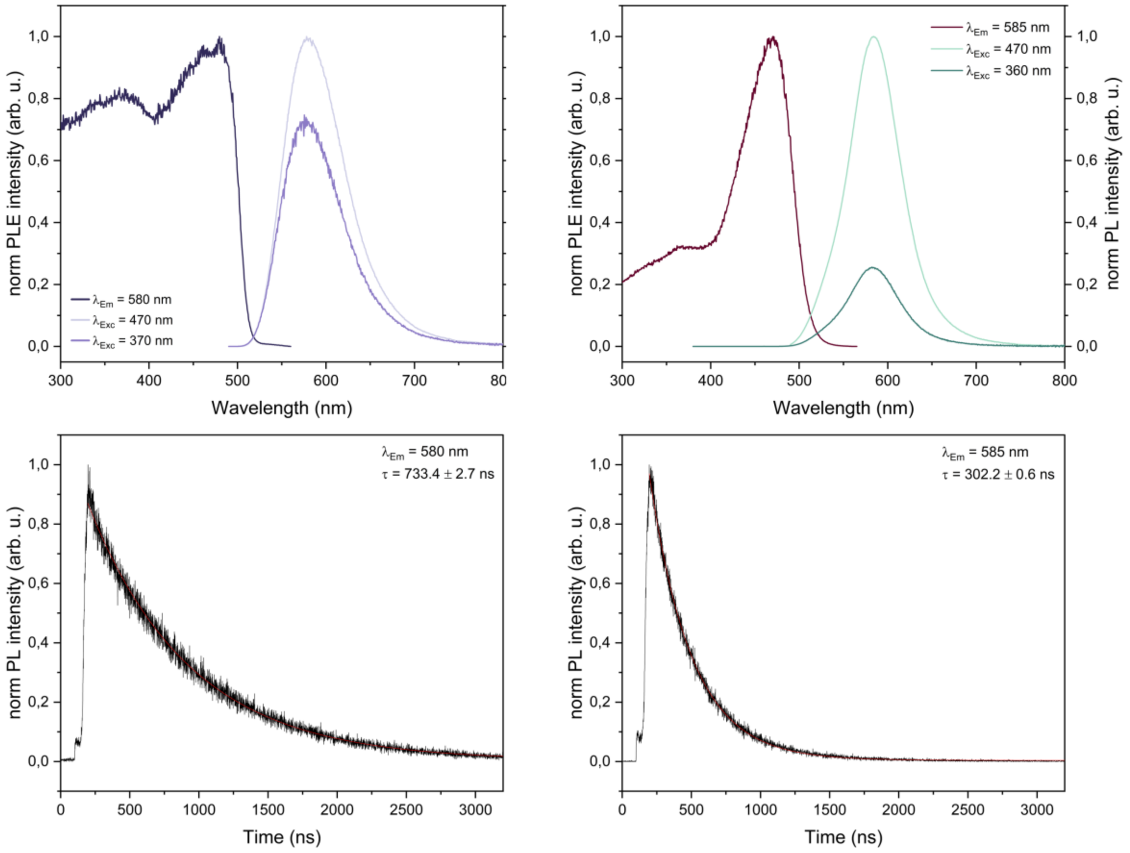

Figure S17: Top: PL and PLE spectra of **1** in a THF solution at 77 K (left) and 295 K (right). Bottom: Fluorescence decay times in a THF solution of **1** at 77 K (left) and 295 K (right).

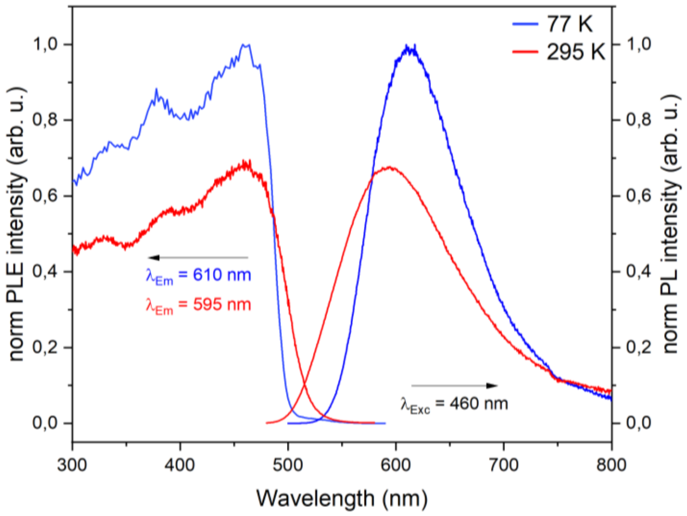

Figure S18: Photoluminescence emission (PL) and excitation (PLE) spectra of compound **2** in the solid state at 77 K (blue line) and 295 K (red line).

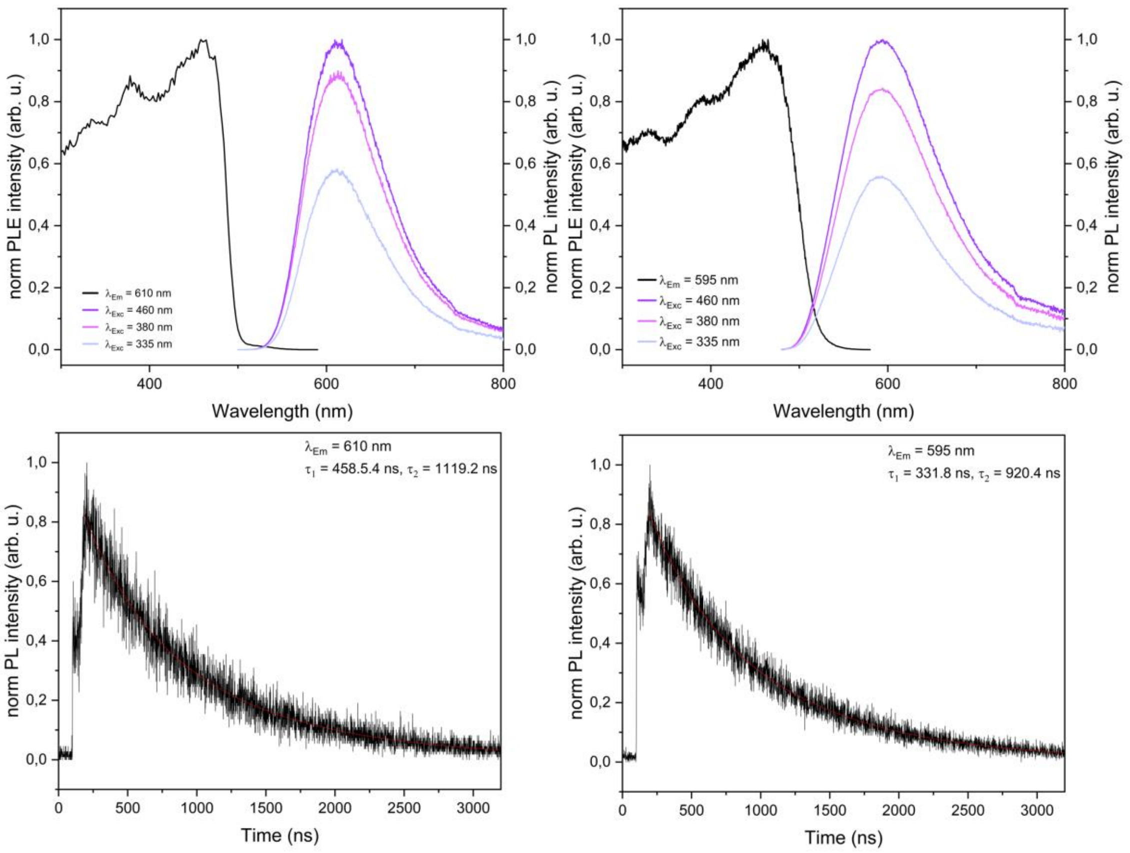

Figure S19: Top: PL and PLE spectra of **2** in the solid state at 77 K (left) and 295 K (right). Bottom: Fluorescence decay times in the solid state for **2** at 77 K (left) and 295 K (right).

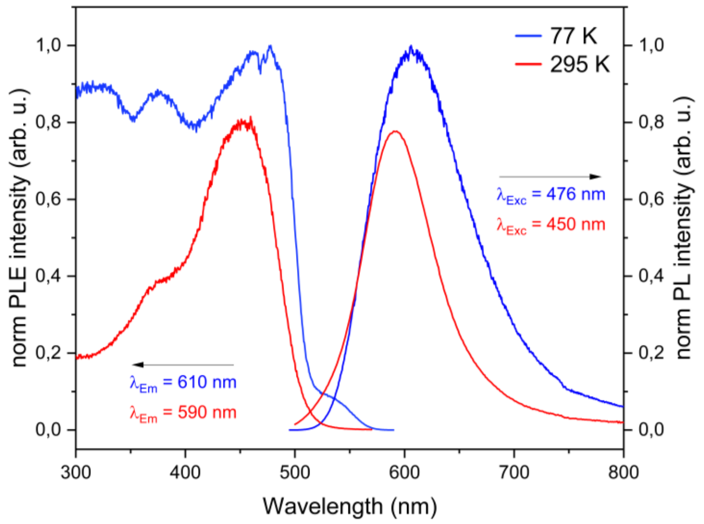

Figure S20: Photoluminescence emission (PL) and excitation (PLE) spectra of compound **2** in a THF solution at 77 K (blue line) and 295 K (red line).

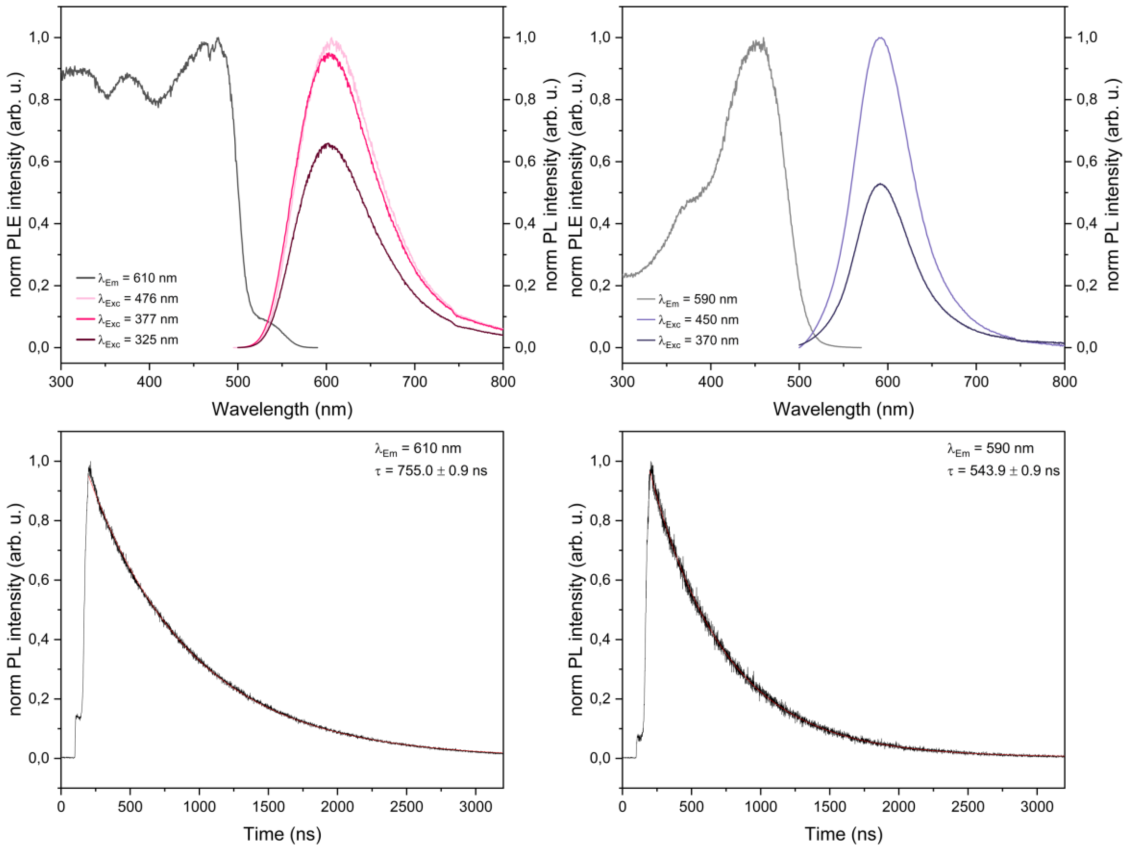

Figure S21: Top: PL and PLE spectra of **2** in a THF solution at 77 K (left) and 295 K (right). Bottom: Fluorescence decay times in a THF solution of **2** at 77 K (left) and 295 K (right).

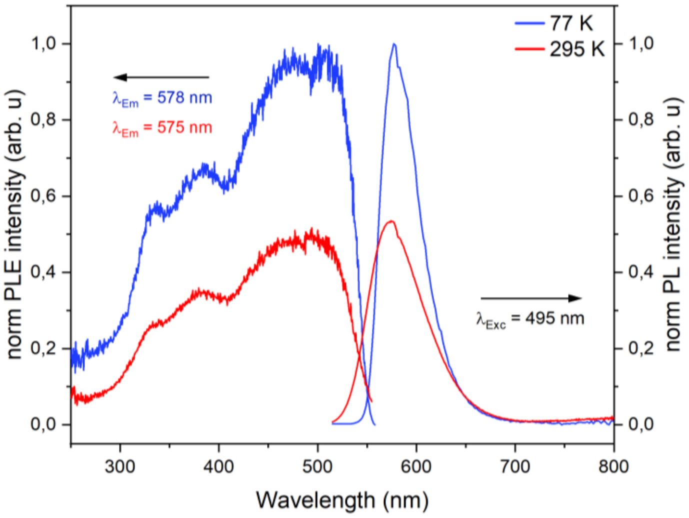

Figure S22: Photoluminescence emission (PL) and excitation (PLE) spectra of compound [{(Cp*)(thf)$_2$Eu}$_2${μ-η^8:η^8-C_8H_8}] in the solid state at 77 K (blue line) and 295 K (red line).

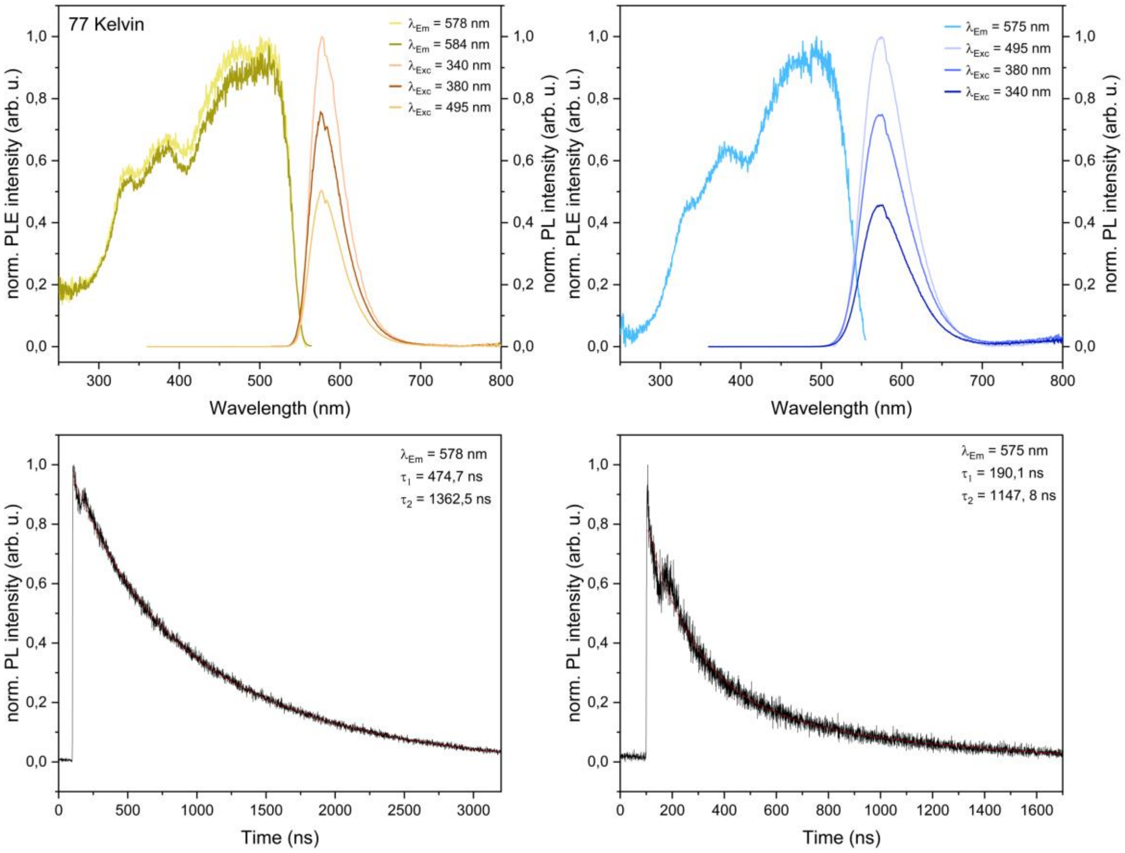

Figure S23: Top: PL and PLE spectra in the solid state of [{(Cp*)(thf)$_2$Eu}$_2${μ-η^8:η^8-C_8H_8}] at 77 K (left) and 295 K (right). Bottom: Fluorescence decay times in the solid state for [{(Cp*)(thf)$_2$Eu}$_2${μ-η^8:η^8-C_8H_8}] at 77 K (left) and 295 K (right).

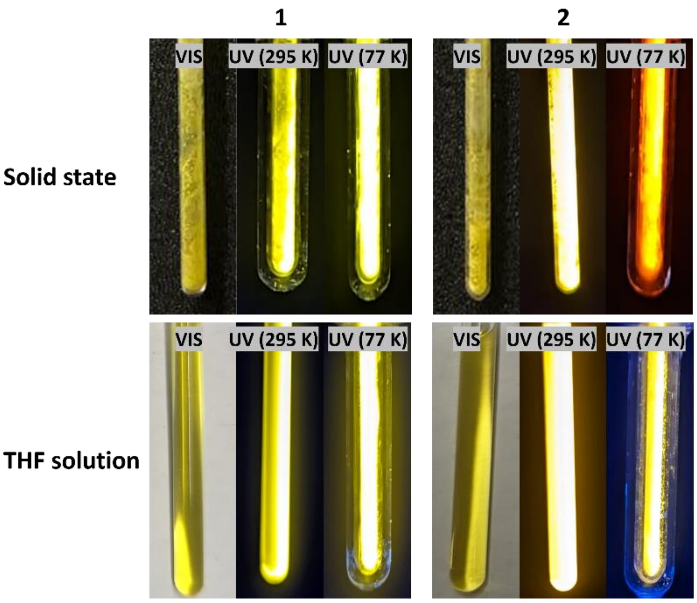

Figure S24: Pictures of **1** and **2** in the solid state and in THF solution under visible and UV-light.

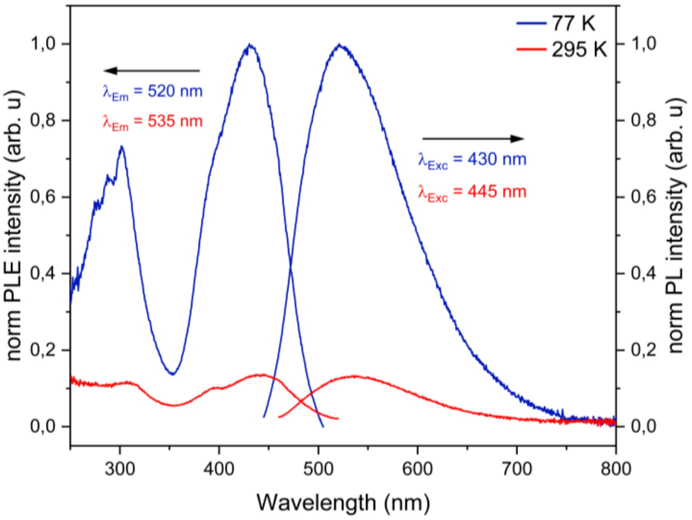

Figure S25: Photoluminescence emission (PL) and excitation (PLE) spectra of compound **3** in the solid state at 77 K (blue line) and 295 K (red line).

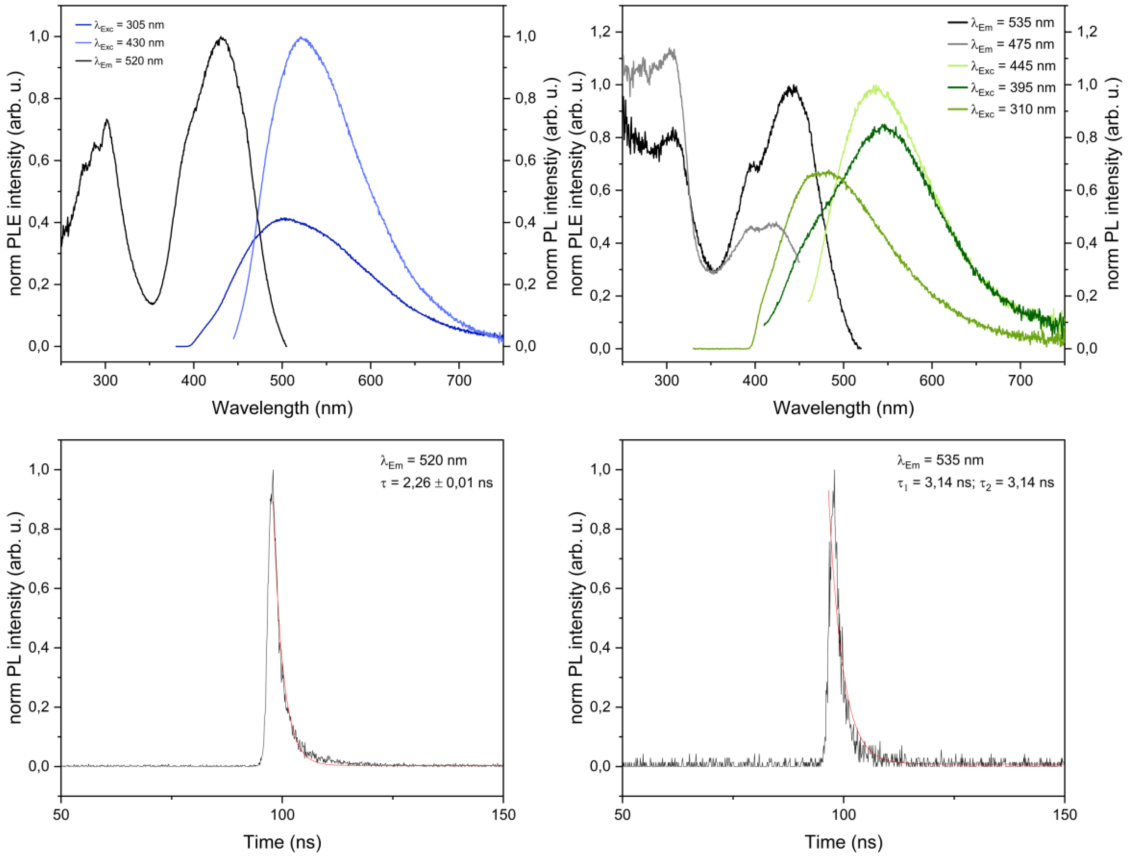

Figure S26: Top: PL and PLE spectra of **3** in the solid state at 77 K (left) and 295 K (right). Bottom: Fluorescence decay times in the solid state for **3** at 77 K (left) and 295 K (right).

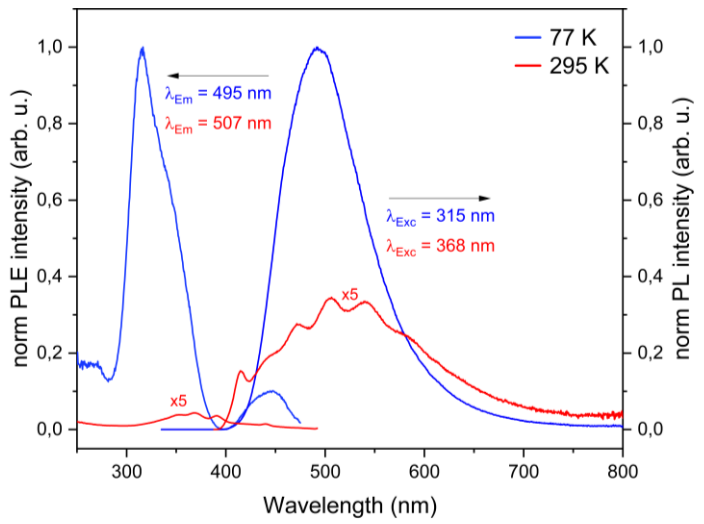

Figure S27: Photoluminescence emission (PL) and excitation (PLE) spectra of compound **3** in a THF solution at 77 K (blue line) and 295 K (red line).

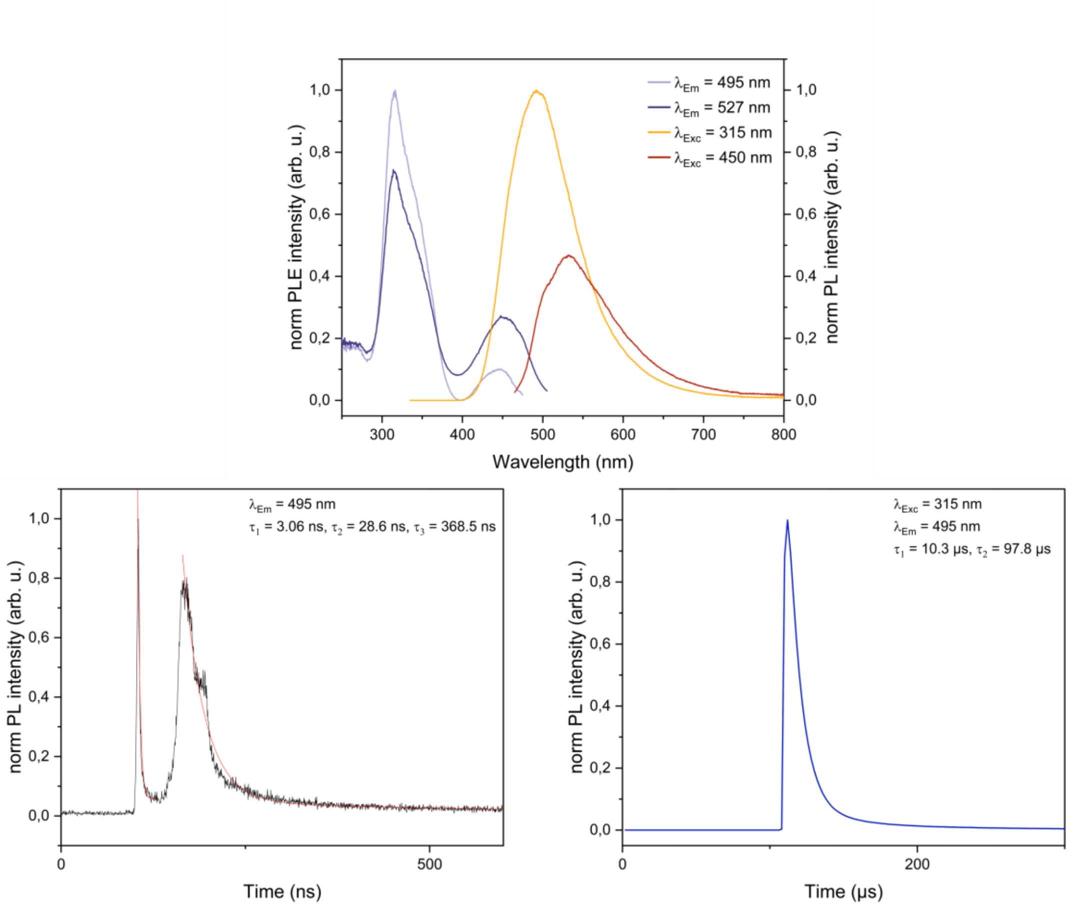

Figure S28: Top: PL and PLE spectra of **3** in a THF solution at 77 K. Bottom: Fluorescence (left) and phosphorescence (right) decay times in a THF solution of **3** at 77 K.

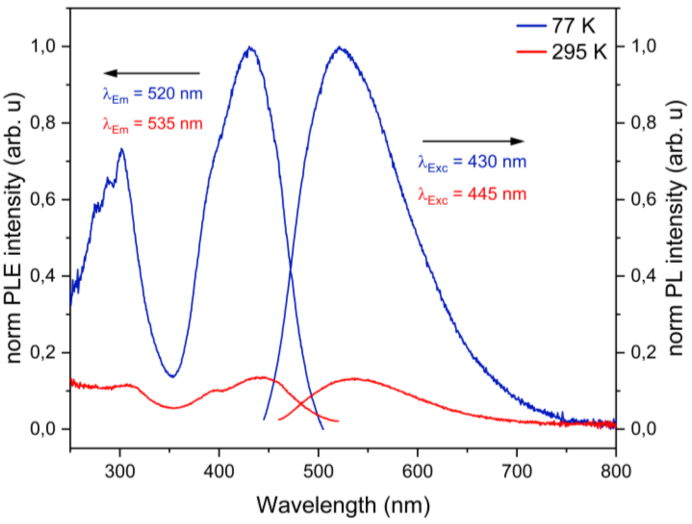

Figure S29: Photoluminescence emission (PL) and excitation (PLE) spectra of compound **4** in the solid state at 77 K (blue line) and 295 K (red line).

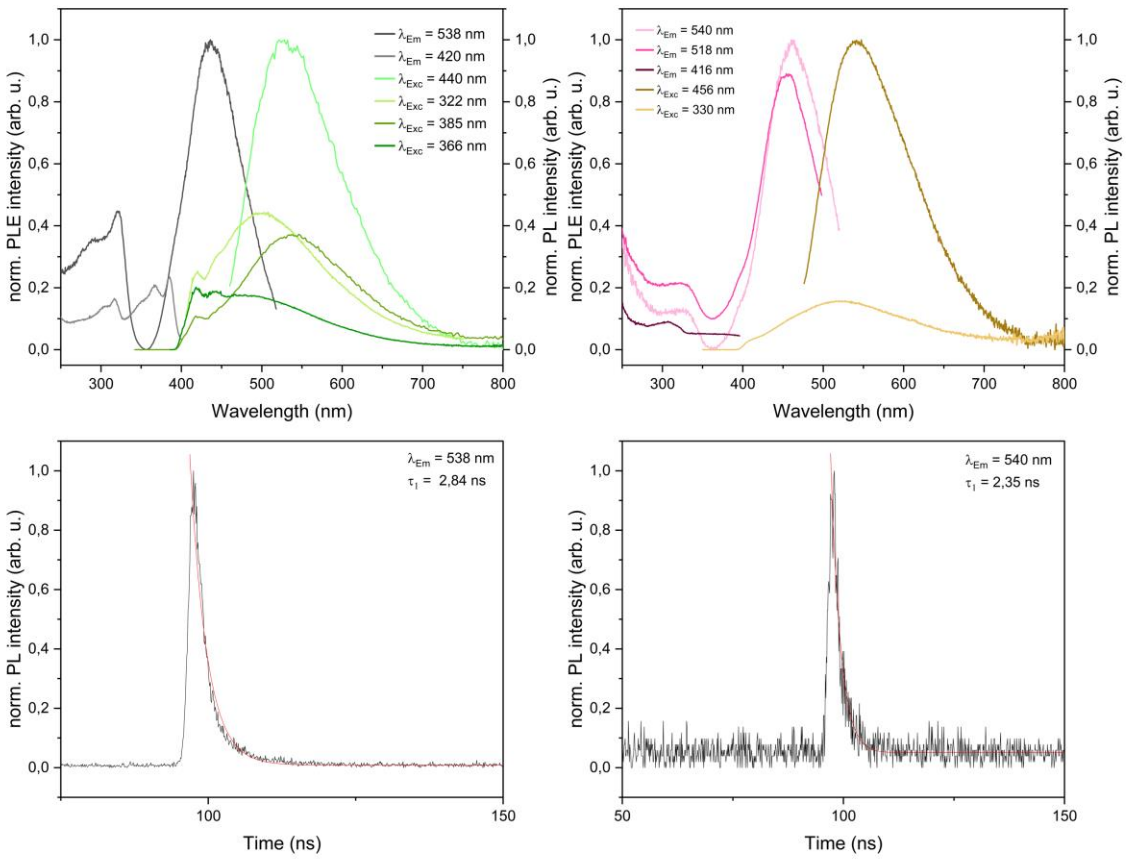

Figure S30: Top: PL and PLE spectra in the solid state of **4** at 77 K (left) and 295 K (right). Bottom: Fluorescence decay times in the solid state for **4** at 77 K (left) and 295 K (right).

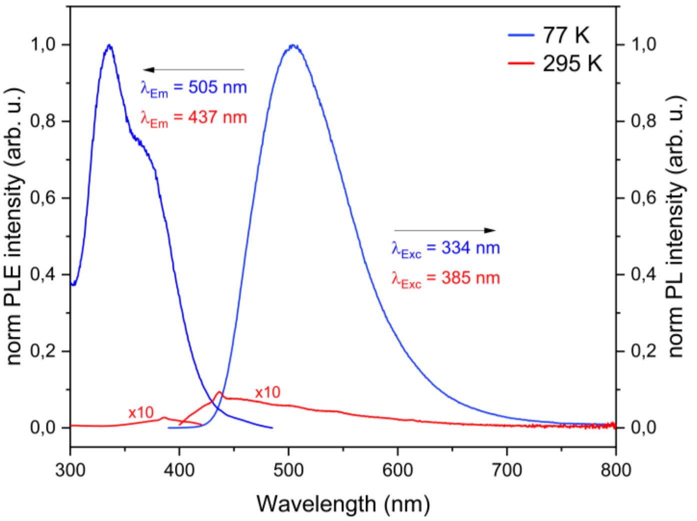

Figure S31: Photoluminescence emission (PL) and excitation (PLE) spectra of compound **4** in a THF solution at 77 K (blue line) and 295 K (red line).

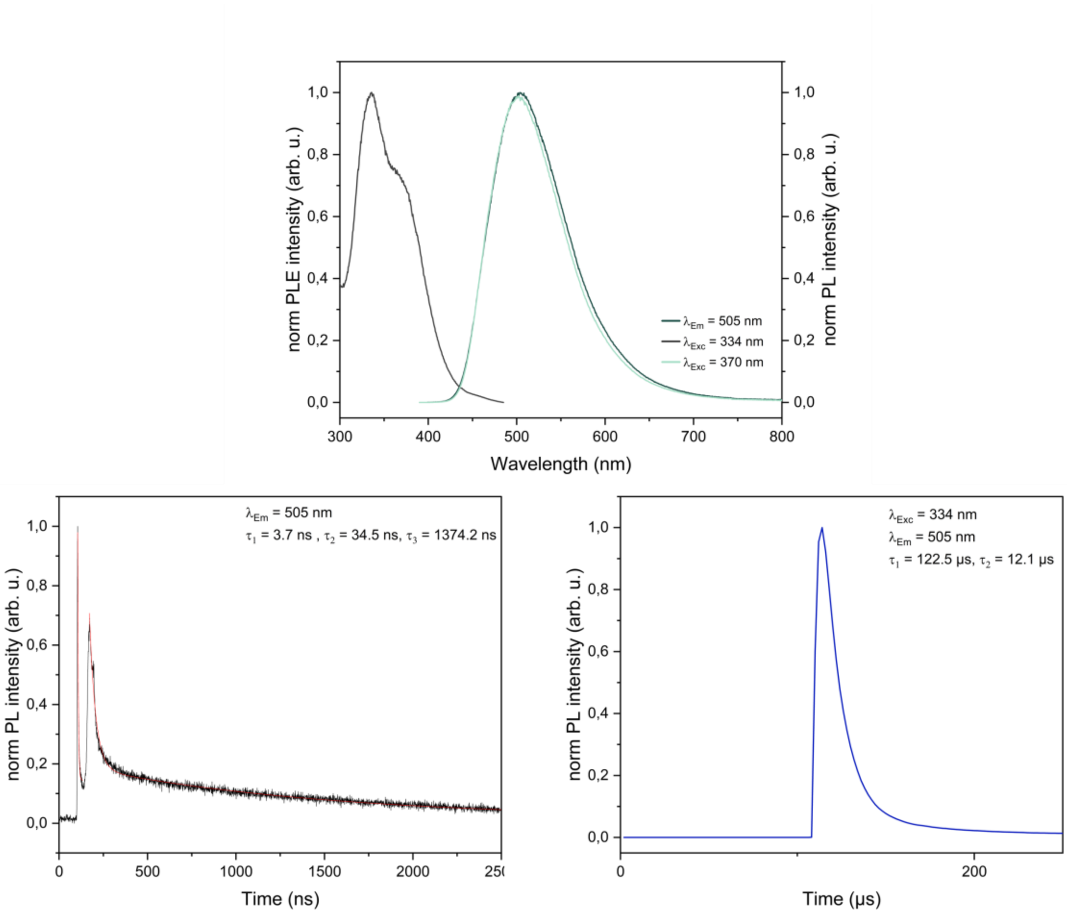

Figure S32: Top: PL and PLE spectra of **4** in a THF solution at 77 K. Bottom: Fluorescence (left) and phosphorescence (right) decay times in a THF solution of **3** at 77 K.

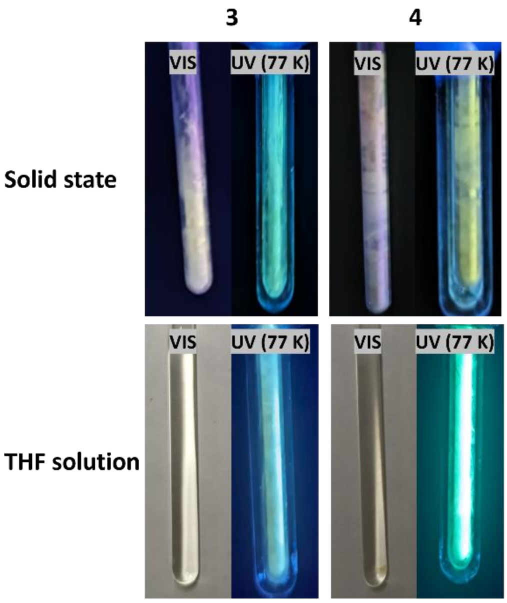

Figure S33: Pictures of **3** and **4** in the solid state and in THF solution under visible and UV-light.

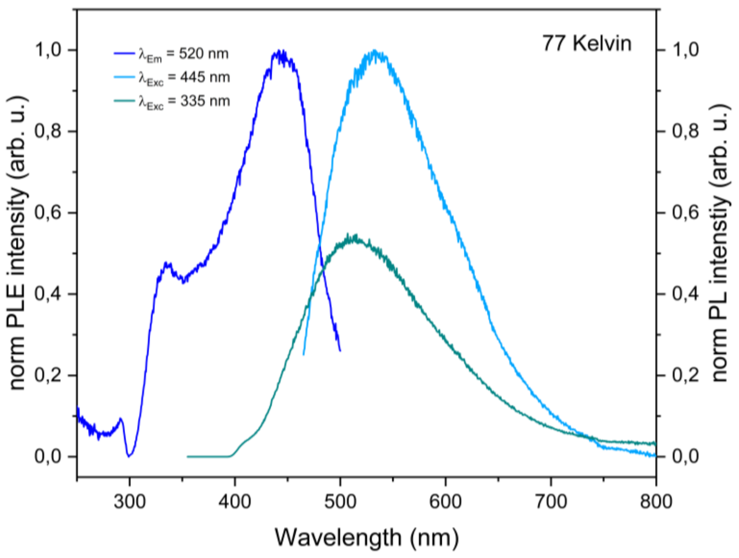

Figure S34: Photoluminescence emission (PL) and excitation (PLE) spectra of K(Dtp) in the solid state at 77 K.

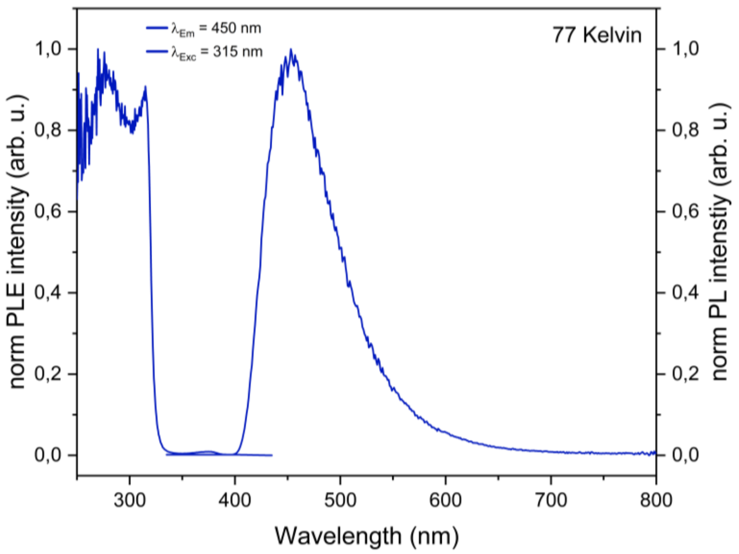

Figure S35: Photoluminescence emission (PL) and excitation (PLE) spectra of K(Dtas) in the solid state at 77 K.

K(dtp)

K(dtas)

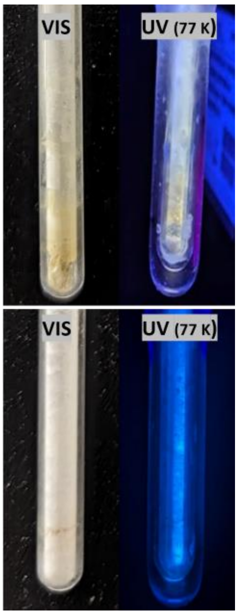

Figure S36: Pictures of K(Dtp) (top) and K(Dtas) (bottom) in the solid state under visible (left) and UV-light (right).

UV-Vis measurements

UV-Vis spectra were recorded on a *Ocean Optics* USS-ISS-UV-/Vis-spectrophotometer at concentrations of around 10^{-4} mmol/mL. For air-sensitive samples, cuvettes equipped with a J. Young valve were used.

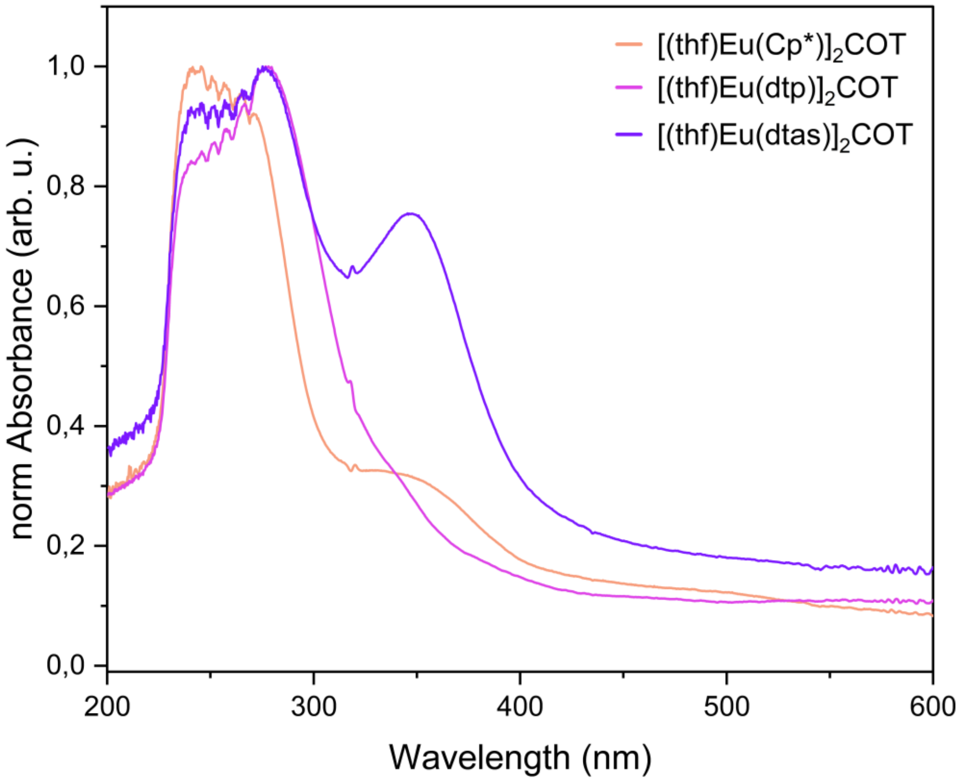

Figure S37: UV-Vis Spectra of **1** (pink line), **2** (blue line) and [{(Cp*)(thf)$_2$Eu}$_2${μ-η^8:η^8-C_8H_8}] (orange line) in THF solution (concentrations: 200 μM)

Theoretical calculations

Quantum chemical calculations at density functional theory (DFT) level were performed with the program suite TURBOMOLE.[11,12] All structures were optimized with the PBE0 hybrid functional.[13,14] def2-TZVP basis sets[15] together with the effective core potentials (ECP) for Sr and Eu were used.[16,17]

The resolution of the identity approximation for the Coulomb part (RI-*J*)[18] in combination with the corresponding auxiliary basis sets as well as the multipole-accelerated RI-*J* approximation (MARI-*J*)[19] were employed.

For ground state and response calculations, self-consistent field (SCF) thresholds were set to 10^{-8} E_h. For response calculations, the convergence criterion for the root mean square of the density matrix was set to 10^{-7} E_h. Medium sized grids (gridsize 3) for the numerical integration of the exchange-correlation terms were used in all calculations.[20] Difference densities were analyzed using the python script PANAMA (Peak Analyzing Machine).[21]

a)

b)

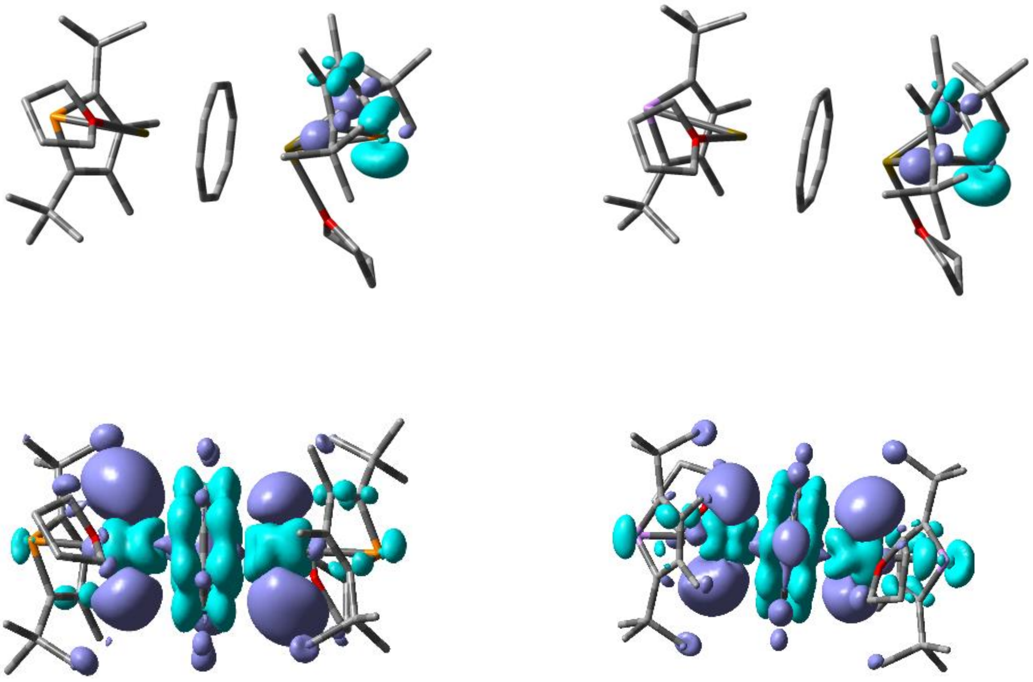

Figure S38: a) Non-relaxed difference electron densities approximating the fluorescence of **3** (left) and **4** (right) (iso values ±0.006 a.u.); b) Non-relaxed difference electron density describing the first two electronic excitations (weighted by their oscillator strength) of **1** (left) and **2** (right) (iso values ±0.0002 a.u.) from the ground states. Blue indicates a surplus of electron density for the ground state, violet for the excited state.

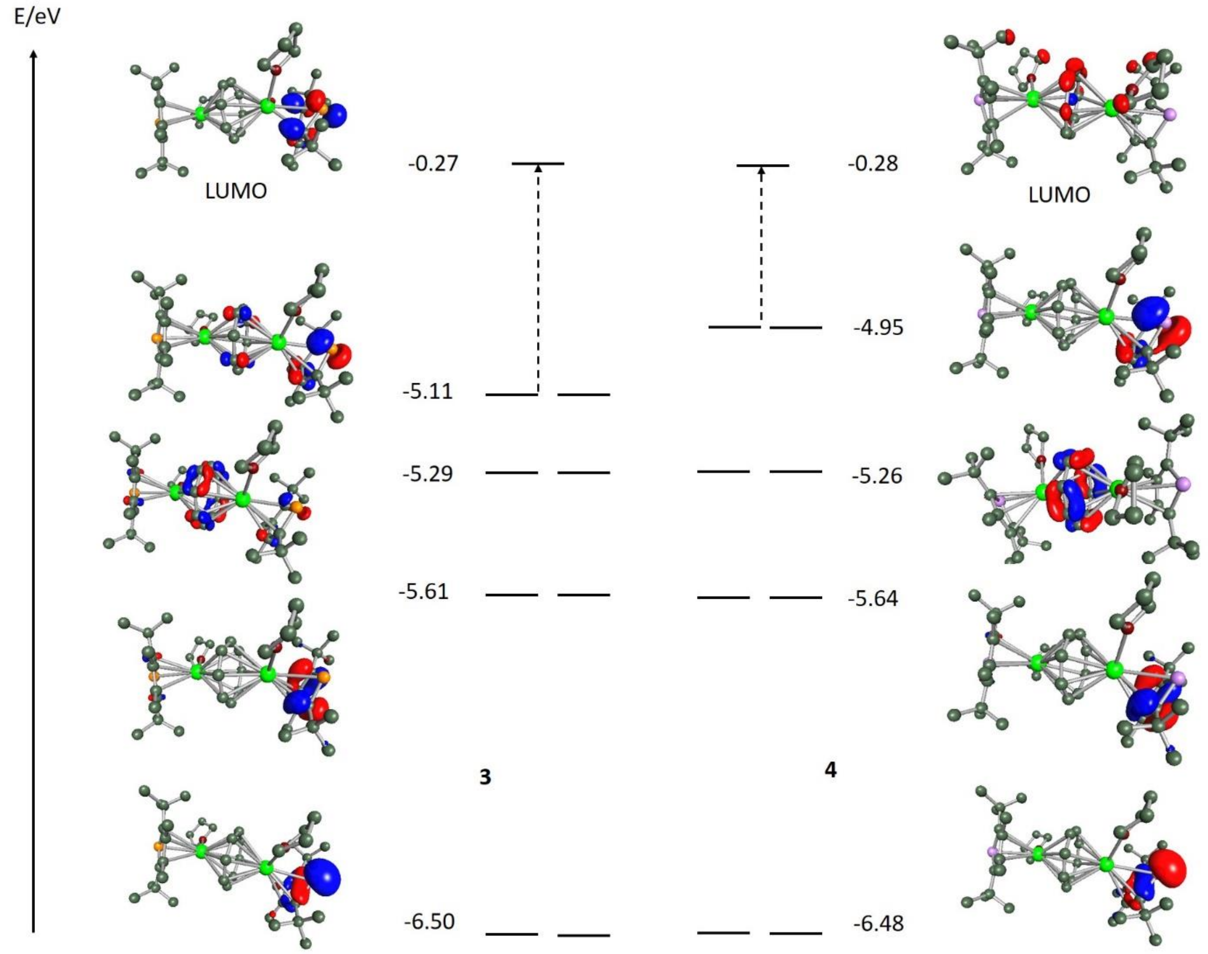

Figure S39: MO diagrams of the ground states of **3** and **4** (RI-DFT, PBE0, def2-TZVP). The HOMO is represented by the highest π-MO of the phospholyl or arsolyl ligand, respectively.

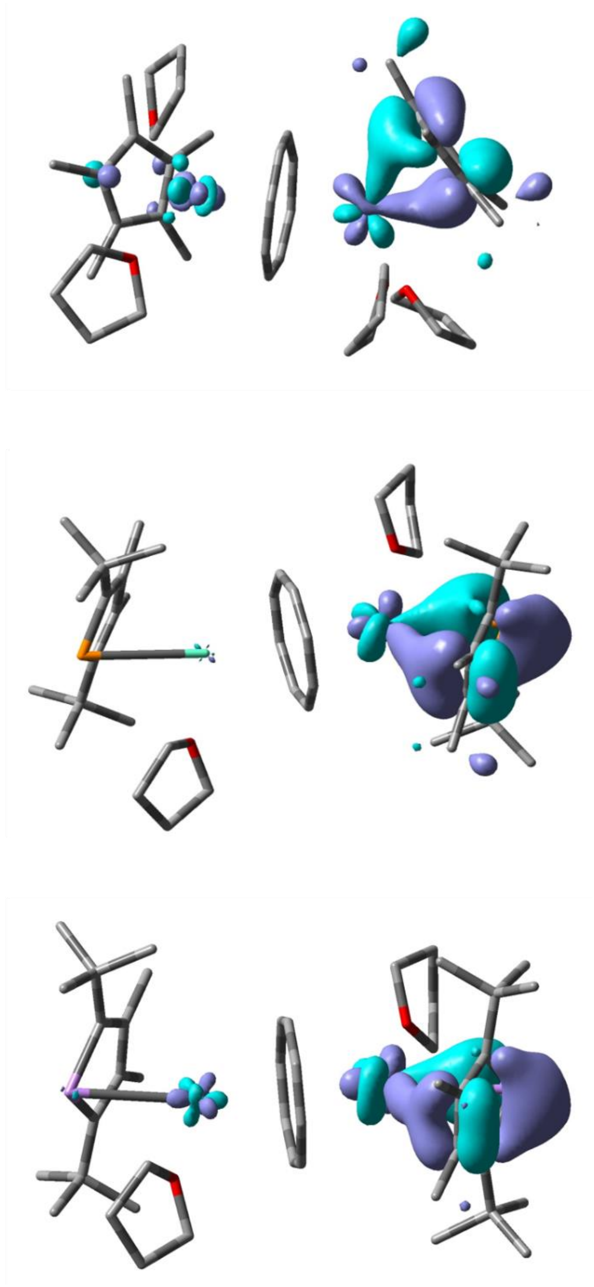

a)

b)

c)

Figure S40: Isosurface plots representing the highest π-MO (α-spin) of the Cp*-, phospholyl or arsolyl ligand of a) [{(Cp*)(thf)$_2$Eu}$_2${μ-η^8:η^8-C$_8$H$_8$}], b) **1** and c) **2** (iso values ±0.004 a.u.).

Table S2: MO energies of the relevant occupied and unoccupied (depicted in yellow or green, respectively) π-MOs of the ligands $C_5H_5^-$, $PC_4H_4^-$ and $AsC_4H_4^-$.

			C5H5(-)						PC4H4 (-)						AsC4H4 (-)		
15	1	e2"		7.599	eV	27	9	b1		6.859	eV	36	3	a2		6.896	eV
						26	2	a2		6.829	eV	35	11	b1		6.831	eV
14	4	e2'		7.506	eV	25	8	b1		6.351	eV	34	10	b1		6.249	eV
13	4	e1'		7.101	eV	24	12	a1		6.046	eV	33	16	a1		6.005	eV
12	4	a1'		6.42	eV	23	4	b2		5.563	eV	32	6	b2		5.296	eV
11	1	e1"	4	0.126	eV	22	3	b2	2	-0.249	eV	31	5	b2	2	-0.076	eV
						21	1	a2	2	-0.875	eV	30	2	a2	2	-0.966	eV
						20	11	a1	2	-2.022	eV	29	15	a1	2	-2.072	eV
						19	7	b1	2	-4.032	eV	28	9	b1	2	-3.588	eV
10	1	a2"	2	-4.155	eV	18	2	b2	2	-4.454	eV	27	4	b2	2	-4.358	eV
9	3	e2'	4	-4.337	eV	17	10	a1	2	-5.164	eV	26	14	a1	2	-5.245	eV
8	3	e1'	4	-4.812	eV	16	6	b1	2	-5.605	eV	25	8	b1	2	-5.744	eV
7	3	a1'	2	-8.602	eV	15	9	a1	2	-8.148	eV	24	13	a1	2	-8.208	eV
6	2	e2'	4	-9	eV	14	5	b1	2	-9.297	eV	23	7	b1	2	-9.181	eV

Table S3: Calculated total energies of **1**, **2**, **3**, **4** and [{(Cp*)(thf)$_2$Eu}$_2${μ-η^8:η^8-C_8H_8}] (RI-DFT, PBE0, def2-TZVP, TD-DFT).

	E_{tot}/Hartree ground state	E_{tot}/Hartree excited state (S_1)	E_{tot}/Hartree ground state (geometry of excited state).	fluorescence/nm
**1**	-3972.312704			
**2**	-7761.039384			
**3**	-2612.967028	-2612.825615	-2612.915755	505.5
**4**	-6401.693780	-6401.565374	-6401.640303	608.1
**[{(Cp*)(thf)$_2$Eu}$_2${μ-η^8:η^8-C_8H_8}]**	-3948.686264			

Table S4: Cartesian Coordinates of **1**, **2**, **3**, **4**, [{(Cp*)(thf)$_2$Eu}$_2${μ-η^8:η^8-C$_8$H$_8$}] (given in a. u.)

1, RKS ground state

15.65570076429490	14.96644963340245	7.95605885160230	eu
11.56658763811245	18.95172132106928	8.57769949156811	p
11.36727522772216	12.50675062775792	6.70412133254113	o
13.60259328680759	19.64429527016687	6.02247774736097	c
16.01257039437764	20.32344333662529	6.90234199134929	c
16.23901519079203	20.20956197026165	9.59103165317255	c
13.99676434294609	19.46949417403236	10.80312375440450	c
22.11664498138740	9.64092512602262	8.00644639730434	eu
17.25075111989240	10.14121753949675	5.80825318292870	c
18.67338646904613	11.98056387148243	4.50622455711085	c
20.22637901304819	14.01710770883897	5.24710308537145	c
20.99111141301534	15.03371695130363	7.58610187035944	c
20.54332826205394	14.45055631318443	10.14112202029196	c
19.10719250176171	12.62027246775709	11.44098703621784	c
17.52364515251035	10.60901556717381	10.70070703232063	c
16.75999015935987	9.58932128610637	8.36190386719501	c
9.32828499214825	12.25836755255812	8.44265120171539	c
10.77316290356176	10.89421303157390	4.63823637536595	c
12.67851098054005	19.67127567800873	3.28661473855713	c
18.17764597653242	21.33268325660044	5.35893231338485	c
18.63002699890868	21.10963850664712	10.83479911687842	c
13.50767060741611	19.36410890440949	13.64836588327882	c
26.15027338073061	5.57253451085359	7.58124895775257	p
26.45155509802251	12.25613711875937	8.27354479455562	o
23.56031403308544	4.80422636391023	5.62722368138002	c
21.41306937792918	4.24663309043917	7.08525005533724	c
21.83738922541349	4.47917681480797	9.74209132161378	c
24.30988005609411	5.23543497538323	10.34528710515051	c
16.30046148316182	8.84661599707228	4.53395606393838	h
18.55512636413509	11.77595362515466	2.46935360311056	h
21.00637667079696	15.03743362987196	3.64786351778557	h
22.24757664765414	16.63898937639870	7.36082645897848	h
21.50587042497813	15.73373612723907	11.41837182589193	h
19.23674856124945	12.81548819052432	13.47739257051705	h
16.73050624770110	9.59994934085679	12.30043658517446	h
15.49683326745217	7.98920015431831	8.58632326026036	h
9.55856756031538	10.49636914271887	9.51622400595613	h
9.40568294030988	13.87200359562691	9.72064395276059	h
6.96573528290011	12.19895531148105	6.80498565373454	c
11.86725225454330	11.52039915726722	3.00878257504497	h
11.33711309178989	8.95308228600019	5.10046235302764	h
7.90949308096009	11.09653154081762	4.28238532128089	c
12.73529469135668	22.36926118546500	2.22403313399345	c
9.93705401195401	18.76240254904204	3.12282165184952	c
14.24763254680929	17.90485822954288	1.60462294682660	c
17.81056542497657	21.29862423049634	3.33984571518203	h
18.58264427978559	23.29837253738339	5.87075403355112	h
19.92071568963080	20.26826126123547	5.68144362873823	h
20.30579823088496	20.43276906146413	9.83639292735858	h
18.73072625389635	23.18002854929599	10.82542601911810	h
18.79801398253113	20.50398292580058	12.79089367764960	h
13.93488577598296	21.97638181549542	14.83359662279866	c
10.76199630564640	18.63679948919067	14.19766048284608	c
15.17341262015300	17.39604753808304	14.97053670347991	c

27.33180162669988	13.90192974026576	10.22280776929689	c
28.23260381656067	12.20724538399511	6.22625221502497	c
23.83452298218473	4.55725229097794	2.76213469806784	c
18.93880975899605	3.21867496662004	6.13936846872617	c
19.79472202617007	3.70625367348593	11.56274107579732	c
25.43751244214929	5.56566519521136	12.98287178638654	c
5.46715743743220	11.07154151570211	7.65950130409465	h
6.25057757408043	14.11383088366678	6.54257234350806	h
7.08490122240023	9.25211815065899	3.87439852563994	h
7.44315333471781	12.34777863851053	2.71334284436149	h
14.63174124191286	23.17185765321198	2.19155548590343	h
12.00060442431816	22.39599852238760	0.29007482251897	h
11.55222249684066	23.60573461033201	3.37856644674762	h
8.67376613110449	19.98341089060538	4.20548309793503	h
9.31056680081230	18.77197169129861	1.15271528084732	h
9.74105947157304	16.84332227234703	3.85944316259904	h
14.05748973423094	15.93936840599763	2.22887695507800	h
13.57902519366693	17.98518883173586	-0.35124863769659	h
16.25545353857797	18.36442162824955	1.60216593359039	h
12.71572862422052	23.37550212359740	13.92896977648009	h
13.47589620361894	21.93026049595736	16.85018395288904	h
15.88161459970094	22.62073295148297	14.64421590265686	h
10.30327145536348	16.76944798537195	13.44231704101127	h
10.44660857030430	18.58957931991838	16.23975280790239	h
9.44197857304343	19.99091154840825	13.37174989121800	h
14.74792434965915	15.49402514520454	14.27716266735179	h
17.18645522730152	17.72471628267611	14.68230144522799	h
14.81594243324050	17.40471010741520	17.00810252769316	h
26.31605609698380	15.70403503241274	10.10669543565026	h
26.91512999843835	13.03182698594474	12.04695317051878	h
30.13065974921716	14.24561519787591	9.72482646857635	c
30.20953906187856	14.19510861278618	6.84691188411376	c
29.02793335908110	10.30218165099077	6.12667805903278	h
27.22204962964390	12.60042240500438	4.46684523823322	h
26.53152848077595	5.19032533154276	1.92809740145389	c
22.07448298818518	6.36829168448630	1.34073199613024	c
23.32050810110275	1.82565186138207	1.93476990059936	c
18.64220293733407	3.55409819714092	4.13425764912427	h
18.82909099037616	1.17066823537587	6.43909602023708	h
17.33667508662966	4.04823867022506	7.14289579481134	h
17.99223848060767	4.62079661218191	11.13141321049590	h
19.46058132647163	1.66394861483833	11.46353773664440	h
20.24700044562901	4.14972683538012	13.51568358041081	h
25.39956883659310	3.05293883460035	14.42938149648759	c
28.20366734754947	6.39711139333571	12.81516299350714	c
24.03949178968996	7.60517593680013	14.49797744505155	c
31.19898628390894	12.66038591680204	10.50727151260382	h
30.86772449577750	15.99088479298195	10.53600060057624	h
29.66069406295537	16.03520431118418	6.08432158816940	h
32.06209010834251	13.71237622216147	6.08553240896033	h
26.69126260429058	5.00231832462134	-0.12409294530986	h
27.04899853756639	7.12520830601619	2.43413143330246	h
27.90752280792970	3.92398012103033	2.80085104503195	h
20.08896360459752	6.09929571482225	1.81949012390347	h
22.55559632094369	8.33546954679919	1.76426355780325	h
22.27425071262509	6.11023230780660	-0.70210844646437	h
21.39302262531430	1.22556211223486	2.34160885500111	h
23.63075961151851	1.62450673499803	-0.10065679822383	h

24.60244286139307	0.53470126353038	2.91061067298309	h
26.28878317304900	3.27748765188452	16.28396696069738	h
23.49156046074081	2.33648645524277	14.72538480839125	h
26.45228178906018	1.61966125844075	13.38104917649811	h
29.35396071127991	4.98902526067080	11.83921634483741	h
28.39310692506484	8.17893616036872	11.78679815748032	h
28.97595119625008	6.65740287989036	14.71490192490558	h
24.22040315392846	9.45388106775259	13.58124771554922	h
22.02840747527411	7.21476115161482	14.71355999080836	h
24.85342610434011	7.78880576072360	16.39085306173579	h

2, RKS ground state

15.62687499577096	14.93287144847403	7.96882211975521	eu
11.29304859499026	18.92235682607550	8.61765161224360	as
11.38885622241825	12.38858496779061	6.68843973034886	o
13.57894082010729	19.64135670735578	5.95479449005882	c
15.94797855678518	20.30397830830410	6.90998492371373	c
16.17810621757181	20.19469640372501	9.61555211356771	c
13.98765693086011	19.48004121129897	10.90239344214245	c
22.14578288658508	9.66039695646080	7.99068676121728	eu
17.25936235517861	10.12058314376374	5.81582641655062	c
18.66088707891394	11.97072955963042	4.50692078083919	c
20.19911287327038	14.02066109408049	5.24097421211883	c
20.96289440954039	15.04490903855491	7.57664348111020	c
20.53222334425410	14.45821321572515	10.13347352762222	c
19.11717805608162	12.61683616394418	11.44021730324928	c
17.54651426898443	10.59356337480154	10.70691182133101	c
16.78283287316397	9.56694569803655	8.37141952629829	c
9.36853689683174	12.04198574463143	8.43038265849918	c
10.77474700728658	10.89467263753519	4.54015347256085	c
12.74162601663954	19.67883518464410	3.19216656325484	c
18.14254431619359	21.31455440122412	5.40286212127796	c
18.59857738487328	21.09320572751966	10.81187176211226	c
13.59099124527234	19.39533296280740	13.76146955410089	c
26.42798457572197	5.58323893334295	7.53653551587634	as
26.42927591339451	12.37580957218534	8.23016484630155	o
23.57395012043810	4.76980777509604	5.54340716816124	c
21.48069955391586	4.24801918313153	7.06477431153640	c
21.90407365819314	4.49080044422658	9.73782442733118	c
24.34156241541069	5.23751969474898	10.42506238478075	c
16.31166844486848	8.81945905221700	4.54617685452501	h
18.53566296016892	11.76428668455584	2.47068786802590	h
20.96420778644185	15.04704185894474	3.63828679084215	h
22.20836761112950	16.65793900569577	7.34623404152804	h
21.49046349382451	15.74918982281316	11.40605382161282	h
19.25379621579021	12.81392109487458	13.47596404044105	h
16.76621628639342	9.57979740161983	12.31006956971268	h
15.52826808916920	7.96091484366332	8.60107826461668	h
9.59651808130495	10.21417439178077	9.38855937525022	h
9.46774701274137	13.56982899918234	9.80950914172176	h
6.99610168721480	12.10502016111568	6.81253145297773	c
11.82728206561048	11.63464274740584	2.93070273918047	h
11.37633225420969	8.93876622876896	4.87246506887606	h
7.89923357860712	11.07065936494728	4.24783375029669	c
12.79788411362624	22.38329550742843	2.14276780150314	c
10.01494041534702	18.74585440747947	2.93596711781364	c
14.37268674957591	17.93136533813387	1.54758624984441	c

17.77536101649164	21.35843174378406	3.38528629602276	h
18.58728339842782	23.25191495410961	5.98288443579990	h
19.86194265704164	20.20287619351321	5.68888918229043	h
20.25604460655049	20.36530571096872	9.82003233546701	h
18.72690794515192	23.16084294389054	10.74449527949955	h
18.77367857056540	20.53958515802895	12.78136205565985	h
14.04881679512615	22.01393990375574	14.92395172711610	c
10.86421664836451	18.67001625366650	14.40116285970840	c
15.29177598205964	17.42790080694263	15.04200671862725	c
27.32056637908073	13.94541693495978	10.23446989007117	c
28.16539661555165	12.45833890979856	6.14664014399400	c
23.76008899370045	4.49557700303667	2.67476487009199	c
18.98213748791509	3.22511070087596	6.16614366077097	c
19.82119683569626	3.72139731387452	11.51975203261860	c
25.38228482162402	5.55435503519880	13.09885468401514	c
5.46865824361040	10.99140722843441	7.63335326670261	h
6.34054380678465	14.05051188263854	6.62514570550028	h
7.09083952646514	9.22254037397825	3.82376491828771	h
7.37956225819681	12.34321441123240	2.71314664587595	h
14.68397157456091	23.20954937293453	2.16302312619083	h
12.11401767906373	22.40973042606763	0.19023703607667	h
11.57020437966162	23.60052515179192	3.27067780607677	h
8.70634639447975	19.95835840463776	3.97455203048689	h
9.45058054951177	18.75115077019193	0.94730559569416	h
9.81094184679996	16.82371768582416	3.66370449577510	h
14.18322238197580	15.96299966413730	2.16257856628883	h
13.75472525915892	18.01184817894363	-0.42487849154149	h
16.37574413175431	18.40577277072502	1.59909830791278	h
12.80583500135016	23.40826189507883	14.04479306415708	h
13.63959913348586	21.97535308307306	16.95141251874424	h
15.98880767871366	22.66072925610095	14.68489147084583	h
10.38058058251356	16.80577320987045	13.65287422415408	h
10.61266081919145	18.61507695215753	16.45178909105284	h
9.52113909570806	20.03282297472408	13.62680967815086	h
14.83805861780933	15.52599126127307	14.36746303736767	h
17.29647954839651	17.74653522086699	14.69344190837403	h
14.99418234586844	17.44596214774648	17.08907715649798	h
26.30379966234465	15.75093120210071	10.19783746538114	h
26.91740917734597	13.00406632291317	12.02594777632749	h
30.11443605874723	14.31010398771699	9.72674846253116	c
30.16332466956766	14.40028949469825	6.84881472319371	c
28.95400937919553	10.56194518528540	5.91185855998461	h
27.12031810332954	12.96399931176555	4.43674065297651	h
26.43221606515158	5.11400836013415	1.75335243357021	c
21.96928306333125	6.30712230418666	1.29034175328665	c
23.22061968710927	1.75802291752887	1.87936901707593	c
18.68358657060405	3.49360249957211	4.15267151371703	h
18.84603737875515	1.19083437362881	6.53873853394796	h
17.39576900166698	4.11189407061377	7.14417797955618	h
18.05020299777624	4.70505312108615	11.11182310312578	h
19.42693195412595	1.69530142974817	11.34585489046339	h
20.27584188926418	4.08176333569886	13.48775056446281	h
25.32865697060515	3.03025426942681	14.52772250009200	c
28.14588434501611	6.40836989396704	13.02553870885751	c
23.92857552889806	7.57731958020781	14.58566243874966	c
31.18667463502265	12.68696077574865	10.42076103022993	h
30.86303803475556	16.01327499245843	10.61349364920541	h
29.62217615254898	16.27991680864114	6.18389852031931	h

32.00506193176690	13.94346068032695	6.04624978553329	h
26.53025448297241	4.92074765318115	-0.30213538856660	h
26.97153258680692	7.04879842253249	2.23784009860747	h
27.82769199883841	3.83961322927266	2.58372728280425	h
19.99627122400815	6.05616374735702	1.82554515239155	h
22.47637061110402	8.27284149811286	1.68833116043795	h
22.11105225847672	6.03389485914917	-0.75535690742597	h
21.30455867519557	1.15962161244866	2.33698749810465	h
23.48070920579580	1.54378873477388	-0.16176740065172	h
24.52642921828167	0.47296644821061	2.83095261596174	h
26.16767642338747	3.25112428501718	16.40598930998166	h
23.42224728416836	2.29097108300366	14.77035836239167	h
26.42285882976219	1.61515304460423	13.49747689139336	h
29.33846591266425	5.00984492404887	12.08612671568358	h
28.35373758065734	8.19619070686983	12.01068355655109	h
28.85698270251835	6.66900853153202	14.94882417134658	h
24.12725997367949	9.43132901597213	13.68370721218212	h
21.91416417734176	7.17835619871745	14.74114568852276	h
24.68780263671821	7.75311218905954	16.50187321158157	h

3, RKS ground state

15.52687838682809	14.92542602936436	7.83623141726634	sr
11.28915005480732	18.79627505108834	8.71104125163213	p
11.68714554096053	13.07841945426258	5.08701746096475	o
13.82659134208996	20.09808483840316	6.97706848062969	c
15.97901996758186	20.34994821715548	8.50972984389268	c
15.60871761372907	19.44710134197620	11.02709517733415	c
13.16553570182865	18.50126116418106	11.45266708821186	c
22.13585097723936	9.61086583654598	7.76643489116025	sr
16.84524745838790	9.65668610696303	6.59615149538568	c
17.94009410716943	11.05076956747196	4.61241224758506	c
19.50809672302951	13.20217002961782	4.46211226884623	c
20.68346167598547	14.78990381807404	6.24633769561614	c
20.84107944883057	14.84690873711827	8.90188686880861	c
19.79282938668200	13.41735796530286	10.89019865344490	c
18.12874526821438	11.34313255724266	11.03687355130959	c
16.88148104000558	9.80580023598172	9.25303493112955	c
9.24262057989267	12.57605585038513	6.10126468189625	c
11.64930678914276	12.10697199776443	2.58117110471773	c
13.52526350584863	20.99684791143188	4.24950758350566	c
18.41667947114106	21.64131174018362	7.82224548743380	c
17.64827115532461	19.81634783038314	12.97065772917181	c
12.06917542577701	17.58214228990300	13.96187841660745	c
26.19284327188475	5.46247365695844	7.70022982348778	p
26.29149313710746	12.09579195021658	6.31129544115574	o
23.30863698116192	4.27733357048934	6.51879289218700	c
21.47925786959788	4.16498284698706	8.43947493026439	c
22.37586004300174	5.06623090896019	10.82040114600370	c
24.89658700406224	5.89696076883745	10.74481715748746	c
15.67775671551770	8.11309969368617	5.91888216029603	h
17.43443183322052	10.31841690930279	2.76538632327905	h
19.90755893290737	13.75593013999103	2.52812031251135	h
21.77889799865240	16.28128736120211	5.36313877412666	h
22.02106718470925	16.37888145543486	9.58431772703838	h
20.35705076618983	14.10523448572258	12.73706917328316	h
17.71632768279872	10.80118994050135	12.97018834965897	h
15.73363495393832	8.35438485297611	10.13683388552913	h

9.15357223758561	10.59040060894132	6.69764872858727	h
8.98366915546624	13.80952077278268	7.73044408669408	h
7.41071317406424	13.11532825615172	3.94691055991331	c
13.18947216383247	12.98521533452115	1.53238970418895	h
11.97806589356285	10.06020629328692	2.63715815783753	h
9.02075721765251	12.72084694101846	1.55369406705825	c
13.92619195315586	23.86366430889935	4.07148563867434	c
10.85142355511443	20.46505275442882	3.28357788600068	c
15.35203477455473	19.62420739376601	2.46326056058530	c
18.61694366497193	21.95954734027726	5.80341363426199	h
18.55637988671087	23.48966061935947	8.74843994404737	h
20.05965522566359	20.54800238761398	8.43321319263846	h
19.50791905625055	19.30945485852648	12.23068560782306	h
17.76083586317550	21.80296038315120	13.55022368564896	h
17.34850385767850	18.70491589365308	14.67148132255046	h
12.17243649420699	19.70225980633017	15.93872557563822	c
9.28583054524583	16.85180841054208	13.66593109363870	c
13.44950623297242	15.23537360319722	14.95461670237320	c
27.47947476784731	14.15175531469070	7.59462037550138	c
27.75910378978205	11.38991353226767	4.13802821665405	c
23.05275735417524	3.28816185195365	3.81717253337500	c
18.89813637542308	2.99018558929954	8.25910116703815	c
20.72382808096505	4.83370849093563	13.12269329579508	c
26.48188446596708	6.84429216909673	12.96460091609994	c
5.76952884728105	11.87104775834873	4.01641375106876	h
6.73654234073681	15.05922306038418	4.06248761611097	h
8.31095439744538	11.19362598681288	0.36558753602222	h
9.06130725538003	14.43422855155333	0.40953197802447	h
15.82655824271456	24.43702281910736	4.61882471899282	h
13.60001696645872	24.51485366738154	2.13507719853924	h
12.59443594347959	24.84044919181665	5.30987941959905	h
9.43534486178247	21.45957365705723	4.40811420399178	h
10.67518245228035	21.09337000348405	1.32176312626176	h
10.41390390951596	18.44831619399685	3.36097648478468	h
14.91012061585896	17.60248911736021	2.38526095693462	h
15.17375594721641	20.35745169803644	0.53701913864341	h
17.32461007723791	19.81310220144447	3.02807705728192	h
11.15533225817594	21.36285167522324	15.25332117456979	h
11.28087788440840	19.08288628438360	17.69951882850958	h
14.09903189163009	20.28166235731530	16.37734311087143	h
9.04205434273258	15.32155022310632	12.29941367266167	h
8.53442044288052	16.20740801810528	15.48033063034890	h
8.14888263313521	18.45154502888303	13.02820473994026	h
13.20371307818612	13.64080866133272	13.65917620710277	h
15.47363807872344	15.53455784058021	15.19169759366301	h
12.67503419674590	14.66455006627101	16.78582460744865	h
26.51468787199939	15.91724576432446	7.09927864211958	h
27.28609649451956	13.85075142494262	9.62700869248884	h
30.19327936919133	14.15179399501366	6.68278436558503	c
29.87581774324560	13.32188762192969	3.94374120963684	c
28.46586519250220	9.47376776909909	4.45431285006842	h
26.52172131151507	11.38380539749555	2.48296796275615	h
25.53497946204970	3.60167810510958	2.36516663068474	c
21.02183953937260	4.72150729497255	2.32632894795115	c
22.44574228930314	0.45217723070101	3.83731259447666	c
18.22894304314694	2.81270448352761	6.32480523072857	h
18.89426234990799	1.08858245940618	9.08348998522655	h
17.48937756608646	4.08703906459943	9.29534077404337	h

18.84722789524241	5.64041982854839	12.80933185901217	h
20.43786574214554	2.84272917787862	13.61887986244964	h
21.51302652789287	5.76056778216753	14.77557189704209	h
26.76003689475854	4.76716997409300	14.96947328664921	c
29.15328715625444	7.53439416074149	12.09523223978911	c
25.34602108981368	9.22947527674783	14.16073230596663	c
31.30432572281001	12.76549843718989	7.73633119970036	h
31.09235750784144	15.99547670716315	6.88278550174101	h
29.30167032444080	14.92416175759361	2.77274326698138	h
31.58793027549751	12.51465429491422	3.12990835723132	h
25.31444755239163	2.88730743294642	0.43798359230380	h
26.10273285053306	5.58456522024562	2.24959519458352	h
27.07149969227147	2.56031161846693	3.26705660866232	h
19.17395612604490	4.66926595274268	3.23565273640561	h
21.54935748768505	6.70959725158856	2.09936063252923	h
20.81841867460512	3.91985460722877	0.43000583646605	h
20.63505085777933	0.03736631699191	4.72560281668386	h
22.38587557505243	-0.28093593196132	1.90378267426909	h
23.90573168954047	-0.57901598857254	4.87013514006219	h
27.97989456857060	5.41293367655825	16.51049813404615	h
24.95418533943092	4.20653983214621	15.78537218564461	h
27.61812794304918	3.08751984157630	14.13077214919842	h
30.13353749299124	5.89852860947158	11.30679974693849	h
29.11737049517674	9.00158612580526	10.64088740974096	h
30.25470219809560	8.23670953653520	13.69739502319771	h
25.34906385747510	10.80073799243491	12.81141714871861	h
23.40575211601107	8.96646899248690	14.80010853972212	h
26.47147762122190	9.82970415057999	15.78920868875558	h

3, UKS singlet excited state

15.59612696633185	15.00218997226721	7.77715531359448	sr
11.31121812671289	18.80804798242855	8.74305070413695	p
11.69981942167455	13.09120978298800	5.11872644097460	o
13.80313339722065	20.15140847256449	6.97658920827837	c
15.97355420391279	20.43052082855122	8.47878766156107	c
15.65162597989424	19.51943686170537	10.99990278514303	c
13.23091011307207	18.53209485931269	11.45655832252952	c
22.30765039409749	9.76183760181298	7.56721917155024	sr
16.97077673210441	9.78823429894930	6.43924132928469	c
18.00826193779725	11.24955880269578	4.47298488860801	c
19.55452791082964	13.41552908404033	4.34065599489912	c
20.75042384176447	14.97330427957577	6.13792252898438	c
20.91211606622716	14.99979539711924	8.79524477643565	c
19.88616697829579	13.53010470029967	10.76397050616695	c
18.26710537322966	11.41884687587836	10.89378481786932	c
17.03993429650778	9.87923587951829	9.09779543448962	c
9.25880549170653	12.60911301675531	6.15061197875575	c
11.66355380712383	12.03782678873511	2.64702240052231	c
13.45536407058781	21.03791007879534	4.25034491652914	c
18.38267820717639	21.75502921751109	7.75582528356142	c
17.71164978822383	19.91892185059515	12.91612394293186	c
12.18408519272471	17.58396778956315	13.97641040355564	c
25.92745428302258	5.08094835692320	7.95251044117145	p
26.58696118348063	11.92210143102857	6.11285341694316	o
22.86099069606038	4.70301828236452	6.36932221047488	c
21.31039119111305	4.11244275507626	8.46145166161470	c
22.18393739071085	5.01351327171675	10.87900116284903	c

24.49085219353077	6.34652528408017	10.84602342231955	c
15.82478655010034	8.23850734181577	5.74043690013549	h
17.48690352858114	10.54805626594709	2.61817820311999	h
19.92241426797689	14.00800220897204	2.41209394233354	h
21.80784912843457	16.50151565598041	5.27060133225634	h
22.07008676805152	16.54129591007184	9.49453397799923	h
20.44815178327466	14.19972217296276	12.61818584124433	h
17.88461159199480	10.84171316816101	12.82298348737381	h
15.92814670806367	8.39071519259504	9.96674635672677	h
9.18502599322678	10.64689847393872	6.82246547248897	h
8.99240041132267	13.90204941680605	7.73159005753991	h
7.41831959309671	13.04893728130275	3.97777447281461	c
13.21295556842809	12.87008000001506	1.57542226112232	h
11.97721192863375	9.99117126113539	2.77025802180621	h
9.04286289827335	12.63678259489766	1.59707370620232	c
13.86798929720624	23.90084137592974	4.04396037970733	c
10.76160612351558	20.51326760493389	3.33753210566999	c
15.24219579749816	19.64181848066736	2.44161075618943	c
18.59907283719977	21.98244532441887	5.72563839281689	h
18.46421653030675	23.64839005627990	8.59477559487225	h
20.04485967148179	20.73508712656093	8.43495573394757	h
19.56051866578947	19.37061734970673	12.17787077109466	h
17.85014475730959	21.91955446915834	13.43890276970043	h
17.41328332125068	18.85853966064273	14.64902629048320	h
12.24567076716259	19.70832113663419	15.95081533447067	c
9.41919085182288	16.77670026771779	13.70672525834643	c
13.63431156169669	15.27413977884100	14.95711384533954	c
27.57079800654189	14.21585133495342	7.15024651269120	c
28.36751877260448	10.87340561236399	4.34690420538597	c
22.75933179967349	3.52237557426335	3.74377320983614	c
18.94678084373858	2.55560967802409	8.38454124317196	c
20.65969661862349	4.39143200110749	13.18269848547327	c
26.15929948798640	7.01886331466551	13.09216072869395	c
5.81132439104762	11.76230013032109	4.07523824918140	h
6.68906072905007	14.97509375885846	4.04199754747933	h
8.34242093590392	11.10142600147215	0.41376519068892	h
9.09171217239774	14.34305323958372	0.44226265959890	h
15.78315198509699	24.46714506656540	4.54449612788543	h
13.50276055041591	24.54007874345217	2.11046605281437	h
12.56908795069579	24.89382823578532	5.30434760449039	h
9.37207021713667	21.52269812879607	4.48167558592625	h
10.55236942846391	21.12872732075511	1.37481623019612	h
10.31527624327179	18.49937312262902	3.43681392445473	h
14.78416455286631	17.62286779834690	2.38412395934596	h
15.03670191498882	20.36273390040182	0.51331137016603	h
17.22572955267940	19.82068031825030	2.97202202857338	h
11.16392855412281	21.33287693337240	15.27786879465514	h
11.40067603922172	19.06219399319287	17.72510947941812	h
14.15796722857010	20.35333458717918	16.36018733422830	h
9.20675085914691	15.23694986171625	12.34558945309768	h
8.70204858360773	16.11581480191977	15.52916731768290	h
8.23267497074310	18.34275384086207	13.07578854305874	h
13.42330412317085	13.67597518794896	13.66023829887579	h
15.65144469156477	15.62495372781089	15.18178588763245	h
12.88591752588875	14.68045999630910	16.79209268948521	h
26.75309466261569	15.83027514728674	6.14150583334094	h
26.99751457149485	14.32459604092953	9.12871824071309	h
30.40139021495970	14.06001489590542	6.76248147400747	c

30.55708224397880	12.73046085404242	4.21266797988971	c
28.93497766088099	9.01772638839340	5.06279184335119	h
27.42439629972015	10.61801710348225	2.52761958332789	h
24.98028455100727	4.51458857148001	2.17672341051274	c
20.34920219908154	4.25082797329769	2.30925285803459	c
22.99847455780349	0.63080912110806	3.86871257460684	c
18.39777775287262	2.04158915244145	6.47733592892307	h
19.17609176611162	0.80370313492004	9.46141133653879	h
17.36491024850594	3.57452260129603	9.24566616347607	h
18.77597792110149	5.24464967457641	13.07235966333440	h
20.37584148808708	2.35093765081525	13.35904074902086	h
21.53903181062162	5.04806089781066	14.91433640234371	h
26.90683707035459	4.65819198704527	14.61041376293405	c
28.60070113438998	8.23127260276881	12.11911833880090	c
24.89664814795287	8.95721689671489	14.83706378597142	c
31.26404394204966	12.90388201527946	8.24069612794155	h
31.30334515537276	15.91294648739806	6.77452474197694	h
30.26900942990168	14.08650215051622	2.68114697834444	h
32.35768778771445	11.78139986019200	3.89326167286281	h
24.90600233320297	3.74824857161185	0.25895893739362	h
24.91696539159247	6.57716560468315	2.03953369099161	h
26.79387985582498	3.97623844742594	3.00796788811522	h
18.62604680449644	3.60989389803697	3.23737513509273	h
20.21778516939451	6.30325315170910	2.11104520792299	h
20.38390318091935	3.41674105381348	0.41645903840688	h
21.38758791355596	-0.23794642334957	4.81839853255236	h
23.12054441962067	-0.17399864876868	1.96743882871416	h
24.70283014562840	0.10120086558170	4.90962442133784	h
28.26820274186568	5.14448804984521	16.08957524678661	h
25.28249130844212	3.76991104989987	15.51781339298311	h
27.77438208240090	3.25750347700237	13.36368247295986	h
29.69993208617094	6.90012478878504	10.98288534790652	h
28.18258172420629	9.88172678998288	10.94640840575518	h
29.78060414671351	8.84266030903895	13.70202368309573	h
24.48564520148927	10.70270107613665	13.80801730962289	h
23.12178355848424	8.28792007473341	15.63926147865103	h
26.16021279357269	9.41681465468589	16.40931933661131	h

4, RKS ground state

15.49885659634301	14.89444006645013	7.82460611446494	sr
11.02192324485822	18.78246245154122	8.66788297919618	as
11.71897100855193	12.95493766751623	5.06421692888110	o
13.82695516081174	20.12686750168604	6.89438328542686	c
15.92486915800650	20.33825142521399	8.48171839941960	c
15.55401003818206	19.43532518168725	11.01770768500705	c
13.14356286846570	18.49187321077615	11.53265404780486	c
22.14779214496495	9.61956591502047	7.73659215517872	sr
16.85437775553317	9.63117118871529	6.57555586883862	c
17.93666437741343	11.03445480176041	4.59211878692520	c
19.48675171798746	13.19899502317501	4.44274762705481	c
20.65190137004741	14.79240415330242	6.22780007865150	c
20.81414636620483	14.84539122070763	8.88282453466537	c
19.77976645340922	13.40585655683561	10.87142434421609	c
18.12868687529621	11.32176066932667	11.01755405745981	c
16.89065958332049	9.77855585801291	9.23223492643883	c
9.29308626159028	12.34842432214994	6.06166435659445	c

11.68505728097633	12.12750377555897	2.50671340599488	c
13.60886290367044	21.04427919639152	4.16603872520452	c
18.38943025688859	21.60960234559380	7.83712419638311	c
17.63744981801806	19.81429090984089	12.91870252433957	c
12.13099735286029	17.58665846408660	14.08047038766397	c
26.45801167952321	5.46463489307755	7.61433539619534	as
26.23070134431953	12.20372550846233	6.24715460817046	o
23.31308123438069	4.22453627635147	6.44833543829981	c
21.54321654160364	4.15706690921918	8.40623340206336	c
22.43493640313540	5.07297648522005	10.80301380189582	c
24.93502761833467	5.92177182276520	10.82184159939399	c
15.69394974924475	8.08248694129415	5.89776226474628	h
17.43282673389677	10.30160748155416	2.74483598805535	h
19.87959711304921	13.75848877791347	2.50892155533909	h
21.73837708081680	16.29093161448856	5.34551038266943	h
21.98850349866941	16.38162716738286	9.56544424010507	h
20.34347216986988	14.09441807567250	12.71818142432358	h
17.72220116766799	10.77426063897006	12.95057544928728	h
15.75011350482973	8.32064621004273	10.11495326123208	h
9.23208868020322	10.32435701689935	6.51732006578899	h
9.03009759192197	13.45953822089234	7.77667194000326	h
7.44655066670008	13.02089140723959	3.96346003837768	c
13.17488130342009	13.12741834081723	1.49427398015752	h
12.09936772524792	10.09691022081869	2.44159358014740	h
9.01829487940886	12.68717664706501	1.53651993741190	c
13.99058997757004	23.91676995149415	4.01710353201111	c
10.97005752201799	20.50473324067199	3.11027414696710	c
15.48950837070566	19.68784691001428	2.42223617966271	c
18.56877609384252	22.04712947462994	5.84038541880996	h
18.58393016325827	23.39310255319462	8.87306129410195	h
20.01518969581985	20.44501307889152	8.35583272581618	h
19.47173715060737	19.23033122868270	12.17314470986883	h
17.81224343844003	21.81647803185985	13.42394494342011	h
17.33205827471783	18.77391103702676	14.66140724007309	h
12.26332605242641	19.72272314458726	16.04013193972146	c
9.34601329751222	16.83000422891523	13.87405634544066	c
13.55189235888503	15.25108430688736	15.04565249438964	c
27.46319491449617	14.17398159479542	7.61710457575883	c
27.63559720413945	11.62167774784277	3.99894681290426	c
22.98302680952080	3.20875706605073	3.76591925556443	c
18.94808002630948	2.99705486549296	8.26793046087041	c
20.73000716450866	4.83136138435542	13.07072304024660	c
26.43808021391354	6.86604225160509	13.09841857761950	c
5.77019679544982	11.82274454083278	4.00168008942667	h
6.83626692079907	14.97841481581884	4.17561073272603	h
8.32388080656557	11.14612605109954	0.35677519208602	h
8.98779915739708	14.40457232577410	0.39825552727258	h
15.86577215977492	24.50811778678779	4.62762611470486	h
13.71805179482504	24.57634252526778	2.07530393250560	h
12.61056913281200	24.87215180344661	5.21881025483904	h
9.51328038964458	21.48749117018732	4.19320441345351	h
10.85029275749016	21.14368568940704	1.14789738355209	h
10.54312279158802	18.48408315458557	3.15955017254013	h
15.05916884202253	17.66450951134005	2.32943162037558	h
15.35180696813360	20.42556835712687	0.49445536133673	h
17.44714365582727	19.88086273899587	3.03301238569391	h
11.20921914348394	21.36552349611150	15.36771277723497	h
11.42098495426394	19.10549289954561	17.82568848184749	h

14.19143528810151	20.33037576601811	16.42910315661161	h
9.07567769787983	15.29384551400122	12.51875277643896	h
8.65476305886348	16.18356434729233	15.71134401244847	h
8.17573652222154	18.42052096069173	13.27288986241432	h
13.27133731880860	13.65149533959305	13.76386772693665	h
15.58129781567076	15.55452652575098	15.22113630116571	h
12.83187971885300	14.68646423334974	16.90081954544365	h
26.51635970451655	15.97640233928273	7.22920237422580	h
27.29443124930852	13.76876820461224	9.63348153528376	h
30.16311596875021	14.18456060070691	6.66566664955709	c
29.79178598353846	13.51878412545523	3.88822768779508	c
28.30922086920053	9.67474027482366	4.17063050445878	h
26.36459780269201	11.75444506688919	2.37480789355077	h
25.42882165713560	3.49562814885542	2.24714961558275	c
20.93070524222562	4.64581049028805	2.30593399069123	c
22.37161299270405	0.37291283353378	3.82271805618491	c
18.28417993245473	2.74465028074270	6.34159457241178	h
18.92848952678354	1.13149706991867	9.17045050494504	h
17.54376288697852	4.14493689162475	9.25287935590280	h
18.89247081185770	5.72363313287603	12.75780284519301	h
20.35664020103073	2.83783739283251	13.49060880852211	h
21.52811918725220	5.66646073640325	14.76607539441256	h
26.70335027737945	4.76678486564539	15.08384549301419	c
29.12294857063358	7.61052685370079	12.31818922091680	c
25.23769384176505	9.22474720227858	14.28685518421162	c
31.26445598845162	12.72205872425981	7.62196984765424	h
31.09621167019669	15.99928322719980	6.95606201691387	h
29.23722000383959	15.19705900686648	2.81856939631829	h
31.47659401398530	12.72546890190882	3.00606129558717	h
25.15700453288445	2.76977443134010	0.33103577510854	h
26.00296130830749	5.47522356575062	2.10265302044671	h
26.98010487387965	2.44667458330177	3.11541672553825	h
19.10062001185002	4.62154329106935	3.25076594887530	h
21.47474051904034	6.62603264012814	2.05276431503951	h
20.68340983215629	3.83042743190275	0.42075996637098	h
20.58336871491857	-0.03883652342794	4.75575832707650	h
22.26829891683430	-0.37386652664239	1.89631855519786	h
23.85640919558787	-0.65001601342285	4.82814874550044	h
27.87266810756341	5.41474758721810	16.66256974889916	h
24.89116629908130	4.15711526974202	15.84788133177421	h
27.61536350877171	3.11668626415151	14.24320637275780	h
30.15436299370134	5.99854507014848	11.54465934995655	h
29.10580104872771	9.09597561413232	10.88166613672964	h
30.16657622342706	8.31162353260232	13.95884080085881	h
25.25373415316754	10.80503017650814	12.94852728414291	h
23.28584490452682	8.93472211038172	14.87556133504695	h
26.31624068720155	9.82611422107935	15.94629746391725	h

4, UKS singlet excited state

15.60388644062135	14.95914041136440	7.77231488383636	sr
11.06368031759889	18.75701909273980	8.71359666462004	as
11.79690936055996	12.99970824213085	5.02881624626455	o
13.81396282399200	20.15723521135309	6.89843048475557	c
15.93153457456047	20.40903780194702	8.45322364145282	c
15.61644620199496	19.50092472669620	10.99461595711734	c
13.23501479263461	18.50640340098407	11.54474948156599	c
22.32843503246406	9.72668600311405	7.57839960925528	sr

16.99994121889141	9.75752416760608	6.42639466932613	c
18.04637206611630	11.22480794048663	4.46965785702433	c
19.58695420101425	13.39613655034731	4.35065497212320	c
20.76801535174468	14.95246110481299	6.15820733238282	c
20.91883840267056	14.96890541532311	8.81595221923849	c
19.88819301493484	13.49011443257678	10.77596769395655	c
18.27140061375090	11.37666297361801	10.89192850444162	c
17.05532086512558	9.84071325856607	9.08525622948543	c
9.40045246572191	12.33488379879862	6.05712742498531	c
11.76598478315243	12.13695748309699	2.48356866052941	c
13.54207071320729	21.06060350633026	4.17062846652490	c
18.36373839745035	21.72270061429122	7.77140694553079	c
17.72069307634668	19.92654148879769	12.86273793158957	c
12.28006710871004	17.57934045109145	14.10664350421047	c
26.18885075432860	5.02338596503870	7.91965604448215	as
26.54406533065918	11.98227314396133	6.06806373809183	o
22.80863802265391	4.69525752581423	6.31827390556359	c
21.36337615105953	4.06752047062119	8.45886311424508	c
22.22168168394597	4.98408699410591	10.89178649757926	c
24.46888862289514	6.39121413221932	10.95345561793942	c
15.85631308073999	8.21051018009519	5.71729338492648	h
17.52751186628139	10.53377136552725	2.61023075587375	h
19.96219477673158	13.99554030471521	2.42555137796949	h
21.82942380791041	16.48336085312049	5.30023852913242	h
22.07069830723409	16.51012662294697	9.52602619106293	h
20.43768624981815	14.15747440632638	12.63503784732604	h
17.88291655670654	10.79101005678632	12.81733286969157	h
15.94302712119901	8.34692781751294	9.94467505707431	h
9.40196520055466	10.31505634673993	6.53736540315540	h
9.12250808527838	13.45896036674010	7.76092117226635	h
7.51619861287594	12.92546840954844	3.96805329730858	c
13.21279187824452	13.17087276696064	1.44386027143537	h
12.24578483420499	10.12030212045513	2.44034732613621	h
9.07321054201378	12.59611873016765	1.53149669914135	c
13.88754743771604	23.93622260147125	4.00270903858829	c
10.89455284493002	20.48391805628316	3.15709448031044	c
15.41463597971661	19.72073883993066	2.40528263885426	c
18.52003892917303	22.13502846071632	5.76711874049925	h
18.52929282991347	23.52445623352451	8.78091537143337	h
20.01692300083996	20.59925460493109	8.29391888791996	h
19.54795262562279	19.32536633790587	12.11256597249255	h
17.89917185276734	21.94082651893880	13.31605939459837	h
17.43521393015336	18.92949246489390	14.63356068630484	h
12.36422624524007	19.72948979674985	16.05351600861775	c
9.51950228669352	16.73008979376279	13.93391465617595	c
13.78563966655157	15.29687426385498	15.07060139494096	c
27.57684446176883	14.22259371383645	7.16737432670872	c
28.22337867679245	11.04466893415772	4.14874744731776	c
22.63162934806394	3.51970910716459	3.69571854550778	c
19.00332189043064	2.49192482844583	8.41686095336479	c
20.66402033891306	4.31828166637886	13.16719111691186	c
26.04426512838443	7.07717842852764	13.26170850954208	c
5.87288855425570	11.68429443954572	4.04110820467089	h
6.85550397429219	14.86916254903153	4.15620811205598	h
8.41535398026063	11.01164839653252	0.38881302927076	h
8.97760651631181	14.28929218235190	0.36138040978422	h
15.76461640214144	24.55081210941754	4.58360175695331	h
13.58011253041964	24.58282536004148	2.06164949440195	h

12.51419226569784	24.88118977590616	5.22023301869125	h
9.44285340439477	21.45523108294084	4.25704615473791	h
10.73857985515516	21.11114011099254	1.19335351329731	h
10.49185644635396	18.45886478904858	3.22301328728522	h
15.01314020836275	17.69074940052077	2.32951122346438	h
15.23726802207847	20.44568379265077	0.47581792398054	h
17.37837299352999	19.94643919825667	2.98519119414276	h
11.24269421944014	21.32982946853744	15.38782329688683	h
11.57243888905112	19.09284477808342	17.85548237235128	h
14.27582139672784	20.40727203631801	16.40795490915674	h
9.28611422052257	15.17989029124860	12.58775236682112	h
8.87040190213903	16.06889685784611	15.78144023933834	h
8.29011349398079	18.27775335660335	13.33890439156037	h
13.55247361469135	13.68366900575867	13.79698080890024	h
15.80462208212796	15.66892287006570	15.23192388631407	h
13.09642883177163	14.71790206477516	16.93310553398524	h
26.73689931600287	15.88511892814441	6.25924929657869	h
27.07405219946585	14.24829703682837	9.16752379541908	h
30.38968951441568	14.06564928509108	6.67018391226335	c
30.43830145579928	12.87694803594568	4.04685660568678	c
28.79112512052544	9.13445509086227	4.70437924965415	h
27.19608427418293	10.93774463466979	2.35976938793786	h
24.74692743295108	4.59332947164064	2.03646305877195	c
20.14042472448891	4.19317924932352	2.37262147373772	c
22.96916205693390	0.63604709807771	3.77597360312624	c
18.55977521405442	1.78472028969019	6.54562575881614	h
19.17277565524345	0.85960361712106	9.67403109376568	h
17.37266397467346	3.58209935959981	9.07999333521425	h
18.73294582938950	5.03921356802555	12.98329705880738	h
20.51204933650291	2.26777628258230	13.38743595346508	h
21.44833100443999	5.06872861049270	14.90542559381516	h
26.79472353949073	4.72706372125146	14.79396132002234	c
28.49069459288166	8.35325545908467	12.38478974365280	c
24.68537407200251	8.98558748784864	14.97034151897610	c
31.29021334396238	12.82281761431545	8.05249583204398	h
31.31113375728502	15.90744264903475	6.74862142428863	h
30.12331234535372	14.32134173807385	2.60418301507189	h
32.21212353228852	11.92183854149659	3.61506926006810	h
24.59864449125921	3.85699699089424	0.11085085603634	h
24.63089119720320	6.65598501586676	1.94178878400250	h
26.61286386903976	4.08508991220590	2.76570529797040	h
18.47587612602758	3.49827950209809	3.36537602002972	h
19.94675662810659	6.24281160068817	2.20054787234384	h
20.11781806339802	3.37852317451806	0.47163530292989	h
21.43512890457875	-0.29925926742130	4.78668820370132	h
23.03350356510778	-0.14401184621809	1.86142857267100	h
24.73540566176638	0.15103022590513	4.73262490679964	h
28.10111386896540	5.23594927843862	16.31448040132628	h
25.16419444275594	3.79981304322233	15.64849657384835	h
27.73052744052385	3.34950050577807	13.57076307106612	h
29.66882020787176	7.05184881570803	11.29345054602440	h
28.07889789044505	9.99166945802141	11.19330268464246	h
29.59469457513486	8.99304870744820	14.01073815377326	h
24.26629776605008	10.72280927439081	13.93154709940899	h
22.90389207349954	8.27960825331774	15.72315492861697	h
25.89316729262348	9.47141103468388	16.57765597513986	h

[{(Cp*)(thf)$_2$Eu}$_2${μ-η^8:η^8-C$_8$H$_8$}], RKS ground state

0.87235262151048	16.44834424195042	8.67520086739433	eu
0.54647508770957	17.73188382672182	13.70727516536867	o
-4.33382750131198	16.25260263577920	9.36798918318569	o
1.42820863016205	11.65299721499674	11.11772390694402	c
-0.12638344284183	11.18584300081784	8.98898766106081	c
1.36246525154363	11.47592995884852	6.78700815734165	c
3.84158823549864	12.11873081670056	7.55215150488664	c
3.88192787744867	12.24483092590884	10.22476468442523	c
5.19874026511897	19.12163773736997	6.73521308482805	c
4.01107360555806	18.13415622042913	4.56963242153060	c
1.54304784289535	18.20843789827841	3.57991119671219	c
-0.72216166034650	19.41478822359831	4.28601189156548	c
-1.43346123491708	21.11264895426574	6.20408321450850	c
-0.20344875350213	22.21203860962915	8.29119211413566	c
2.22780079616372	22.03325707120496	9.34780856662823	c
4.45336642513207	20.72101543668631	8.72488925470649	c
2.82150378228019	17.59116115934333	15.16898300917975	c
-1.10110489260762	19.60314304709295	14.75246577793122	c
-6.17561206348380	16.73372262518268	7.47215232333492	c
-5.70295065120264	15.87659108629599	11.64611988198988	c
0.64265188734079	11.27522012299636	13.81093722661273	c
-2.75402424107094	10.15279629059385	9.06889187359745	c
0.52286379656072	10.92037750121299	4.14601255119214	c
6.13157720167486	12.21932079437558	5.90383245129364	c
6.22613873467570	12.64012371458304	11.75855707749276	c
2.86681260382409	23.45688765497172	3.83242670180318	eu
7.15003687410525	18.51185348396234	6.90267373662093	h
5.26042182614364	16.94369182840699	3.46478652522002	h
1.34291123225651	17.09792465946664	1.86882720483211	h
-2.24876981743149	19.03088295649538	2.97101363592390	h
-3.38522430586550	21.71931507576337	6.03112038903658	h
-1.43943050638818	23.46192130205913	9.35384058852472	h
2.43626054598478	23.16874230006939	11.04534279559979	h
5.96893439185074	21.05727602670527	10.07057655997633	h
2.29610633048545	19.04802616103898	17.58131919019263	c
4.35928142849310	18.45550175325672	14.08384575080782	h
3.26561152820677	15.60457380919460	15.47946886936992	h
0.46462479839430	21.06734359133783	16.65671568361671	c
-2.68797672511090	18.64696895894842	15.67965643103543	h
-1.82431439539243	20.76356179255600	13.20799956325218	h
-8.31922609887675	14.89899407362407	8.03235463147709	c
-5.28206434748120	16.46834783612821	5.63724795391730	h
-6.81396993935358	18.70304093261376	7.61285342667307	h
-8.15259541145289	14.52049630545399	10.91016250892880	c
-6.11565677637902	17.71950221724754	12.50530403183937	h
-4.50309505668673	14.80249169301136	12.93120325440631	h
-0.46466676680590	9.54175905966308	14.02631203774570	h
2.28371092650868	11.07971965737086	15.04986782126667	h
-0.50596642052192	12.82246906592073	14.57630037897825	h
-2.75893975618060	8.07891222426238	9.00715474263174	h
-3.74865951929254	10.69774883904204	10.79397012444622	h
-3.89507119120338	10.79185788393284	7.46941571425035	h
0.77848698622526	8.92163030422304	3.65592328671075	h
-1.48074554146911	11.34565322014297	3.86562480070034	h
1.57738561927402	12.02177061252287	2.75327569499806	h
7.19637262251192	10.44092674323889	5.99433072050198	h

5.63948143652023	12.51853590185355	3.92263958975805	h
7.43583594441097	13.72543901787321	6.45514672148316	h
7.69557015041531	11.27327017567723	11.24638689640375	h
7.06413599415849	14.51938333373744	11.50350207060864	h
5.87053826181885	12.40363338706551	13.77797268751532	h
8.09558615243452	24.04917034097478	3.95378373385212	o
3.55009133173646	27.91460085425550	6.64915812335302	o
0.94168650484803	23.27307140076242	-1.19714665056291	c
-0.68326716297869	24.95011520631700	0.10513980077255	c
0.69563927253966	27.17484941357093	0.65845071438535	c
3.17799525250068	26.87233383040040	-0.29620146735207	c
3.32536748868699	24.45715963656917	-1.44341364983295	c
4.01322622504348	19.81646306378254	18.42326487819172	h
1.38315183377396	17.82909979847450	18.97812142415938	h
1.49182317867122	22.59454376581157	15.71929273543066	h
-0.68687688641910	21.88588387019955	18.15775357243832	h
-8.00912499457505	13.11450087066518	7.04947729285601	h
-10.14309043929032	15.64423141358270	7.42748823250107	h
-8.07373060177986	12.51855371451986	11.38752400633807	h
-9.76841375821593	15.33473124900838	11.89800020429050	h
9.80782438416410	23.00340433494591	2.16663282354817	c
9.53998776901837	24.54052392919043	6.16651927539769	c
5.58752132630790	29.65621824906715	6.74326790194689	c
1.77951150604186	28.76649987404686	8.48041371165510	c
0.19941108081485	20.83895702005552	-2.42022300974957	c
-3.45244618385192	24.59243637871196	0.53408279216170	c
-0.40977313807369	29.51889153302114	1.78336340638693	c
5.19738186536932	28.84972464719262	-0.37055443996392	c
5.46277491564215	23.49016089763570	-3.02125292273937	c
11.50346831718777	21.24532417238022	3.68417356169958	c
10.90360068232174	24.53209645509231	1.28584134972932	h
8.71091461951758	22.05789760316454	0.70680580120208	h
11.53082144080984	22.44653866681557	6.33034726083395	c
8.23936625314620	24.57416072699698	7.76217418968839	h
10.44171788132862	26.40066406072866	5.98924198264273	h
4.36798018010820	32.24784913324336	7.05430207514169	c
6.68745526097396	29.43990640700135	5.01692240763915	h
6.79835684725030	29.21391602927328	8.37196469489173	h
1.82971593172600	31.65391624269080	8.34675819297739	c
2.35875614731675	28.09245523119911	10.35502503871197	h
-0.04785401419677	27.93388211827380	8.02736587330942	h
-0.44543349467387	21.13299628146633	-4.36955981305095	h
1.77363593504183	19.50408961319912	-2.50680427633259	h
-1.34153176664877	19.90383458670789	-1.41405733113187	h
-4.57653269230123	25.41129516717271	-1.00503467745150	h
-3.95932045059383	22.59473654941685	0.65036528100545	h
-4.09489266893077	25.48978079641793	2.28209699136145	h
-1.45769650877803	30.62549082886389	0.37689175640294	h
-1.74534801760782	29.10113054565055	3.30817398626116	h
1.04277261032253	30.76059691805535	2.56106032712566	h
5.34062549173842	29.73734569654375	-2.23884463119459	h
4.81994519107624	30.37155773382101	0.97330282531181	h
7.07091330120477	28.08991199162776	0.07137355525616	h
5.06172004180322	23.69574573109246	-5.04555662206886	h
7.21237896611169	24.52430559002821	-2.66037659175765	h
5.84442963066306	21.47893490588680	-2.71078517090964	h
13.38441157051360	21.08883645873512	2.85629166073510	h
10.68153128623261	19.35679144010126	3.75268122660342	h

13.37819379651589	23.22432861950774	6.81290794980096	h
11.03991744645710	21.06299547169177	7.77556263854445	h
4.06267162588351	33.12924597803779	5.21601560179720	h
5.55313561253030	33.51633441516488	8.16595275356233	h
0.25405886485562	32.36626182043642	7.22735439659661	h
1.69111173420684	32.48857133815278	10.22651964402054	h

References

(1) Watson, P. L.; Tulip, T. H.; Williams, I. Defluorination of perfluoroolefins by divalent lanthanoid reagents: activating carbon-fluorine bonds. *Organometallics* **1990**, *9*, 1999-2009.

(2) Zhou, Z.; Greenough, J.; Wei, Z.; Petrukhina, M. A. The dinuclear scandium(III) cyclooctatetraenyl chloride complex di-μ-chlorido-bis[(η^8-cyclooctatetraene)(tetrahydrofuran-κO)scandium(III)]. *Acta Crystallogr. C* **2017**, *73*, 420-423.

(3) Turcitu, D.; Nief, F.; Ricard, L. Structure and Reactivity of Homoleptic Samarium(II) and Thulium(II) Phospholyl Complexes. *Chem. Eur. J.* **2003**, *9*, 4916-4923.

(4) Schwarz, N.; Krätschmer, F.; Suryadevara, N.; Schlittenhardt, S.; Ruben, M.; Roesky, P. W. Synthesis, Structural Characterization, and Magnetic Properties of Lanthanide Arsolyl Sandwich Complexes. *Inorg. Chem.* **2024**.

(5) Evans, W. J.; Shreeve, J. L.; Ziller, J. W. Synthesis and structure of inverse cyclooctatetraenyl sandwich complexes of Europium(II): $[(C_5Me_5)(THF)_2Eu]_2(\mu\text{-}C_8H_8)$ and $[(THF)_3K(\mu\text{-}C_8H_8)]_2Eu$. *Polyhedron* **1995**, *14*, 2945-2951.

(6) Sheldrick, G. SHELXT - Integrated space-group and crystal-structure determination. *Acta Crystallogr. A* **2015**, *71*, 3-8.

(7) Dolomanov, O. V.; Bourhis, L. J.; Gildea, R. J.; Howard, J. A. K.; Puschmann, H. OLEX2: a complete structure solution, refinement and analysis program. *J. Appl. Crystallogr.* **2009**, *42*, 339-341.

(8) Sheldrick, G. A short history of SHELX. *Acta Crystallogr. A* **2008**, *64*, 112-122.

(9) Sheldrick, G. Crystal structure refinement with SHELXL. *Acta Crystallogr. C* **2015**, *71*, 3-8.

(10) de Mello, J. C.; Wittmann, H. F.; Friend, R. H. An improved experimental determination of external photoluminescence quantum efficiency. *Adv. Mater.* **1997**, *9*, 230-232.

(11) Franzke, Y. J.; Holzer, C.; Andersen, J. H.; Begušić, T.; Bruder, F.; Coriani, S.; Della Sala, F.; Fabiano, E.; Fedotov, D. A.; Fürst, S.; Gillhuber, S.; Grotjahn, R.; Kaupp, M.; Kehry, M.; Krstić, M.; Mack, F.; Majumdar, S.; Nguyen, B. D.; Parker, S. M.; Pauly, F.; Pausch, A.; Perlt, E.; Phun, G. S.; Rajabi, A.; Rappoport, D.; Samal, B.; Schrader, T.; Sharma, M.; Tapavicza, E.; Treß, R. S.; Voora, V.; Wodyński, A.; Yu, J. M.; Zerulla, B.; Furche, F.; Hättig, C.; Sierka, M.; Tew, D. P.; Weigend, F. TURBOMOLE: Today and Tomorrow. *J. Chem. Theory Comput.* **2023**, *19*, 6859-6890.

(12) TURBOMOLE V7.7 2022, a development of University of Karlsruhe and Forschungszentrum Karlsruhe GmbH, 1989–2007, TURBOMOLE GmbH, since 2007; available from https://www.turbomole.org.

(13) Perdew, J. P.; Ernzerhof, M.; Burke, K. Rationale for mixing exact exchange with density functional approximations. *J. Chem. Phys.* **1996**, *105*, 9982-9985.

(14) Adamo, C.; Barone, V. Toward reliable density functional methods without adjustable parameters: The PBE0 model. *J. Chem. Phys.* **1999**, *110*, 6158-6170.

(15) Weigend, F.; Ahlrichs, R. Balanced basis sets of split valence, triple zeta valence and quadruple zeta valence quality for H to Rn: Design and assessment of accuracy. *Phys. Chem. Chem. Phys.* **2005**, *7*, 3297-3305.

(16) Dolg, M.; Stoll, H.; Preuss, H. Energy-adjusted ab initio pseudopotentials for the rare earth elements. *J. Chem. Phys.* **1989**, *90*, 1730-1734.

(17) Kaupp, M.; Schleyer, P. v. R.; Stoll, H.; Preuss, H. Pseudopotential approaches to Ca, Sr, and Ba hydrides. Why are some alkaline earth MX_2 compounds bent? *J. Chem. Phys.* **1991**, *94*, 1360-1366.

(18) Weigend, F. Accurate Coulomb-fitting basis sets for H to Rn. *Phys. Chem. Chem. Phys.* **2006**, *8*, 1057-1065.

(19) Sierka, M.; Hogekamp, A.; Ahlrichs, R. Fast evaluation of the Coulomb potential for electron densities using multipole accelerated resolution of identity approximation. *J. Chem. Phys.* **2003**, *118*, 9136-9148.

(20) Treutler, O.; Ahlrichs, R. Efficient molecular numerical integration schemes. *J. Chem. Phys.* **1995**, *102*, 346-354.

(21) Yang, X.-X.; Issac, I.; Lebedkin, S.; Kühn, M.; Weigend, F.; Fenske, D.; Fuhr, O.; Eichhöfer, A. Red-luminescent biphosphine stabilized $Cu_{12}S_6$ cluster molecules. *Chem. Commun.* **2014**, *50*, 11043-11045.
